# Increased intracellular stress responses and decreased KLF2 in adult patients with atopic dermatitis

**DOI:** 10.1016/j.cstres.2025.02.001

**Published:** 2025-02-10

**Authors:** Shuji Sugiura, Hiderou Yoshida, Hisashi Sugiura, Masami Uehara, Yasuo Sugiura, Yoshihiro Maruo, Yuji Hayashi, Takefumi Yamamoto, Takeshi Kato, Noriki Fujimoto, Jun Udagawa

**Affiliations:** 1 Department of Dermatology, Shiga University of Medical Science, Otsu, Japan; 2 Division of Anatomy and Cell Biology, Department of Anatomy, Shiga University of Medical Science, Otsu, Japan; 3 Department of Molecular Biochemistry, Graduate School of Life Science, University of Hyogo, Ako, Japan; 4 Department of Dermatology, Sugiura Dermatology Clinic, Kusatsu, Japan; 5 International Health Care Center, National Center for Global Health and Medicine, Tokyo, Japan; 6 Department of Pediatrics, Navitas Clinic, Tokyo, Japan; 7 Department of Pediatrics, Shiga University of Medical Science, Otsu, Japan; 8 Hospital Division of Diagnostic Pathology, Shiga University of Medical Science, Otsu, Japan; 9 Central Research Laboratory, Shiga University of Medical Science, Otsu, Japan

**Keywords:** Atopic dermatitis, Endoplasmic reticulum stress, Unfolded protein response, Chaperones, Heat shock protein

## Abstract

Atopic dermatitis (AD) is prone to exacerbations in response to various triggering factors and flare-ups after remission. We searched for molecules associated with relapse/exacerbation of AD among molecules with altered gene expression in the skin of patients with AD. Microarray analyses were performed on lesional and nonlesional skin of adolescent or adult patients with recalcitrant AD and healthy controls. Five chaperones involved in intracellular stress responses, namely heat shock protein family A (Hsp70) member 9 (*HSPA9*), heat shock protein 90 beta family member 1 (*HSP90B1*), calnexin (*CANX*), malectin (*MLEC*; endoplasmic reticulum-associated degradation), and heat shock protein family D (Hsp60) member 1 (*HSPD1*), were consistently upregulated in involved and uninvolved skin of patients with AD. Damage-associated molecular patterns were upregulated in involved skin. KLF transcription factor 2 (*KLF2*) was decreased in involved skin and exhibited a decreasing trend in uninvolved skin of patients with AD. CD4(+)/CD8(+) double-positive cells (1.4% of T cells) were detected in lesions with declined KLF2 levels. WNT inhibitory factor 1 (WIF1) was downregulated in involved skin. Prolactin-induced protein was upregulated in only uninvolved skin of patients with AD. We found increased intracellular stress responses and decreased expression of KLF2 in the skin of patients with AD. Multifactorial genetic diseases, such as asthma, inflammatory bowel disease, type 2 diabetes, and rheumatoid arthritis, are associated with intracellular stress. Intracellular abnormalities may also be responsible for AD. Further research on AD may incorporate enhanced intracellular stress response and the decreased expression of KLF2 into the mechanism underlying AD.

## Introduction

Atopic dermatitis (AD) is a chronic disease that affects approximately 20% of children and up to 10% of adults in high-income countries.^1^ It is associated with pruritus-induced sleep disruption, decreased work productivity, and depression and anxiety, thereby creating a health and economic burden for patients and their families.^1^ Thus, AD has important social implications.

Exacerbations of AD may be caused by various triggering factors that do not pose major problems in healthy individuals: seasonal changes, changes in environmental humidity and temperature, and physical stimuli (sweating and scratching, fatigue, overwork, and lack of sleep).^2^, ^3^

AD is a multifactorial genetic disease with many statistically significant but infrequent mutations.^4^ The pathogenesis of AD involves impaired barrier^5^ and T-cell functions.^4^ Symptoms may be caused by exogenous proteases through innate immunity or endogenous proteases (prolactin-induced protein [PIP]) present in sweat and saliva.^6^

Asthma is another multifactorial genetic disease aggravated by various stimuli (intrusion of allergic substances from the environment, chemical irritants, air pollution, cold air, stressors causing emotions, and physical exercise).^7^, ^8^

AD was initially termed neurodermatitis.^9^ However, Sulzberger *et al*.^9^ proposed a new term on the basis that patients with this disease belonged to atopic families with a high incidence of hay fever and asthma. Therefore, AD and asthma may share a common background (atopic predisposition).^9^

Intracellular stress, such as endoplasmic reticulum (ER) stress and unfolded protein response (UPR) with increased expression of chaperones, is associated with the pathogenesis of asthma.^8^, ^10^ In addition, intracellular stress is associated with multifactorial genetic diseases with many genetic mutations, such as inflammatory bowel disease (IBD),^11^, ^12^ type 2 diabetes (T2D),^13^ rheumatoid arthritis (RA),^14^ atherosclerosis, and coronary artery diseases.^13^ Numerous other human disorders (amyloidosis, cataracts, and vitiligo) are also associated with the activation of UPR and ER stress.^15^ Moreover, asthma,^9^ IBD,^11^, ^12^ T2D,^13^ and RA^14^ may benefit from treatment with chemical chaperones that reduce intracellular stress.

In this study, microarray analysis was performed to identify genes that are variably expressed in the skin of adolescent or adult patients with recalcitrant AD. Moreover, the available literature on the function of these genes was examined to elucidate the proneness of AD to flare-ups after remission.

## Results

**Fig. 1:**
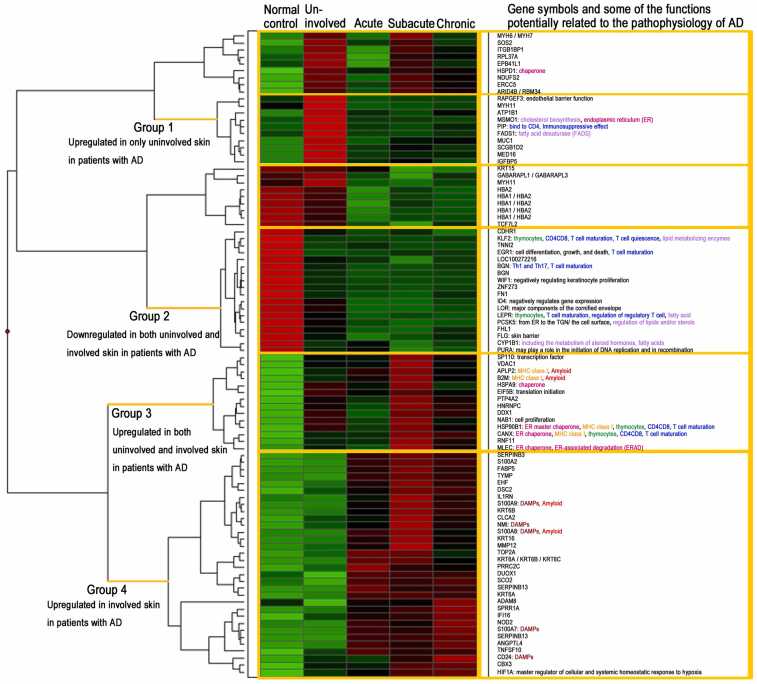
Clustering analysis of microarray data. The clustering analysis subdivided the involved skin into three groups, namely acute, subacute, and chronic as shown in Table S1 (Backgrounds of all AD patients). This analysis was subject to at least a significant difference between any two groups. Group 1: Upregulated in only uninvolved skin of patients with atopic dermatitis (AD). Group 2: Downregulated in both uninvolved and involved skin of patients with AD. Group 3: Upregulated in both uninvolved and involved skin of patients with AD. Group 4: Upregulated in involved skin of patients with AD. Among the six clusters, genes in groups 2 and 3 showing a consistent change in expression in uninvolved and involved skin represented a basic property of the skin of patients with AD. Group 2 included: *KLF2*, *LEPR*, *BGN* (promote T-cell maturation and differentiation); *WIF1* (negatively regulates keratinocyte proliferation); *EGR1* (involved in cell growth and T-cell maturation); *WIF1* and *CDHR1* (related to the WNT signal); and *FN1*, *LOR*, and *FLG* (related to structure). The mRNA expression of *FLG*, *LOR*, *LEPR*, and *BGN* was decreased in uninvolved skin, and these changes in expression were more extensive in involved skin. These findings indicated that secondary changes caused by disease activity are partly responsible for the dysfunction of these genes. Group 3 included: *HSP90B1*, *HSPA9*, *HSPD1*, and *CANX* (molecular chaperones); *CANX*, *B2M*, *HSP90B1*, and *APLP2* (major histocompatibility complex [MHC] class I-related genes); and *NAB1* (related to cell growth). The mRNA expression of many chaperones was increased in uninvolved skin, and these changes in expression were more extensive in subacute relapsing lesions with high disease activity. Based on our analysis, some genes that are highly expressed only in uninvolved skin of patients with AD (e.g., prolactin-induced protein [PIP], which exerts an immunosuppressive effect), may be essential for suppressing the onset of disease. Some functions of the genes are indicated by the following colored text: pink denotes chaperone, endoplasmic reticulum (ER), and ER-associated degradation (ERAD); purple denotes lipid, fatty acid, and sterol regulation; blue denotes T-cell-related; green denotes thymocytes; yellow denotes MHC class I; red denotes amyloid; and brown denotes DAMPs. Abbreviations used: DAMPs, damage/danger-associated molecular patterns; IHC, immunohistochemistry; TGN, trans-Golgi network; Th1, T helper 1.

Table 1 shows upregulated genes in both uninvolved and involved skin of patients with AD. Tables 2a and 2b show upregulated or downregulated genes in involved skin of patients with AD, respectively. Table 3 shows upregulated genes in only uninvolved skin of patients with AD. Clustering analysis was performed (Figure 1). The functions of these candidate genes were investigated in the literature and are described along with the gene names in Table S1. Figure S1 shows variation in gene expression of all candidate genes in individual cases.

**Table 1:** Genes shown to be upregulated in both uninvolved and involved skin in patients with AD.

We compared expression profile between skin of normal controls and both uninvolved and involved skin of patients with atopic dermatitis. Genes showing consistent expression variation from uninvolved to involved areas are shown. Among those with increased expression, five chaperones (*CANX, HSPA9, HSP90B1, HSPD1,* and *MLEC*) were found (indicated by the orange color). *CANX* and *HSP90B1* are major histocompatibility complex class I-related genes and associated T-cell maturation (Table S3). *NAB1* can inhibit *EGR1* functions such as cell proliferation, macrophage differentiation, and synaptic activation (Table S3). *HSPD1* and *MLEC* are listed because of their statistical significance (*P* < 0.05) and functional importance, although their expression is increased less than two-fold.

**Table 3:** Genes shown to be upregulated only in Uninvolved skin.

Gene name	Symbol	Probe set	Accession	Location	Uninvolved/Normal		Uninvolved/Involved	
					Fold change	*P*-value	Fold change	*P*-value
*secretoglobin, family 1D, member 2*	*SCGB1D2*	206799_at	NM_006551	chr11q13	6.0	0.047	2.6	0.012
*prolactin-induced protein*	*PIP*	206509_at	NM_002652	chr7q34	3.5	0.030	2.9	0.024
*mediator complex subunit 16*	*MED16*	221418_s_at	NM_005481	chr19p13.3	3.4	0.030	2.1	0.024
*mucin 1, cell surface associated*	*MUC1*	213693_s_at	AI610869	chr1q21	2.8	0.011	1.8	0.008
*insulin like growth factor binding protein 5*	*IGFBP5*	203426_s_at	M65062	chr2q35	2.8	0.030	1.9	0.035
*SOS Ras/Rho guanine nucleotide exchange factor 2*	*SOS2*	217576_x_at	BF692958	chr14q21	2.5	0.018	1.5	0.027
*fatty acid desaturase 1*	*FADS1*	208962_s_at	BE540552	chr11q12.2-q13.1	2.0	0.047	2.0	0.003
*zinc finger, CCHC domain containing 2*	*ZCCHC2*	219062_s_at	NM_017742	chr18q21.33	2.0	0.030	1.5	0.039

We compared expression profile between skin of normal controls and uninvolved skin of patients with atopic dermatitis (AD). We also compared expression profile between uninvolved skin and involved skin of patients with AD. *IGFBP5* induces skin fibrosis (Table S3). Subjects carrying polymorphisms of the FADS1 are known to have a lower prevalence of allergic rhinitis and atopic eczema (Table S3). PIP has two functions: 1) to suppress local and systemic contact dermatitis and 2) to promote epidermal differentiation and proliferation. Although, the role of other genes in AD has not yet been elucidated, genes that are upregulated specifically in uninvolved skin may be involved in both "preventing AD lesions from relapsing" and "causing lesion development."

**Table 4a:** Pathways analysis of genes upregulated in both uninvolved and involved skin in patients with AD.

Pathway analysis identified RNA degradation and thyroid hormone synthesis as candidates (*P < 0.05).* Protein processing in endoplasmic reticulum was a candidate, although there were no significant differences. Count: gene counts belonging to corresponding annotation term. %: percentage of genes in the list belonging to corresponding annotation term. Genes: symbols and probe ID of the genes belonging to corresponding annotation term. P value: modified Fisher's exact P-values computed through Database for Annotation, Visualization and Integrated Discovery Gene-enrichment and Functional Annotation analysis (genes with *P < 0.1* are selected. Gray areas are pathways where *P < 0.05* is not met).

**Table 4b:** Biological process analysis of genes upregulated in both uninvolved and involved skin in patients with AD.

Category	Term	Link	Count	%	*P*-value	Benjamini	Gene_1	Gene_2	Gene_3	Probe_1	Probe_2	Probe_3
GOTERM_BP_DIRECT	GO:0006457∼protein folding	Protein Folding	3	37.5	0.002	0.195	HSP90B1 (TRA1)	HSPD1	CANX	216449_X_AT	200806_S_AT	208853_S_AT
GOTERM_BP_DIRECT	GO:0034975∼protein folding in endoplasmic reticulum	Protein folding in endoplasmic reticulum	2	25	0.006	0.278	HSP90B1 (TRA1)	CANX		216449_X_AT	208853_S_AT	
GOTERM_BP_DIRECT	GO:0042026∼ protein refolding	Protein refolding	2	25	0.013	0.386	HSPA9B	HSPD1		200692_S_AT	200806_S_AT	
GOTERM_BP_DIRECT	GO:0043066∼negative regulation of apoptotic process	Negative regulation of apoptotic process	3	37.5	0.023	0.485	HSP90B1 (TRA1)	HSPA9B	HSPD1	216449_X_AT	200692_S_AT	200806_S_AT
GOTERM_BP_DIRECT	GO:0030433∼ubiquitin-dependent ERAD pathway	Ubiquitin-dependent ERAD pathway	2	25	0.032	0.550	HSP90B1 (TRA1)	CANX		216449_X_AT	208853_S_AT	
GOTERM_BP_DIRECT	GO:0036503∼ERAD pathway	ERAD pathway	2	25	0.041	0.581	HSP90B1 (TRA1)	CANX		216449_X_AT	208853_S_AT	

The analysis resulted in processes associated with protein folding and refolding and pathways associated with ERAD.

Abbreviation used: ERAD, endoplasmic reticulum-associated degradation.

### Increased intracellular stress responses

In microarray analysis (Table 1), upregulated genes in both uninvolved and involved skin included multiple molecular chaperones, namely calnexin (*CANX*),^16^ heat shock protein family A (Hsp70) member 9 (*HSPA9*),^17^ heat shock protein 90 beta family member 1 (*HSP90B1*),^17^ heat shock protein family D (Hsp60) member 1 (*HSPD1*),^17^ and malectin (*MLEC*).^18^ *CANX* and *HSP90B1* are also major histocompatibility complex (MHC) class I-related genes as well as chaperones. Pathways analysis (Table 4a) and biological process analysis (Table 4b) were performed on the genes shown in Table 1. The analysis revealed the involvement of processes associated with protein folding and refolding and pathways associated with ER-associated degradation (ERAD).

**Table 2a:** Genes shown to be upregulated in involved skin in patients with AD.

Gene name	Symbol	Probe set	Accession	Location	Involved/Normal		Involved/Uninvolved	
					Fold change	*P*-value	Fold change	*P*-value
*S100 calcium binding protein A8*	*S100A8*	202917_s_at	NM_002964	chr1q21	28.6	0.002	24.6	0.000
*keratin 6A, type II*	*KRT6A*	209125_at	J00269	chr12q13.13	7.3	0.002	4.4	0.000
*S100 calcium binding protein A7*	*S100A7*	205916_at	NM_002963	chr1q21	6.5	0.002	6.1	0.000
*serpin peptidase inhibitor, clade B (ovalbumin), member 13*	*SERPINB13*	211361_s_at	AJ001696	chr18q21.33	6.3	0.013	6.3	0.002
*keratin 6A, type II /// keratin 6B, type II /// keratin 6C, type II*	*KRT6A /// KRT6B /// KRT6C*	214580_x_at	AL569511	chr12q13.13	5.5	0.002	4.4	0.000
*calnexin*	*CANX*	208853_s_at	L18887	chr5q35	4.4	0.003	1.6	0.239
*keratin 16, type I*	*KRT16*	209800_at	AF061812	chr17q21.2	4.2	0.002	3.4	0.000
*small proline-rich protein 1A*	*SPRR1A*	213796_at	AI923984	chr1q21-q22	3.4	0.002	2.7	0.001
*keratin 6B, type II*	*KRT6B*	213680_at	AI831452	chr12q13.13	3.4	0.004	2.4	0.004
*S100 calcium binding protein A2*	*S100A2*	204268_at	NM_005978	chr1q21	3.0	0.004	2.6	0.004
*fatty acid binding protein 5 (psoriasis-associated)*	*FABP5*	202345_s_at	NM_001444	chr8q21.13	3.0	0.003	2.7	0.000
*ets homologous factor*	*EHF*	219850_s_at	NM_012153	chr11p12	2.9	0.002	2.0	0.000
*voltage-dependent anion channel 1*	*VDAC1*	217140_s_at	AJ002428	chr5q31	2.7	0.003	1.3	0.095
*nucleotide-binding oligomerization domain containing 2*	*NOD2*	220066_at	NM_022162	chr16q21	2.6	0.022	2.7	0.011
*heat shock protein 90 kDa beta (Grp94), member 1*	*HSP90B1*	216449_x_at	AK025862	chr12q24.2-q24.3	2.5	0.005	1.0	0.659
*desmocollin 2*	*DSC2*	204750_s_at	BF196457	chr18q12.1	2.5	0.002	1.6	0.001
*interferon, gamma-inducible protein 16*	*IFI16*	208965_s_at	BG256677	chr1q22	2.5	0.002	1.5	0.001
*heterogeneous nuclear ribonucleoprotein C (C1/C2)*	*HNRNPC*	212626_x_at	AA664258	chr14q11.2	2.4	0.007	1.2	0.106
*serpin peptidase inhibitor, clade B (ovalbumin), member 3*	*SERPINB3*	209720_s_at	BC005224	chr18q21.3	2.4	0.002	2.0	0.000
*N-myc (and STAT) interactor*	*NMI*	203964_at	NM_004688	chr2q23	2.3	0.002	1.7	0.002
*topoisomerase (DNA) II alpha*	*TOP2A*	201291_s_at	AU159942	chr17q21.2	2.3	0.003	1.8	0.001
*beta-2-microglobulin*	*B2M*	216231_s_at	AW188940	chr15q21.1	2.2	0.010	1.3	0.012
*S100 calcium binding protein A9*	*S100A9*	203535_at	NM_002965	chr1q21	2.2	0.002	2.0	0.000
*serpin peptidase inhibitor, clade B (ovalbumin), member 13*	*SERPINB13*	217272_s_at	AJ001698	chr18q21.33	2.2	0.004	2.1	0.001
*chromobox homolog 3*	*CBX3*	201091_s_at	BE748755	chr7p15.2	2.1	0.022	1.1	0.141
*NGFI-A binding protein 1*	*NAB1*	208047_s_at	NM_005966	chr2q32.3-q33	2.1	0.019	−1.0	0.220
*excision repair cross-complementation group 5*	*ERCC5*	202414_at	NM_000123	chr13q33	2.1	0.022	−1.1	0.659
*hypoxia inducible factor 1, alpha subunit (basic helix-loop-helix transcription factor)*	*HIF1A*	200989_at	NM_001530	chr14q23.2	2.0	0.007	1.3	0.070
*amyloid beta (A4) precursor-like protein 2*	*APLP2*	208703_s_at	BG427393	chr11q24	1.8	0.015	1.2	0.106

**Table 2b:** Genes shown to be downregulated in involved skin in patients with AD.

Gene name	Symbol	Probe set	Accession	Location	Involved/Normal		Involved/Uninvolved	
					Fold change	*P*-value	Fold change	*P*-value
*loricrin*	*LOR*	207720_at	NM_000427	chr1q21	−5.7	0.006	−3.2	0.001
*versican*	*VCAN*	221731_x_at	BF218922	chr5q14.3	−5.1	0.037	−1.9	0.202
*fibronectin 1*	*FN1*	211719_x_at	BC005858	chr2q34	−4.5	0.037	−1.7	0.352
*chemokine (C-X-C motif) ligand 14*	*CXCL14*	218002_s_at	NM_004887	chr5q31	−4.1	0.027	−1.2	0.806
*filaggrin*	*FLG*	215704_at	AL356504	chr1q21.3	−4.0	0.044	−1.9	0.039
*leptin receptor*	*LEPR*	209894_at	U50748	chr1p31	−3.9	0.010	−1.9	0.014
*WNT inhibitory factor 1*	*WIF1*	204712_at	NM_007191	chr12q14.3	−3.7	0.002	−1.3	0.035
*troponin I type 2 (skeletal, fast)*	*TNNI2*	206393_at	NM_003282	chr11p15.5	−3.0	0.010	−1.1	0.556
*biglycan*	*BGN*	213905_x_at	AA845258	chrXq28	−3.0	0.009	−1.3	0.259
*uncharacterized LOC100272216*	*LOC100272216*	213089_at	AU158490	---	−2.7	0.009	−1.4	0.117
*fibronectin 1*	*FN1*	210495_x_at	AF130095	chr2q34	−2.7	0.037	−1.3	0.303
*cadherin-related family member 1*	*CDHR1*	213369_at	AI825832	chr10q23.1	−2.7	0.015	−1.4	0.141
*four and a half LIM domains 1*	*FHL1*	201540_at	NM_001449	chrXq26	−2.6	0.019	−1.5	0.070
*proprotein convertase subtilisin/kexin type 5*	*PCSK5*	205559_s_at	NM_006200	chr9q21.3	−2.6	0.007	−1.6	0.033
*inhibitor of DNA binding 4, dominant negative helix-loop-helix protein*	*ID4*	209291_at	AW157094	chr6p22.3	−2.3	0.009	−1.5	0.021
*transcription factor 7-like 2 (T-cell specific, HMG-box)*	*TCF7L2*	212762_s_at	AI375916	chr10q25.3	−2.2	0.019	−1.9	0.070
*biglycan*	*BGN*	201261_x_at	BC002416	chrXq28	−2.2	0.013	−1.2	0.117
*zinc finger protein 273*	*ZNF273*	215239_x_at	AU132789	chr7q11.21	−2.2	0.022	−1.2	0.492
*dual specificity phosphatase 1*	*DUSP1*	201041_s_at	NM_004417	chr5q34	−2.2	0.044	−1.0	1.000
*Kruppel-like factor 2*	*KLF2*	219371_s_at	NM_016270	chr19p13.11	−2.0	0.013	−1.1	0.303

Genes shown to be upregulated or downregulated in involved skin in patients with atopic dermatitis (AD). We compared expression profile between skin of normal controls and involved skin of patients with AD. We also compared expression profile between uninvolved skin and involved skin of patients with AD. Genes with variable expression in the involved lesions are shown. Among those that are up regulated are several damage/danger-associated molecular patterns *(NMI, S100A7, S100A8, S100A9*). *HIF1A* is master regulator of cellular and systemic homeostatic response to hypoxia. *APLP2* and *B2M* is MHC class I rerated genes. *APLP2* is listed because of their statistical significance (*P < 0.05)* and functional importance, although their expression is increased less than two-fold (a). The downregulated genes include *FN1* as well as *LOR* and *FLG*, which have been already well-known as candidate genes for AD. The downregulated genes also include a number of those associated with T-cell maturation and differentiation (*KLF2, LEPR, BGN*). *WIF1*, which negatively regulates keratinocyte proliferation, and purine rich element binding protein A, which is associated with DNA replication, are also down regulated. *PCSK5* is also downregulated and known to be involved in squamous differentiation of human nasal epithelial cells (b).

**Fig. 2:**
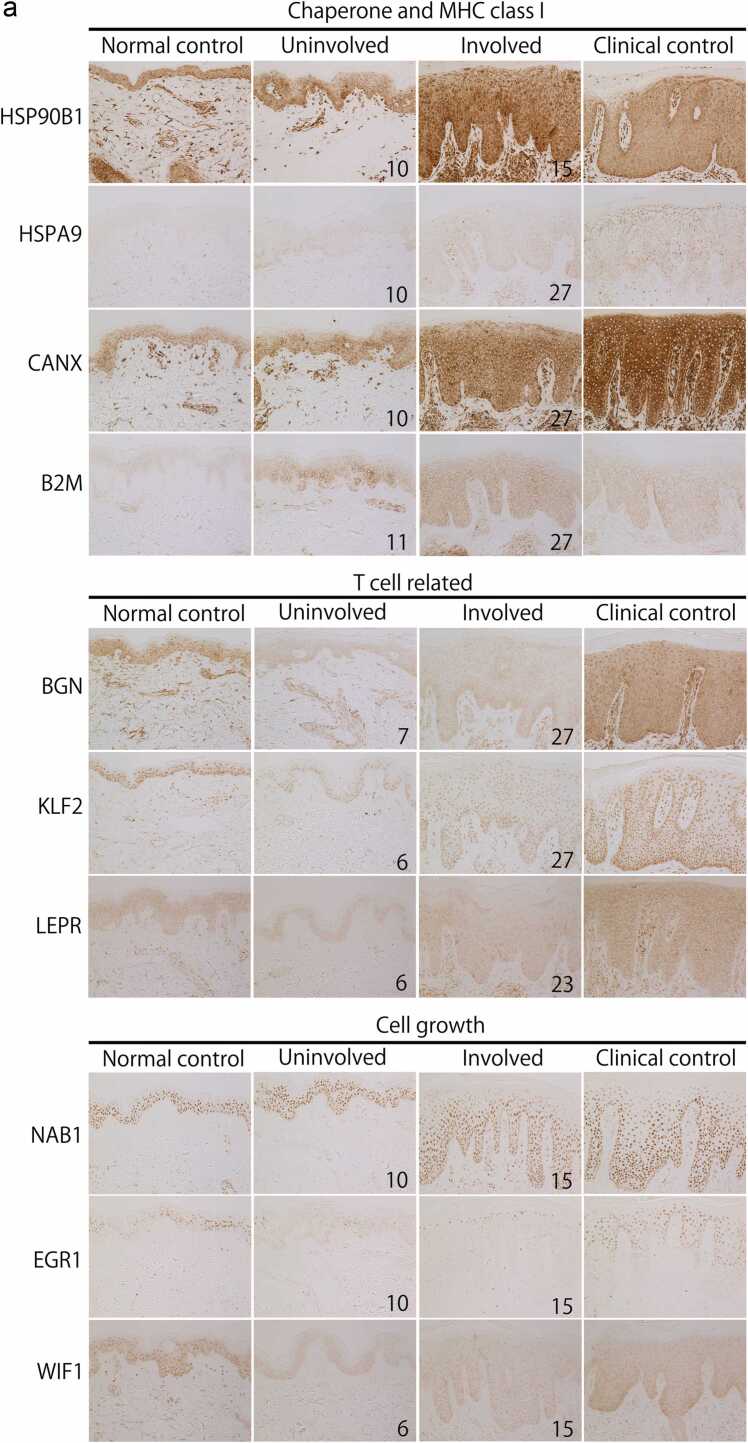

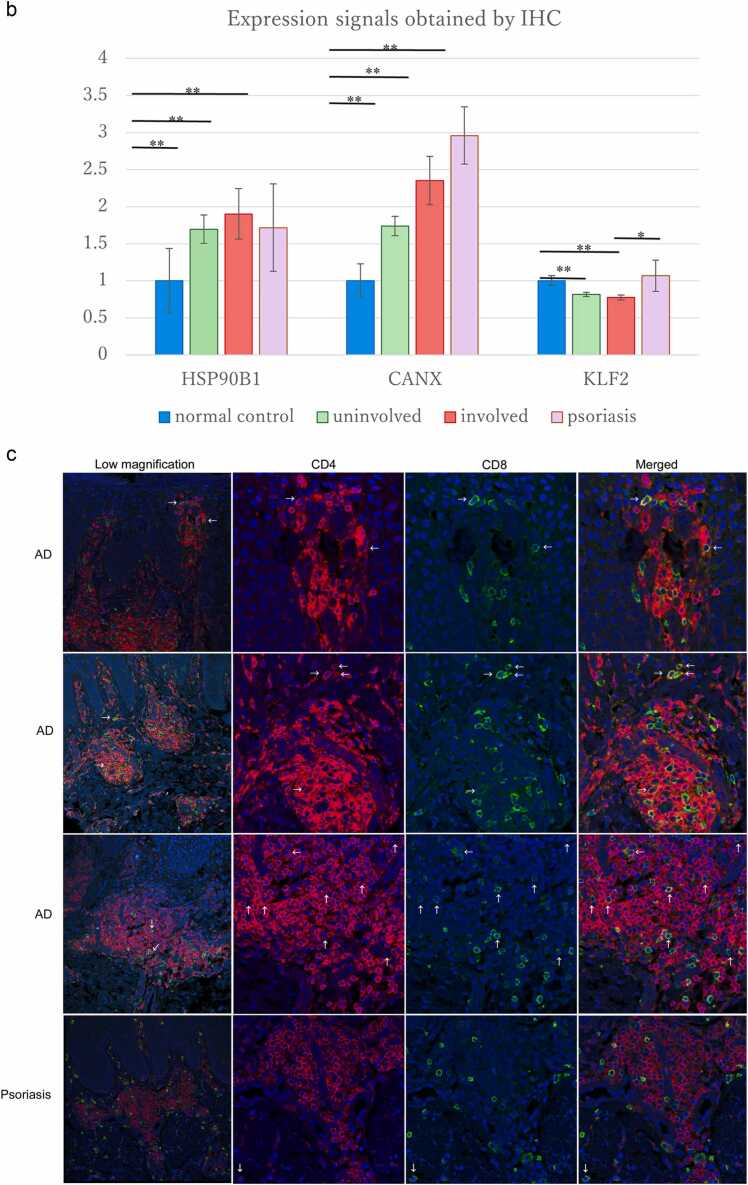
(a) Histopathological findings. Representative case of immunohistochemical staining. Concerning HSP90B1, CANX, and B2M, the staining intensity of epidermal cells was increased in uninvolved and involved skin of patients with atopic dermatitis (AD) *versus* normal controls. Regarding BGN, KLF2, and LEPR, the staining properties of epidermal cell nuclei, as well as the number of dermal infiltrating cells and vascular endothelial cells were reduced in both nonlesioned and lesioned areas of patients with AD *versus* normal and clinical controls (psoriasis). Concerning EGR1 and WIF1, the staining intensity of epidermal cells was decreased in uninvolved and involved areas of patients with AD *versus* normal and clinical controls. Increased B2M staining was observed in epidermal, dermal endothelial, and infiltrating cells in both nonlesioned and lesioned areas of patients with AD *versus* normal and clinical controls. The nuclei of epidermal cells expressed NAB1 or EGR1, but not both. Focusing on the staining property of intracellular organelles, HSP90B1, HSPA9, and CANX (chaperones) was positively stained in the cytoplasm of epidermal cells. Moreover, the endoplasmic reticulum (ER) distributing area around the nuclear membrane was strongly stained. KLF2, NAB1, EGR1, and WIF1, which act as transcription factors, were clearly stained in the nuclei of epidermal cells. Original magnification: x 200. The numbers in the image represent shows the case number described in the Table S3 (backgrounds of all AD patients) of Supplementary material. (b) Expression signals of HSP90B1, CANX, and KLF2 were obtained by IHC. When quantified using Fiji,^19^ the expression signals of HSP90B1 and CANX obtained by IHC were significantly increased in both uninvolved (*P* < 0.01, *P* < 0.01) and involved skin in atopic dermatitis (*P* < 0.01, *P* < 0.01), respectively, compared with the normal control ([a]; Table S2). HSP90B1 and CANX were significantly increased in psoriasis as clinical control compared with the normal control. Protein levels of molecular KLF2 quantified by Fiji^19^ were significantly decreased in both uninvolved and involved skin (*P* < 0.01) compared with the normal control. Protein levels were significantly decreased (*P* < 0.05) in involved skin compared with the clinical control. HSP90B1 expression is upregulated in psoriasis; in this study, we observed that CANX expression is also upregulated. Of note, we did not find any reports indicating the presence of CANX in lesions of psoriasis *P < 0.05, **P < 0.01; bars accompanying the graphs represent standard deviations. (c) Fluorescent staining. Three representative lesions of AD are shown in the double-staining low-magnification panel (×200), high-magnification CD4 panel, CD8 panel, and double-staining “merged” panel (×400). CD4(+) T cells are shown as Cy3 (red). CD8(+) T cells are shown as FITC (green). The nuclei of epidermal cells and infiltrating cells are shown in blue (DAPI). CD4(+)/CD8(+) double-positive T cells are indicated by arrows and appear yellow both in the “low-magnification” and “merged” panels. CD4(+)/CD8(+) double-positive cells were mean 1.4%, standard deviation 0.7 in AD and mean 0.5%, standard deviation 0.3 in psoriasis. The number of CD4(+)/CD8(+) double-positive T cells was lower in psoriasis than in AD, and the cells stained as granules (*P* < 0.05). Abbreviations used: CANX, calnexin; DAPI, 4′,6-diamidino-2-phenylindole; FITC, fluorescein isothiocyanate; HSP90B1, heat shock protein 90 beta family member 1; IHC, immunohistochemistry; KLF2, KLF transcription factor 2.

When quantified using Fiji,^19^ the expression signals of HSP90B1 and CANX obtained by immunohistochemistry (IHC) were significantly increased in both uninvolved (*P* < 0.001, *P* < 0.01) and involved skin in AD (*P* < 0.001, *P* < 0.01), respectively, compared with the normal control (Figure 2(b); Table S2). HSP90B1 and CANX were significantly increased in psoriasis as clinical control compared with the normal control. Protein levels of HSPA9 showed increased expression in some individuals; however, this observation was not consistent with the microarray data.

Upregulated genes in involved skin included damage/danger-associated molecular patterns (DAMPs), namely N-myc and STAT interactor (*NMI*), S100 calcium binding protein A7 (*S100A7*), and particularly S100 calcium binding protein A8 (*S100A8*) and S100 calcium binding protein A9 (*S100A9*) (Table 2a).^20^ Hypoxia-inducible factor 1 (*HIF1A*) and MHC I-related genes amyloid beta precursor like protein 2 (*APLP2*) and beta-2-microglobulin (*B2M*) were also upregulated.

### Downregulation of KLF transcription factor 2 (KLF2) and immature T-cell infiltrate

Downregulated genes in involved skin are shown in Table 2b. *KLF2*,^21^, ^22^ leptin receptor (*LEPR*), and biglycan (*BGN*) (Table S1) genes promote T-cell maturation and differentiation. Protein levels of molecular KLF2 quantified by Fiji^19^ were significantly decreased in both uninvolved and involved skin (*P* < 0.01) compared with the normal control. Protein levels in involved skin were significantly decreased (*P* < 0.05) compared with those recorded in the clinical control (Figure 2(b)). *KLF2*^21^ induces the transformation of CD4(+)/CD8(+) double-positive cells into CD4(+) or CD8(+) single-positive cells during T-cell maturation in the thymus. *KLF2* and *LEPR* (Table S1) are associated with the regulation of regulatory T (Treg) cells. *CANX* and *HSP90B1* are also associated with T-cell maturation (Table S1). Leptin^23^ and *BGN* (Table S1) tilt the T helper 1/T helper 2 (Th1/Th2) balance toward Th1. Fatty acid binding protein 5 (*FABP5*) (Table 1) is a possible biomarker in atopic march and induces Th17 polarization.^24^

Proteins related to T-cell maturation and differentiation (BGN, KLF2, LEPR) in involved and uninvolved areas were decreased (Figure 2(a); Table S2). Regarding BGN, KLF2, and LEPR, the staining properties of epidermal cell nuclei, as well as dermal infiltrating cells and vascular endothelial cells tended to be decreased in both nonlesioned and lesioned areas of patients with AD *versus* normal and clinical controls (psoriasis).

In the dermis of AD lesions, KLF2 was also expressed on CD3(+) cells (Figure S2). In AD lesions with declined KLF2 expression, CD4(+)/CD8(+) double-positive cells were detected among the dermal infiltrating T cells (Figure 2(c)). Significantly more CD4(+)/CD8(+) double-positive cells were observed in AD than in psoriasis (mean: 1.4% [standard deviation: 0.7%] vs. 0.5% [standard deviation 0.3%], respectively; *P* < 0.05).

### Cell proliferation, lipid and sterol metabolism

NGFI-A binding protein 1 (*NAB1*) can inhibit early growth response 1 (*EGR1*) function, such as cell proliferation, macrophage differentiation, and synaptic activation (Table S1). As shown in Table 2b, WNT inhibitory factor 1 (WIF1) was downregulated in involved skin (Table S1); WIF1 negatively regulates keratinocyte proliferation.^25^ Proteins linked to cell growth (NAB1, EGR1, WIF1) in involved and uninvolved areas were decreased (Figure 2(a); Table S2).

Proteins related to DNA replication, such as purine rich element binding protein A (Table S1), and proteins linked to structure, that is, fibronectin 1 (FN1), loricrin cornified envelope precursor protein (LOR), and filaggrin (FLG) (Table S1), were downregulated in involved skin (Table 2b).

As shown in Table 2b, the expression of proprotein convertase subtilisin/kexin type 5 (*PCSK5*) (Table S1 and Discussion in the Supplementary material) was involved in lipid and sterol metabolism.

### Genes upregulated only in uninvolved skin

Genes upregulated only in uninvolved skin of patients with AD included Son of sevenless homolog 2 (Drosophlia) (*SOS2*), *PIP* (binding to CD4, exerting an immunosuppressive effect),^26^ mediator complex subunit 16 (*MED16*), mucin 1, cell surface associated (*MUC1*), insulin-like growth factor binding protein 5 (*IGFBP5*), and fatty acid desaturase 1 (*FADS1*) (Table 3). *IGFBP5* induces skin fibrosis (Table S1). Individuals carrying polymorphisms of the *FADS1* are linked to a lower prevalence of allergic rhinitis and atopic eczema (Table S1).

### Increased expression in the subacute phase in the clustering analysis

The clustering analysis subdivided the involved skin into three groups, namely acute, subacute, and chronic (Figure 1). This analysis was subject to at least a significant difference between any two groups. HSPA9, HSP90B1, CANX, and MLEC were enhanced in the subacute phase, while HSPD1 was particularly enhanced in uninvolved and subacute lesions. Among DAMPs, S100A8 and S100A9 were enhanced in the subacute phase, while S100A7 and CD24 were particularly enhanced in the chronic phase. Amyloid-related APLP2, B2M, S100A9, and S100A8 were also most enhanced in the subacute phase.

## Discussion

Our previously published microarray data^27^ were limited to those associated with keratinization and 1q.21. In this study, we analyzed candidate genes with more than two-fold changes in expression and significant differences (*P* < 0.05) to screen for candidate genes more broadly related to the pathogenesis of AD. We also performed IHC and immunofluorescence staining. Several microarray analyses of AD have been previously performed.^28^ Ghosh *et al*.^28^ integrated and analyzed previous data, revealing several candidate genes. Some of our data were consistent with previous results. Regarding barrier function, FLG, LOR, keratin 6, and KRT16 downregulation was commonly observed. Regarding inflammation, S100A7, S100A8, and S100A9 upregulation was commonly noted. Regarding lipid metabolism, FADS1 upregulation was also recorded. The most important new findings, which add to these previous microarray data, are the increased expression of many chaperones and downregulation of KLF2 in AD. Herein, we discussed primarily “downregulation of KLF2 and abnormal T-cell maturation” and secondarily “causes of increased expression of chaperones and intracellular abnormalities.”

Prior to these two main points, we briefly discuss DAMPs and WIF1. Chronic inflammation can stimulate the secretion of DAMPs, which promote pathological inflammatory responses.^29^ The vicious cycle of DAMP production and inflammation might play a pathogenic role in inflammatory diseases.^29^ The expression of DAMPs in AD may contribute to the development of eczema and the spread of skin lesions to the surrounding area. Moreover, the decreased expression of WIF1 (Table S1), which negatively regulates keratinocyte proliferation,^25^ may cause uncontrolled epidermal proliferation in AD. These results suggest that homeostasis is partially declined in the skin of patients with AD. In addition to the abovementioned genes, the present microarray analysis revealed changes in the expression of various genes involved in immunity, cell division and proliferation, lipid metabolism, etc. The role of these candidate genes found in our study is discussed in the Supplementary material.

### Downregulation of KLF2 and abnormal T-cell maturation

KLF2 plays a role in the transformation of CD4(+)/CD8(+) double-positive T cells to CD4(+) or CD8(+) single-positive T cells in the thymus.^21^ The microarray analysis indicated that there is a constant decrease in KLF2 in AD (Table 2b). KLF2 showed a decreasing trend in uninvolved skin: no significant difference between uninvolved and normal control (−1.9 folds, *P* = 0.073), and significant difference between involved and normal control (−2.0 folds, *P* = 0.013).

Decreased KLF2 protein expression was also observed in dermal cells other than the epidermis of patients with AD (Figure 2(a)). We have not verified the reduced expression of KLF2 in the thymus of patients with AD. CD4+CD8+ immature T cells were present in the dermal infiltrate of patients with AD; hence, we speculate that KLF2 expression is reduced in the thymus.

Using a fluorescence-activated cell sorter, CD4(+)/CD8(+) double-positive T cells were detected only from T-cell lines established from AD skin lesions, but not from normal skin or AD peripheral blood.^30^ Using immunofluorescence, we directly identified CD4(+)/CD8(+) double-positive T cells infiltrating the dermis in skin tissues obtained from patients with AD (Figure 2(c)). CD4(+)/CD8(+) double-positive cells in AD lesions may arise from the decreased expression of KLF2.

*CANX* and *HSP90B1*, which transform CD4(−)/CD8(−) double-negative T cells into CD4(+)/CD8(+) double-positive T cells in the thymus (Table S1), are upregulated in skin lesions of AD (Table 1). The upregulation of *CANX* and *HSP90B1* may contribute to the response to aberrant proteins and the increased responsiveness to the downregulation of *KLF2*. Moreover, *KLF2* serves to restrain T follicular helper cell generation and enhances Th1 differentiation.^31^ Leptin regulates the Th1/Th2 balance^23^ (Table S1). The expression of *LEPR* (nutritional status of Treg cells; Table S1) and *BGN* (recruitment of Th1 and Th17; Table S1) was also downregulated in AD (Table 2b), suggesting impairment of the maturation process of T cells. Hence, immature lymphocytes may circulate throughout the body of patients with AD.

Treg cells were decreased in AD lesions, and the AD severity score was negatively correlated with the percentage of Treg cells.^32^ *KLF2* is necessary for the generation of peripheral induced Treg cells, but not thymus-derived Tregs.^22^ It also controls naive Treg migration patterns *via* regulation of homeostatic and inflammatory homing receptors. Moreover, KLF2-deficient Tregs are unable to efficiently migrate to secondary lymphoid organs.^33^ Drugs that limit KLF2 proteolysis during T-cell activation enhance induced Treg cell development.^22^ Our data showing that KLF2 expression is decreased in the lesions of AD suggest that drugs with similar effects may also improve the pathophysiology of AD.

### Genes upregulated only in uninvolved skin

PIP expression was upregulated only in nonlesional areas (Table 3). PIP binds to CD4 and inhibits the CD4-HLA-DR interaction,^34^ induces differentiation of naive T cells into CD4(+) CD25(+) forkhead box P3(+) (FOXP3(+)) Treg cells,^35^ and exerts both local and systemic immunosuppressive effects in mouse contact dermatitis.^26^ *PIP* has other functions, promoting epidermal differentiation and proliferation.^6^ Therefore, such genes that are specifically upregulated in uninvolved skin may be involved in “preventing AD lesions from relapsing” and “causing lesion development.”

### Increased expression of chaperones in AD

The microarray and IHC analyses showed that chaperones in multiple intracellular regions and MHC class I-related genes are consistently upregulated from nonlesion to lesion skin of patients with AD (Figure 2(a)). Pathway and biological process analyses of these genes suggested an association with pathways linked to protein folding and ERAD (Table 4a, 4b). During the secretory process of proteins, abnormal polypeptides are recognized and returned to the cytoplasm for degradation (i.e., ERAD).^36^ Following the abnormal accumulation of proteins in the cytoplasm or mitochondria, the transcription of *HSPA9* (a typical heat shock protein) is upregulated by the heat shock factor–heat shock element system.^37^ Following the abnormal accumulation of proteins in the ER, the expression of ER chaperones (*HSP90B1*/*GRP94*, *CANX*) is upregulated.^38^, ^39^

According to our microarray data, there was no significant difference in expression; nevertheless, activating transcription factor 6 beta expression was 1.2-fold higher (*P* = 0.073) in uninvolved/normal skin and 1.1-fold higher (*P* = 0.109) in involved/normal skin (Gene Expression Omnibus accession number: GSE174582). ATF6 is known to activate HSP90B1.^40^

Thus, the abnormality in AD may not be limited to the cytoplasm but extends to various organelles. Proteins with reported amino acid sequence abnormalities in AD are also distributed in various organelles, including the cytoplasm and plasma membrane.^3^ Several previously reported genetic mutations^41^ may be associated with minor structural or functional abnormalities of proteins. Hence, we hypothesize that, in AD, there is a constant and persistent stress in cells, resulting in a constant state of overload.

In our study, multiple pathways suggestive of intracellular abnormalities in AD (DAMPs *S100A7*, *A8*, *A9*, and *NMI*), MHC class I-related genes (*APLP2*, *B2M*, *HSP90B1*, *CANX*) and ERAD (*MLEC*)^18^ were enhanced (Figure 2(a)).

In AD, the constant overexpression of chaperones and MHC class I-related genes (MHC class I-related genes correspond to intracellular antigens) suggests that the intracellular stress response system is operating suboptimally. Consequently, stress tolerance may be lower in patients with AD *versus* healthy individuals. Therefore, we hypothesized that weaker internal and external stimuli will easily cause skin symptoms because the intracellular load exceeds the processing capacity.

Diseases associated with intracellular stress responses (UPR and ER stress response) include asthma,^8^, ^10^ IBD,^11^, ^12^ T2D,^13^ RA,^14^ and coronary artery diseases.^13^ Disruption of intracellular homeostasis contributes to the pathogenesis of these diseases. Asthmatic conditions are exacerbated by stimuli (oxidative stress, pathogenic infections, and allergen exposure).^7^, ^8^ These stimuli may induce ER stress and activate the UPR, leading to activation of various inflammatory responses and dysregulation of innate immune function in the airways.^8^ In IBD, deregulation of ER stress and UPR signaling in the intestinal epithelium may mediate the action of genetic or environmental factors driving colitis in experimental animals and patients with IBD.^12^ In T2D, ER stress causes β-cell damage and death.^13^ The relationship between the pathophysiology of AD and the intracellular stress response remains unresolved; however, the relationship between the pathophysiology of diabetes and ER stress, for example, has been reported by Oakes.^13^

The involvement of ER stress in the pathogenesis of asthma is discussed in detail in the review conducted by Miao.^42^ As stated by Miao, house dust mite (HDM) stimulation induces ER stress through the ATF6 and inositol-requiring enzyme 1 pathways. The ER chaperones glucose-regulated protein, 78 kDa (GRP78), GRP94 (HSP90B1), and ER-resident protein 57 are also markedly upregulated by HDMs.^43^ *Dermatophagoides farinae* induce ER stress by activating interleukin-25.^44^ Tauroursodeoxycholic acid effectively inhibits apoptosis partly by modulating the PRKR-like ER kinase-eukaryotic translation initiation factor 2 alpha ER stress pathway and the AKT pathway,^45^, ^46^ and reduces HDM-mediated asthma by inhibiting ER stress^47^ The ER stress blocker 4-phenylbutyric acid can reduce the expression of nuclear factor-kappaB, resulting in downregulation of the expression of Th2 cytokines (IL-4, IL-5, IL-13) and airway inflammatory response factors (IL-1β, tumor necrosis factor-alpha, interferon-gamma).^48^

Similarly, disruption of intracellular homeostasis may contribute to the pathogenesis of AD. Deposition of amyloid (the end product of abnormal proteins) is observed in the chronic phase of IBD, RA, T2D, and AD.

We could not determine whether the increased expression of chaperones causes AD or induces a parallel effect. However, we consider two possibilities. Firstly, intracellular stress (abnormal proteins) causes AD, followed by the upregulation of chaperones. Secondly, excessive expression of intracellular stress response systems (chaperones) causes AD. Serum levels of Hsp90 and anti-Hsp90 immunoglobulin E autoantibodies are significantly elevated in patients with AD.^49^ In addition, suppression of Hsp90 improved inflammation in models of AD.^50^, ^51^ Hence, AD may be caused by overactivation of the intracellular stress response.

HSP90B1 expression is upregulated in psoriasis.^52^ In this study, we observed that CANX expression is also upregulated.

### Clinical implication

Clinicians recognize that avoiding the aggravation of AD is important in the treatment of AD. The new finding of increased intracellular stress response in AD may be part of an unexplained mechanism between “internal and external stress” and “exacerbation of AD.” Increased intracellular stress response may help to explain the clinical notion that “it is not good to aggravate AD” in the future.

There are many studies incorporating intracellular stress responses into therapy; in particular, Hsp90 inhibitors are being actively investigated as therapeutic agents for a variety of diseases, including cancer.^53^ For inflammatory diseases, compound 54, reported by Jiang *et al*.^54^ to selectively inhibit HSP90B1, has also been found to be effective in a mouse model of ulcerative colitis. *HSP90B1* expression was also upregulated in the present microarray analysis; thus, *HSP90B1* may be a therapeutic target in AD.

### Further perspectives

Our findings are in agreement with the established concept regarding AD. As in asthma^55^ and diabetic nephropathy,^56^ ER stress in AD may also be regulated in association with IL-33. Further research on AD may incorporate the enhanced intracellular stress response into the mechanism underlying AD.

### Limitations

Although the present study included a large number of samples, our data are based on microarrays performed 20 years ago. The patients included in this analysis had recurrent and recalcitrant skin lesions. Therefore, the genes expressed are diverse. These data may include unimportant expression changes. In addition, compared with single cell RNA sequencing and spatial transcriptomics, the accuracy of this approach is low and proteomics analysis is not yet available. Future analyses using these newer technologies may elucidate the detailed pathophysiology of AD, such as the characteristics of relapse-prone areas.

### Conclusions

Increased intracellular stress response and decreased KLF2 may be involved in the pathogenesis of AD.

## Materials and methods

### Patients

This study involved 22 adolescent or adult patients with AD (17 men and five women; mean age: 28 years), who had not received topical or systemic corticosteroids for ≥1 month before the examination, and four healthy controls (three men and one woman; mean age: 34 years). All patients provided informed consent for their participation and fulﬁlled the diagnostic criteria established by Williams *et al*.^57^ The study protocol was approved by the Ethics Committee of Shiga University of Medical Science (Shiga, Japan) (approval number: G2011-135).

### Biopsy

Biopsy was performed to obtain 23 involved and seven uninvolved skin specimens from patients with AD. Normal skin samples were obtained from the controls. Table S3 and Figure S3 show background information and representative clinical features of patients, respectively. The biopsy specimens were divided into two parts; one part was sectioned using a cryostat for RNA extraction, while the other was fixed in 10% neutral-buffered formalin, embedded in paraffin, and subsequently subjected to IHC analysis.

### RNA preparation, DNA microarray, and pathway analysis

The present study reexamined the data presented by Sugiura *et al*.^27^ The microarray analysis was performed as previously described (Supporting Information). In the previous study, 22 cases were analyzed; of those, 17 cases with immunoglobulin E ≥2000 were selected. In the past study, cornified envelope-related genes in the 1q21 region with at least a five-fold difference in expression and keratinization-related genes in non-1q21 regions were selectively reported. In the present study, we included all 22 cases, and genes with at least a two-fold difference in expression with significant difference (*P* < 0.05) in all genomic regions were included. The National Center for Biotechnology Information Gene Expression Omnibus accession number is GSE174582. Pathway analysis was performed using Database for Annotation, Visualization and Integrated Discovery (https://david.ncifcrf.gov/home.jsp). Microarray and pathway analyses were performed by Hayao Ebise MSc of Sumitomo Pharma Co., Ltd. (Osaka, Japan).

### IHC

We examined 30 specimens of AD, 10 normal skin samples (six were not included in the microarray study) from healthy individuals, and 5–10 psoriatic lesions with psoriasis vulgaris (not included in the microarray study) as clinical controls. Ten candidate genes determined by the microarray analysis, that is, *HSP90B1*, *HSPA9*, *CANX*, *B2M*, *BGN*, *KLF2*, *LEPR*, *NAB1*, *EGR1*, and *WIF1* were analyzed using IHC. IHC was performed using an automated Roche Ventana BenchMark XT staining system (Ventana Medical Systems/Roche, Tucson, AZ, USA). Details of the antibodies used are provided in the Supplementary material. Expression signals of HSP90B1, CANX, and KLF2 obtained by IHC were quantified using Fiji.^19^ Measurements were performed on epidermal cells. Staining intensity was measured for HSP90B1 in whole cells, CANX in perinuclear area, and KLF2 in nuclei.

### Immunofluorescence study for CD4/CD8 double staining

Double staining of CD4/CD8 was observed through confocal laser scanning microscopy (Leica TCS SP8 X; Leica, Wetzlar, Germany). We selected 12 skin tissues from 12 AD cases with decreased KLF2 expression and 10 skin tissues from 10 psoriasis vulgaris patients without decreased KLF2 expression (all confirmed by IHC). The CD4 and CD8 antibodies and secondary antibodies used in this experiment are described in the Supplementary material.

## Ethics statement

All patients signed written informed consent.

## Funding and support

H.Y. received funding from JSPS KAKENHI (Grant number: 19K06645); Grant-in-Aid for Scientific Research on Innovative Areas of MEXT (Grant number: JP17H06414); and Takeda Science Foundation. This work was supported by grants from Sugiura Dermatology Clinic, Otsuka Pharmaceutical.

## Declarations of interest

The authors declare no conflicts of interest associated with this manuscript.

## Data Availability

Data will be made available on request.

## Footnotes

Appendix A Supplementary data associated with this article can be found online at doi:10.1016/j.cstres.2025.02.001.
