# Supplementary material for: Increased intracellular stress responses and decreased KLF2 in adult patients with atopic dermatitis
Source: Cell Stress Chaperones. 2025 Feb 10;30(2):84–99. doi: 10.1016/j.cstres.2025.02.001 (PMC11891603; doi:10.1016/j.cstres.2025.02.001)
Supplement: Supplementary file 1 — Supplementary material [file mmc1.pdf]

Supplementary material

Table of contents

Abbreviations……………………………………………………….…..……………..4

Results

Table S1. List and Function of Novel Candidate Genes Found by Microarray Analysis, Including Normal-appearing Skin in Patients with Atopic Dermatitis That May Contribute to Pathogenesis…..……………………………………………5

Figure S1. Fluctuation in Expression in An Individual Case of All 92 Genes Identified by The Clustering Analysis…………………………………………………18

Microarray Analysis………………………………..…………………………..…30

Table S2. Outline of the Intensity of Immunohistochemistry (IHC)….………………..31

IHC Analysis……………………………………………………………………34

Figure S2. Immunofluorescence study for CD3/KLF2 double staining………………..35

Discussion on Genes Thought To Play An Important Role in The Pathogenesis of AD, But Not Introduced in The Main Text...……………………………….37

Lipid Metabolism…………………………………………………………….…...38

Hypertension is Rare in Adult Patients with AD……………………..……….……..40

Possible Cause of Th1/Th2 Imbalance……………………………………..………..41

Genes with Increased Expression Only in Non-Lesional Areas, Particularly the Function of PIP…………………………..…………………………...………...42

MHC I Enhancement and Autoimmunity in AD………………………………………44

Amyloid Deposition…………………………………………………………….…...45

Close Interaction and Gene Locus Between NAB1 and EGR1………………………..46

Does Change in the Expression of Chaperones, B2M, KLF2, BGN, and LEPR Occur in Organs Other Than the Skin?...47

WIF1 and Keratinocyte Proliferation…………………………………………………..48

Exacerbation and Spreading of Eczema by DAMPS…………………………………..49

Special Remarks………………………………………………………………………..50

Previous reports on HSP expression in AD skin lesions and healthy skin……………..51

Methods

Biopsy…………………………………………………………………………………52

Table S3. Backgrounds of All Patients with AD Examined By Both Microarray Analysis and Immunohistochemical (IHC) Analysis…………………………...……………..53

Figure S3. Representative Clinical Features of Patients with AD……………………..55

RNA Preparation, DNA Microarray, and Statistical Analysis………………………….56

Immunohistochemistry (IHC) …………………………………..………………...58

Immunofluorescence Analysis for CD4/CD8 Double Staining……………...…………59

Table S3. The Probe Set of Microarray Analysis of 92 Genes with Significant Differences in Expression……………………………………………………..60

References…………………………………………………………………...64

ABBREVIATIONS

CYP1B1, cytochrome P450 family 1 subfamily B member 1

FADS1, fatty acid desaturase 1

FLG, filaggrin

LOR, loricrin cornified envelope precursor protein

MED16, mediator complex subunit 16

MSMO1, methylsterol monooxygenase 1

MUC1, mucin 1, cell surface associated

NAB2, NGFI-A binding protein 2

PCSK5, proprotein convertase subtilisin/kexin type 5

PURA, purine rich element binding protein A

SCGB1D2, secretoglobin family 1D member 2

Table S1.

List and function of novel candidate genes found by microarray analysis including normal-appearing skin in patients with atopic dermatitis that may contribute to pathogenesis

Official Symbol	location	Gene name	Function	References
		**Up regulated in both uninvolved and involved skin in AD patients (group 3)**		
**SP110**	2q37.1	SP110 nuclear body protein	nuclear body proteins and encodes a leukocyte-specific nuclear body component. The protein can function as an activator of gene transcription and may serve as **a nuclear hormone receptor coactivator**.<br>the protein may play a role in ribosome biogenesis and in the induction	Sp110 localizes to the PML-Sp100 nuclear body and may function as a nuclear hormone receptor transcriptional coactivator. Bloch DB, Nakajima A, Gulick T, et al. Mol Cell Biol. 2000 Aug;20(16):6138-46.
**VADC1**	Xq21.31 Yp11.2	voltage-dependent anion channel 1	The voltage-dependent anion channel 1 (VDAC1) protein, is an important regulator of mitochondrial function, and serves as a mitochondrial gatekeeper, with responsibility for cellular fate.	VDAC1 at the Intersection of Cell Metabolism, Apoptosis, and Diseases. Shoshan-Barmatz V, Shteinfer-Kuzmine A, Verma A, Biomolecules. 2020 Oct 26;10(11):1485.
**APLP2**	11q24.3	**Amyloid beta (A4) precursor-like protein 2**	APLP2 is active in several physiological processes, including cell adhesion, migration, cell signaling, and cell cycle regulation.<br>APLP2 binds to **MHC class I** molecules in human tumor cells, and that increased APLP2 expression causes **down-regulation of human MHC class I** molecules at the plasma membrane.	Amyloid precursor-like protein 2 association with HLA class I molecules. Tuli A, Sharma M, Wang X, et al. Cancer Immunol Immunother. 2009 Sep;58(9):1419-31.
**B2M**	15q21.1	**Beta-2-microglobulin**	found in association with the **major histocompatibility complex (MHC) class I heavy chain** on the surface of nearly all nucleated cells.<br>The protein has B-sheets. Increased serum levels and misfolding have been linked to a pathological condition known as dialysis - related **amyloidosis** (DRA).	Excess beta 2 microglobulin promoting functional peptide association with purified soluble class I MHC molecules. Kozlowski S, Takeshita T, Boehncke WH, et al. Nature. 1991 Jan 3;349(6304):74-7.<br>Molecular insights into cell toxicity of a novel familial amyloidogenic variant of β2-microglobulin. Leri M, Bemporad F, Oropesa-Nuñez R, et al. J Cell Mol Med. 2016 Aug;20(8):1443-56.
**HSPA9**	5q31.2	**heat shock 70kDa protein 9 (mortalin)**	A heat-uninducible member of **chaperones**. An immortalization marker. Mortalin regulates p53 and plays important roles in stress response and maintenance of the mitochondria and endoplasmic reticulum, cell proliferation, apoptosis, vesicular transport of proteins and neurodegenerative diseases.	Mortalin - a multipotent chaperone regulating cellular processes ranging from viral infection to neurodegeneration. Flachbartová Z, Kovacech B. Acta Virol. 2013;57(1):3-15. Review.
EIF5B	2p16.1	eukaryotic translation initiation factor 5B	Mitochondrial Translational Initiation Factor 2: One of the essential components for the initiation of protein synthesis.	Structural insights on the translation initiation complex: ghosts of a universal initiation complex. Allen GS, Frank J. Mol Microbiol. 2007 Feb;63(4):941-50. Review.

PTP4A2	1p35.2	Protein tyrosine phosphatase type IVA member 2	DUSPs (dual-specificity phosphatases): protein phosphatases that can dephosphorylate both phosphotyrosine and phosphoserine/phosphothreonine residues within the one substrate. PTP4A2 stimulates progression from G1 into S phase during mitosis. Promotes tumors.	Enhanced cell cycle progression and down regulation of p21(Cip1/Waf1) by PRL tyrosine phosphatases. Werner SR, Lee PA, DeCamp MW, Crowell DN, Randall SK, Crowell PL. Cancer Lett. 2003 Dec 30;202(2):201-11.<br>Dual-specificity phosphatases: critical regulators with diverse cellular targets. Patterson KI, Brummer T, O'Brien PM, Daly RJ. Biochem J. 2009 Mar 15;418(3):475-89.
HNRNPC	14q11.2	Heterogeneous nuclear ribonucleoprotein C (C1/C2)	Heterogeneous nuclear protein. influence pre-mRNA splicing. An interaction of hnRNPs C1 and C2 with hnRNP A1 and telomerase may be important for telomerase to function on the telomere.	Heterogeneous nuclear ribonucleoproteins C1 and C2 associate with the RNA component of human telomerase. Ford LP, Suh JM, Wright WE, Shay JW. Mol Cell Biol. 2000 Dec;20(23):9084-91.
DDX1	2p24.3	DEAD (Asp-Glu-Ala-Asp) box helicase 1	ATP-dependent RNA helicases that function in multiple steps of RNA metabolism. DEAD box proteins play a role in antiviral immune response. DDX1 is involved in the sensing of dsRNA and production of an interferon response.	DExD/H-box RNA helicases as mediators of anti-viral innate immunity and essential host factors for viral replication. Fullam A, Schröder M. Biochim Biophys Acta. 2013 Aug;1829(8):854-65
**NAB1**	2q32.2	**NGFI-A binding protein 1（EGR1 binding protein 1）**	NAB2 can inhibit EGR1, EGR2, and EGR3, whereas **NAB1 is able to inhibit the activity of only EGR1 and EGR2**. Furthermore, NAB1 is constitutively expressed in most cell types, while NAB2 is a delayed early response gene and is induced by the same environmental signals that lead to EGR expression. EGR1 has been implicated in cell proliferation, macrophage differentiation, synaptic activation, and long-term potentiation, whereas **EGR2 is critical for proper hindbrain segmentation and peripheral nerve myelination**.	EGR1, EGR2, and EGR3 activate the expression of their coregulator NAB2 establishing a negative feedback loop in cells of neuroectodermal and epithelial origin. Kumbrink J, Kirsch KH, Johnson JP. J Cell Biochem. 2010 Sep 1;111(1):207-17.<br>Identification of NAB1, a repressor of NGFI-A- and Krox20-mediated transcription. Russo MW, Sevetson BR, Milbrandt J. Proc Natl Acad Sci U S A. 1995 Jul 18;92(15):6873-7.

List and function of novel candidate genes found by microarray analysis

including normal-appearing skin in patients with atopic dermatitis that may contribute to pathogenesis

**HSP90B 1**	12q23.3	**heat shock protein 90kDa beta (Grp94), member 1**	Hsp90b1 is an **endoplasmic reticulum master chaperone** for multiple Toll-like receptors (TLRs) in mice. Hsp90b1 is essenntial for transition from pro-B to pre-B cell. Hsp90b1 is the only known molecular chaperone to specifically regulate T- and B-cell development. Hsp90b1 helps **thymocytes** to develop beyond the **CD4**(-)**CD8**(-) stage.<br>**MHC class I** antigen presentation pathway.	gp96, an endoplasmic reticulum master chaperone for integrins and Toll-like receptors, selectively regulates early T and B lymphopoiesis. Staron M, Yang Y, Liu B, Li J, Shen Y, Zúñiga-Pflücker JC, Aguila HL, Goldschneider I, Li Z. Blood. 2010 Mar 25;115(12):2380-90.<br>Heat shock protein gp96 is a master chaperone for toll-like receptors and is important in the innate function of macrophages. Yang Y, Liu B, Dai J, et al. Immunity. 2007 Feb;26(2):215-26. Epub 2007 Feb 1.<br>Regulatory role of microRNA in mesenteric lymph nodes after Salmonella Typhimurium infection. Herrera-Uribe J, Zaldívar-López S, Aguilar C, et al. Vet Res. 2018 Feb 1;49(1):9. doi: 10.1186/s13567-018-0506-1.
**CANX**	5q35.3	**Calnexin**	**ER chaperone**. Regulates the folding of **MHC class I** molecules. Immature **thymocytes** express clonotype-independent CD3 complexes that, when engaged by anti-CD3 antibodies, can signal **CD4**(-)**CD8**(-) **thymocytes** to differentiate into **CD4**(+)**CD8**(+) cells.	The quality control of MHC class I peptide loading. Wearsch PA, Cresswell P. Curr Opin Cell Biol. 2008 Dec;20(6):624-31,<br>The molecular chaperone calnexin is expressed on the surface of immature thymocytes in association with clonotype-independent CD3 complexes. Wiest DL, Burgess WH, McKean D, Kearse KP, Singer A. EMBO J. 1995 Jul 17;14(14):3425-33.
**RNF11**	1p32.2	**Ring finger protein 11**	RNF11 blocks type I IFN production. RNF11 is a key negative regulator of NF-κB signaling in **proinflammatory pathways**.<br>RNF11 inhibits antiviral signaling and virus-induced IFN-β production.	RING finger protein 11 targets TBK1/IKKi kinases to inhibit antiviral signaling. Charoenthongtrakul S, Gao L, Parvatiyar K, Lee D, Harhaj EW. PLoS One. 2013;8(1):e53717.
**MLEC**	12q24.31	**malectin**	**ER chaperone**. Binding to G2M9 glycans. malectin might induce the **ER-associated degradation (ERAD)**. Capturing misfolded glycoproteins and attenuating the cytotoxic effects of their accumulation.	Role of malectin in Glc(2)Man(9)GlcNAc(2)-dependent quality control of α1-antitrypsin. Chen Y, Hu D, Yabe R, et al. Mol Biol Cell. 2011 Oct;22(19):3559-70
		**Down regulated in both uninvolved and involved skin in AD patients (group 2)**		
**CDHR1**	10q23.1	**cadherin-related family member 1**	Homophilic cell-adhesion proteins. Protocadherins are predominantly expressed in the **nervous system**, CDHR1 is a photoreceptor-specific cadherin. Multiple protocadherins across several subfamilies have been implicated as modulators of **Wnt signaling pathways**, and through this as potential tumor suppressors.	CDHR1 mutations in retinal dystrophies. Stingl K, Mayer AK, Llavona P, et al. Sci Rep. 2017 Aug 1;7(1):6992.<br>Regulation of Wnt signaling by protocadherins. Mah KM, Weiner JA. Semin Cell Dev Biol. 2017 Sep;69:158-171.

**KLF2**	19p13.11	**Kruppel-like factor2 (lung)**	Zinc finger protein. Transcription of the KLF2 gene is induced during the differentiation of **immature double-positive (CD4+CD8+) to mature single-positive (CD4+ or CD8+) thymocytes** and KLF2 expression remains high in quiescent circulating T cells. Following activation through the T-cell antigen receptor, the transcription of the KLF2 gene is extinguished and pre-existing KLF2 protein is degraded.<br>**Suppressing the expression of KLF2 in T cells and monocytes leads to a pro-inflammatory phenotype of these cell types.**<br>KLF2 is an antiadipogenic factor which binds to and represses the **Peroxisome proliferator-activated receptor(PPAR)γ** promoter. PPAR γ activates the expression of **lipid metabolizing enzymes.**<br>**UVA induce KLF2 up-regulation**.<br>KLF2 has been identified as an inhibitor of NF-κB mediated inflammatory activities and hence has been of research interest in inflammation and mediated chronic inflammatory diseases. KLF2 has been identified as a potent inhibitor of NF-κB dependent HIF-1α signaling and is a critical determinant of myeloid cell activation and host response in polymicrobial infection and endoxemia.	The LKLF transcription factor is required for normal tunica media formation and blood vessel stabilization during murine embryogenesis. Kuo CT, Veselits ML, Barton KP, Lu MM, Clendenin C, Leiden JM. Genes Dev. 1997 Nov 15;11(22):2996-3006. Statin-induced Krüppel-like factor 2 expression in human and mouse T cells reduces inflammatory and pathogenic responses. Bu DX, Tarrio M, Grabie N, et al. J Clin Invest. 2010 Jun; 120(6):1961-70.<br>Kruppel-like factor 2 (KLF2) regulates proinflammatory activation of monocytes. Das H, Kumar A, Lin Z, et al. Proc Natl Acad Sci U S A. 2006 Apr 25; 103(17):6653-8.<br>Platycodin D inhibits adipogenesis of 3T3-L1 cells by modulating Kruppel-like factor 2 and peroxisome proliferator-activated receptor gamma. Lee H, Kang R, Kim YS, Chung SI, Yoon Y. Phytother Res. 2010 Jun;24 Suppl 2:S161-7.<br>Ultraviolet A regulates adipogenic differentiation of human adipose tissue-derived mesenchymal stem cells via up-regulation of Kruppel-like factor 2. Lee J, Lee J, Jung E, et al. J Biol Chem. 2010 Oct 15;285(42):32647-56.<br>KLF2 in Regulation of NF-κB-Mediated Immune Cell Function and Inflammation. Jha P, Das H. Int J Mol Sci. 2017 Nov 10;18(11). pii: E2383. doi: 10.3390/ijms18112383.
TNNI2	11p15.5	troponin I type 2 (skeletal, fast)	subunit of the troponin complex in striated muscle (skeletal and cardiac muscles). TNNI2 is encoding the fast skeletal muscle isoform of TnI (fsTnI). Troponin I interacts with all known regulatory proteins in the thin filament: TnC, TnT, actin, and tropomyosin, reflecting its key position in the Ca2+ regulation of striated muscle contraction.	TNNI1, TNNI2 and TNNI3: Evolution, regulation, and protein structure-function relationships. Sheng JJ, Jin JP. Gene. 2016 Jan 15;576(1 Pt 3):385-94.

List and function of novel candidate genes found by microarray analysis

including normal-appearing skin in patients with atopic dermatitis that may contribute to pathogenesis

**EGR1**	5q31.2	**Early growth response 1**	zinc-finger protein. **EGR1 expression is low in resting cells**, it is rapidly and transiently induced by a wide variety of environmental signals including growth factors, cytokines, and toxic substances. EGR1 induce of cellular programs of differentiation, growth, and cell death through changes in the expression of EGR1 target genes.	Egr-1 induces the expression of its corepressor nab2 by activation of the nab2 promoter thereby establishing a negative feedback loop. Kumbrink J, Gerlinger M, Johnson JP. J Biol Chem. 2005 Dec 30;280(52):42785-93. Epub 2005 Oct 31.
LOC1002	5q13.2	LOC340111	uncharacterized LOC100272216	

**BGN**	Xq28	**Biglycan**	The encoded preproprotein is proteolytically processed to generate the mature protein, which plays a role in **bone growth, muscle development and regeneration, and collagen fibril assembly** in multiple tissues.<br>This protein may also regulate inflammation and innate immunity. Biglycan is a trigger of **Th1 and Th17** cell recruitment into the kidney.	Targeted disruption of the biglycan gene leads to an osteoporosis-like phenotype in mice. Xu T, Bianco P, Fisher LW, et al. Nat Genet. 1998 Sep;20(1):78-82.<br>Phenotypic effects of biglycan deficiency are linked to collagen fibril abnormalities, are synergized by decorin deficiency, and mimic Ehlers–Danlos-like changes in bone and other connective tissues. Corsi A, Xu T, Chen XD, et al.J Bone Miner Res. 2002 Jul;17(7):1180-9.<br>Excess biglycan causes eyelid malformation by perturbing muscle development and TGF-alpha signaling. Hayashi Y, Liu C-Y, Jester JJ, et al. (2005) Dev Biol. 2005 Jan 1;277(1):222-34.<br>Defective glycosylation of decorin and biglycan, altered collagen structure, and abnormal phenotype of the skin fibroblasts of an Ehlers–Danlos syndrome patient carrying the novel Arg270Cys substitution in galactosyltransferase I (β4GalT-7), Seidler D, Faiyaz-Ul-Haque M, Hansen U, et al. J Mol Med (Berl). 2006 Jul;84(7):583-94. Epub 2006 Apr 1.<br>Interaction of biglycan with type I collagen. Schönherr E, Witsch-Prehm P, Harrach B, Robenek H, Rauterberg J, Kresse H. J Biol Chem. 1995 Feb 10;270(6):2776-83.<br>The matrix component biglycan is proinflammatory and signals through Toll-like receptors 4 and 2 in macrophages. Schaefer L, Babelova A, Kiss E, et al. J Clin Invest. 2005 Aug;115(8):2223-33. Epub 2005 Jul 14.<br>Biglycan, a novel trigger of Th1 and Th17 cell recruitment into the kidney. Nastase MV, Zeng-Brouwers J, Beckmann J, et al. Matrix Biol. 2018 Aug;68-69:293-317.

List and function of novel candidate genes found by microarray analysis
including normal-appearing skin in patients with atopic dermatitis that may contribute to pathogenesis

**WIF1**	12q14.3	**WNT inhibitory factor 1**	WIF1 is a noncanonical **Wnt inhibitor**, and **a marker for keratinocyte stem cells (KSCs)**. Wnt signaling pathway inhibitors expressed by KSCs were Sfrp-1, Dkk-3, and WIF1. WIF1 was the single most overexpressed gene in KSCs.<br>WIF1 is highly expressed in non-cycling cells, indicating a role in **negatively regulating keratinocyte proliferation**.<br>Wnt signaling is also involved in self-renewal and maintenance of hematopoietic stem cells (HSCs). Wif1 in osteoblasts led to a reduction of quiescent HSCs but did not alter bone architecture.	WIF1 is expressed by stem cells of the human interfollicular epidermis and acts to suppress keratinocyte proliferation. Schlüter H, Stark HJ, Sinha D, Boukamp P, Kaur P. J Invest Dermatol. 2013 Jun;133(6):1669-73.<br>Noncanonical Wnt signaling maintains hematopoietic stem cells in the niche. Sugimura R, He XC, Venkatraman A, et al. Cell. 2012 Jul 20;150(2):351-65
ZNF273	7q11.21	Zinc finger protein 273	not enough disclosed.<br>a member of the krueppel C2H2-type zinc-finger protein family and encodes a protein with 13 C2H2-type zinc fingers and a KRAB domain.	none
**FN1**	2q35	**Fibronectin 1**	Fibronectin (FN1) mediates a wide variety of cellular interactions with the extracellular matrix (ECM) and plays important roles in **cell adhesion**, migration, growth and differentiation.<br>The FN1 molecule contains a large repertoire of binding sites for cell adhesion molecules, other ECM components, and cell signaling molecules: integrins, vascular endothelial growth factor-A (VEGF), fibrillin, latent TGF-β binding protein (LTBP).<br>FN1 also mediates a particular viral infection.	Fibronectin at a glance. Pankov R, Yamada KM. J Cell Sci. 2002 Oct 15;115(Pt 20):3861-3. The extracellular matrix: not just pretty fibrils. Hynes RO. Science. 2009 Nov 27;326(5957):1216-9.

List and function of novel candidate genes found by microarray analysis
including normal-appearing skin in patients with atopic dermatitis that may contribute to pathogenesis

**ID4**	6p22.3	**Inhibitor of DNA binding 4, dominant negative helix-loop-helix protein**	ID proteins (ID1, ID2, ID3 and ID4) are dominant negative transcriptional regulators of basic Helix Loop Helix (bHLH) transcription factors that lack the basic DNA binding domain but have intact HLH domain. ID4 can act as pro- or anti-differentiation factor in a cell specific manner. In general, ID4 expression is down-regulated in ALL. Some studies suggest that **ID4 supports angiogenesis** in cancers, ID4 exists in positive feedback loop with androgen receptor(AR) but in a negative feedback loop with Estrogen Receptor(ER). Ectopic expression of **ID4 blocks cell cycle** at S-Phase and **inhibits proliferation** in prostate cancer cell line DU145. Loss of ID4 also promotes progression into S-phase in neuronal early cortical progenitor cells.<br>IDs are direct downstream targets of the transforming growth factor (TGF)-β pathway. TGF-β inhibits IDs.	Inhibitor of differentiation 4 (ID4): From development to cancer. Patel D, Morton DJ, Carey J, Havrda MC, Chaudhary J. Biochim Biophys Acta. 2015 Jan;1855(1):92-103.<br>Id2, Id3 and Id4 overcome a Smad7-mediated block in tumorigenesis, generating TGF-β-independent melanoma. DiVito KA, Simbulan-Rosenthal CM, Chen YS, Trabosh VA, Rosenthal DS. Carcinogenesis. 2014 Apr;35(4):951-8
**LOR**	1q21.3	**Loricrin**	Loricrin and involucrin are **major components of the cornified envelope**. The small proline-rich proteins (SPRRs) show strong sequence homology to loricrin and involucrin proteins. Microarray analysis of atopic skin lesions revealed altered expression of genes located within the epidermal differentiation complex (EDC), in particular upregulation of S100A8 and S100A7 and downregulation of loricrin and filaggrin, compared with healthy control samples.<br>*SPRRs: In addition to their function as structural proteins it has been suggested that they might also be involved in gene regulation, possibly in limiting proliferation and promoting differentiation.	On the role of the epidermal differentiation complex in ichthyosis vulgaris, atopic dermatitis and psoriasis. Hoffjan S, Stemmler S. Br J Dermatol. 2007 Sep;157(3):441-9. Epub 2007 Jun 15.<br>Large-scale DNA microarray analysis of atopic skin lesions shows overexpression of an epidermal differentiation gene cluster in the alternative pathway and lack of protective gene expression in the cornified envelope. Sugiura H, Ebise H, Tazawa T, et al. Br J Dermatol. 2005 Jan;152(1):146-9.

**LEPR**	1q31.3	**Leptin receptor**	In innate immunity, leptin enhances the activity and function of neutrophils, and **shifting of T-cells toward the Th1 phenotype**. In adaptive immunity, leptin induces the **maturation and survival of thymic T-cells**. Leptin deficiency results in severe immune defects characterized by decrease in total lymphocytes, CD4+ helper T cell number, increased **thymocyte** apoptosis, and a **skewing away from the Th1 toward Th2** phenotype. Lack or inhibition of leptin/leptin receptor pathway protects against the development of various immunoinflammatory diseases. Protection was associated with a shift of the cytokine profile toward **increased Th2/Treg type**, Foxp3 expresstion, number of Treg cells in lymphoid organs of mice with defective leptin signaling. **Thymocytes** treated with leptin induces **CD4(+)CD8(+) cell differentiation mainly to CD4+ mature thymocytes**. **Unlike effector CD4(+) and CD8(+) T cells, Tregs and memory T cells oxidize** **fatty acids** **for fuel**. Upon activation, T cells express the insulin and leptin receptors and become sensitive to insulin signaling and nutrient availability and show changes in differentiation.	Defective leptin/leptin receptor signaling improves regulatory T cell immune response and protects mice from atherosclerosis. Taleb S, Herbin O, Ait-Oufella H, et al. Arterioscler Thromb Vasc Biol. 2007 Dec;27(12):2691-8. Epub 2007 Aug 9.<br>Metabolism of activated T lymphocytes. Maciolek JA, Pasternak JA, Wilson HL. Curr Opin Immunol. 2014 Apr;27:60-74.<br>Leptin Functions in Infectious Diseases. Maurya R, Bhattacharya P, Dey R, Nakhasi HL. Front Immunol. 2018 Nov 26;9:2741

**PCSK5**	9q21.13	**Proprotein convertase subtilisin/kexin type 5**	**Proprotein convertase subtilisin/kexin 5 (PCSK5) cleaves prohormones at consensus sequences, present from the endoplasmic reticulum (ER) to the trans Golgi network (TGN) or at the cell surface**. PCSK5, PCSK7 and Furin are present in human **CD4(+) T cells** with different expression patterns. GDF11 is a TGFβ superfamily molecule and is cleaved and activated by PCSK5. Some of convertases play critical roles in the **regulation of lipids and/or sterols through the inactivation of lipases**, e.g. by PCSK5, PACE4, and furin; through the activation of specific membrane-bound transcription factors (SREBP-1 and SREBP-2) by SKI-1/S1P. PCSK5 regulates the level of mature PCSK9. PCSK5 directly **inactivate endothelial lipase**. **HDL particles are modulated by a variety of lipases, including vascular endothelial lipase**.	C-terminal cleavage of human Foxp3 at a proprotein convertase motif abrogates its suppressive function. Elhage R, Cheraï M, Levacher B, et al. Scand J Immunol. 2015 Apr;81(4):229-39.<br>Processing specificity and biosynthesis of the Drosophila melanogaster convertases dfurin1, dfurin1-CRR, dfurin1-X, and dfurin2. De Bie I, Savaria D, Roebroek AJ, et al. J Biol Chem. 1995 Jan 20;270(3):1020-8.<br>PCSK5 and GDF11 expression in the hindgut region of mouse embryos with anorectal malformations. Tsuda T, Iwai N, Deguchi E, et al. Eur J Pediatr Surg. 2011 Aug;21(4):238-41.<br>PCSK5 mutation in a patient with the VACTERL association. Nakamura Y, Kikugawa S, Seki S, et al. BMC Res Notes. 2015 Jun 9;8:228.<br>The proprotein convertase (PC) PCSK9 is inactivated by furin and/or PC5/6A: functional consequences of natural mutations and post-translational modifications. Benjannet S, Rhainds D, Hamelin J, Nassoury N, Seidah NG. J Biol Chem. 2006 Oct 13;281(41):30561-72. Epub 2006 Aug 15.<br>Genetic variation at the proprotein convertase subtilisin/kexin type 5 gene modulates high-density lipoprotein cholesterol levels. Iatan I, Dastani Z, Do R, et al. Circ Cardiovasc Genet. 2009 Oct;2(5):467-75.
FHL1	Xq26.3	Four and a half LIM domains 1	FHL1 contains the zinc finger domain. FHL1 is a protein harboring domains capable of binding both the transcriptional machinery and the actin cytoskeleton. FHL1 was selectively and significantly upregulated in settings of pathological cardiac hypertrophy, signaling, and disease. FHL1, a transcriptional regulator associated with Emery–Dreifuss muscular dystrophy, has been shown to play a key role in nuclear anchorage and myotube hypertrophy. Pathogenetic mutations in FHL1 cause massive protein mislocalization and accumulation into perinuclear aggregates called aggresomes. FHL1 regulates cell morphological change.	Four and a half LIM domain protein signaling and cardiomyopathy. Liang Y, Bradford WH, Zhang J, Sheikh F. Biophys Rev. 2018 Jun 20.<br>Laminopathies and lamin-associated signaling pathways. Maraldi NM, Capanni C, Cenni V, Fini M, Lattanzi G. J Cell Biochem. 2011 Apr;112(4):979-92<br>Four-and-a-half LIM Domains 1 (FHL1) Protein Interacts with the Rho Guanine Nucleotide Exchange Factor PLEKHG2/FLJ00018 and Regulates Cell Morphogenesis. Sato K, Kimura M, Sugiyama K, et al. J Biol Chem. 2016 Nov 25;291(48):25227-25238. Epub 2016 Oct 20.

List and function of novel candidate genes found by microarray analysis
including normal-appearing skin in patients with atopic dermatitis that may contribute to pathogenesis

**FLG**	1q21.3	**Filaggrin**	The name filaggrin is a contraction of 'filament aggregating protein'. Filaggrin is a key protein that facilitates terminal differentiation of the epidermis and formation of the **skin barrier**. Filaggrin is the major genetic risk factor for AD and atopy.	Filaggrin failure - from ichthyosis vulgaris to atopic eczema and beyond. McLean WH. Br J Dermatol. 2016 Oct;175 Suppl 2:4-7.<br>Common loss-of-function variants of the epidermal barrier protein filaggrin are a major predisposing factor for atopic dermatitis. Palmer CN, Irvine AD, Terron-Kwiatkowski A, et al. Nat Genet. 2006 Apr;38(4):441-6. Epub 2006 Mar 19.
**CYP1B1**	2p22.2	**Cytochrome p450, family 1, subfamily B, polypeptide 1**	CYP1B1 was shown to be important in regulating endogenous metabolic pathways, **including the metabolism of steroid hormones, fatty acids**, melatonin, and vitamins. CYP1B1 and nuclear receptors including **peroxisome proliferator-activated receptors (PPARs)**, estrogen receptor (ER), and retinoic acid receptors (RAR) contribute to the maintenance of the homeostasis of these endogenous compounds. Modulation of CYP1B1 can **decrease adipogenesis** and tumorigenesis, and cancer. **CYP1B1 inhibitors are effective in the treatment of hypertension**, obesity, and atherosclerosis.	Potential role of CYP1B1 in the development and treatment of metabolic diseases. Li F, Zhu W, Gonzalez FJ. Pharmacol Ther. 2017 Oct;178:18-30.
**PURA**	5q31.3	**Purine-rich element binding protein A**	PURA is an evolutionarily conserved cellular protein participating in processes of DNA replication, transcription, and RNA transport; all involving binding to nucleic acids and altering conformation and physical positioning. PURA provokes **neurodevelopmental disorders**.	PURA-Related Neurodevelopmental Disorders. Reijnders MRF, Leventer RJ, Lee BH, et al. GeneReviews® [Internet]. Seattle (WA): University of Washington, Seattle; 1993-2018. 2017 Apr 27.<br>Regulation of PURA gene transcription by three promoters generating distinctly spliced 5-prime leaders: a novel means of fine control over tissue specificity and viral signals. Wortman MJ, Hanson LK, Martínez-Sobrido L, et al. BMC Mol Biol. 2010 Nov 9;11:81.
		**Up regulated in only uninvolved skin in AD patients (group 1)**		
**RAPGEF3**	12q13.11	**Rap guanine nucleotide exchange**	Potently increases **endothelial barrier function**. Required for the actin rearrangement at cell-cell junctions.	Rap1 signaling in endothelial barrier control. Pannekoek WJ, Post A, Bos JL. Cell Adh Migr. 2014;8(2):100-7.
MYH11	16q13.11	Myosin heavy polypeptide 11, smooth muscle	smooth muscle myosin belonging to the myosin heavy chain family. Not enough disclosed.	

ATP1B1	1q24.2	ATPase Na/ka transporting beta 1 polypeptide	not enough disclosed.	
**MSMO1**	4q32.3	**methylsterol monooxygenase 1**	localized to the **endoplasmic reticulum** membrane and is believed to function in cholesterol biosynthesis. Psoriasiform Dermatitis.	Cholesterol biosynthesis pathway as a novel mechanism of resistance to estrogen deprivation in estrogen receptor-positive breast cancer. Simigdala N, Gao Q, Pancholi S, et al. Breast Cancer Res. 2016 Jun 1;18(1):58,<br>A rare case of sterol-C4-methyl oxidase deficiency in a young Italian male: Biochemical and molecular characterization. Frisso G, Gelzo M, Procopio E, et al. Mol Genet Metab. 2017 Aug;121(4):329-335
**PIP**	7q34	**Prolactin-induced protein**	Prolactin-induced protein (PIP) has been shown to **bind to CD4 and is speculated to block CD4-HLA-DR interaction**. PIP might have a local and systemic **immunosuppressive effect** in mouse chronic allergic contact dermatitis (ACD).<br>Aspartic peptidase of PIP found in sweat or saliva deteriorates the skin barrier in a de novo manner, which potentially **leads directly to the proliferation** of epidermal keratinocytes without any external antigenic factors.	Immunosuppressive effect of prolactin-induced protein: a new insight into its local and systemic role in chronic allergic contact dermatitis. Sugiura S, Fujimiya M, Ebise H, et al. Br J Dermatol. 2010 Jun;162(6):1286-93.<br>Effect of prolactin-induced protein on human skin: new insight into the digestive action of this aspartic peptidase on the stratum corneum and its induction of keratinocyte proliferation. Sugiura S, Tazuke M, Ueno S, et al. J Invest Dermatol. 2015 Mar;135(3):776-785.
**FADS1**	11q12.2	**Fatty acid desaturase 1**	member of the **fatty acid desaturase (FADS)** gene family.<br>fatty acids(PUFAs), FADS1, FADS2 can influence inflammatory processes.<br>Subjects carrying the minor alleles of several single nucleotide polymorphisms of the FADS1, FADS2 had a lower prevalence of allergic rhinitis and atopic eczema.	Polyunsaturated fatty acids, inflammatory processes and inflammatory bowel diseases. Calder P.C. Mol Nutr Food Res, 52 (2008), pp. 885-897<br>Common genetic variants of the FADS1 FADS2 gene cluster and their reconstructed haplotypes are associated with the fatty acid composition in phospholipids. Schaeffer L., Gohlke H., Muller M., et al. Hum Mol Genet, 15 (2006), pp. 1745-1756
MUC1	4q32.3	mucin 1, cell surface associated	O-glycosylated proteins that play mucous barriers on epithelial surfaces against bacterial and enzyme attack. intracellular signaling in ERK, SRC and NF-kappa-B pathways	

List and function of novel candidate genes found by microarray analysis
including normal-appearing skin in patients with atopic dermatitis that may contribute to pathogenesis

SCGB1D2	11q12.3	Secretoglobin family 1D member 2	They are found in several other tissues, notably glands and steroid-rich organs.	Expression analysis of mammaglobin A (SCGB2A2) and lipophilin B (SCGB1D2) in more than 300 human tumors and matching normal tissues reveals their co-expression in gynecologic malignancies. Zafrakas M, Petschke B, Donner A, et al. BMC Cancer. 2006 Apr 9;6:88.
**MED16**	19p13.3	**mediator complex subunit 16**	**thyroid hormone receptor**-associated proteins(TRAPs). **thyroid hormone receptor** (TR)/TRAP complex markedly activates transcription.	Ligand induction of a transcriptionally active thyroid hormone receptor coactivator complex. Fondell JD, Ge H, Roeder RG. Proc Natl Acad Sci U S A. 1996 Aug 6;93(16):8329-33.
IGFBP5	2q35	Insulin-like growth factor binding protein 5	activates transcription	Insulin-like growth factor binding protein 5 induces skin fibrosis: A novel murine model for dermal fibrosis. Yasuoka H, Jukic DM, Zhou Z, Choi AM, Feghali-Bostwick CA.Arthritis Rheum. 2006 Sep;54(9):3001-10.

Figure S1.

Up regulated in uninvolved and subacute skin in AD patients

MYH6 /// MYH7

myosin, heavy chain 6, cardiac muscle, alpha /// myosin, heavy chain 7, cardiac muscle, beta

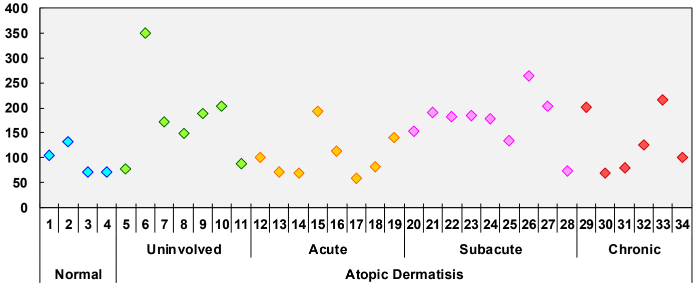

SOS2

SOS Ras/Rho guanine nucleotide exchange factor 2

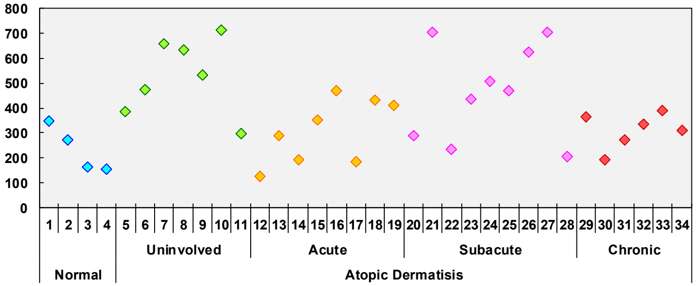

ITGB1BP1

integrin beta 1 binding protein 1

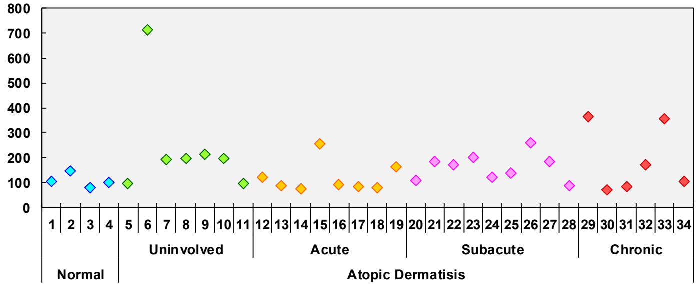

RPL37A

ribosomal protein L37a

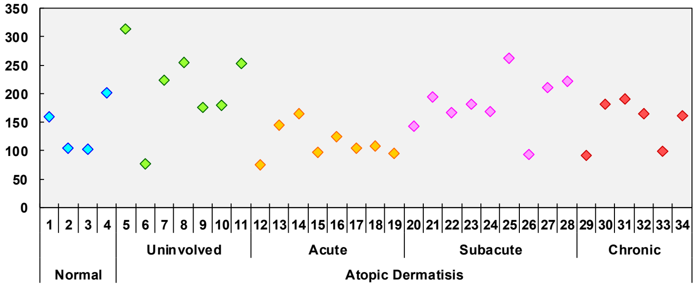

EPB41L1

erythrocyte membrane protein band 4.1-like 1

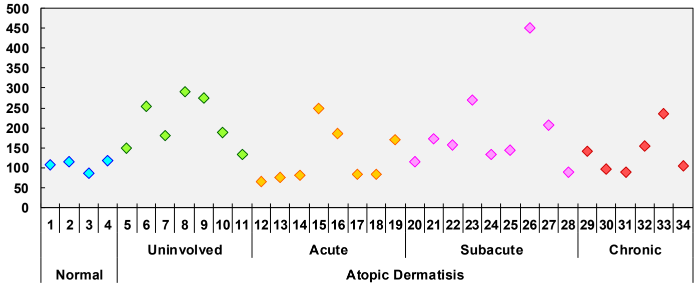

HSPD1

heat shock 60kDa protein 1 (chaperonin)

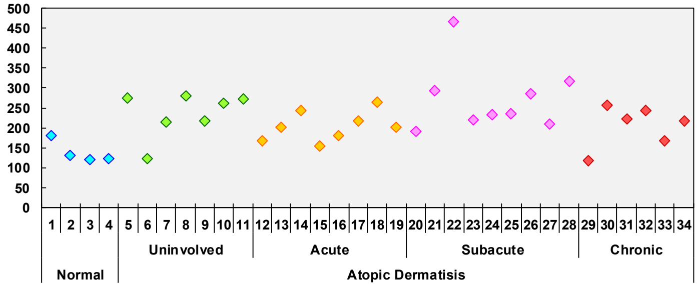

NDUFS2

NADH dehydrogenase (ubiquinone) Fe-S protein 2, 49kDa (NADH-coenzyme Q reductase)

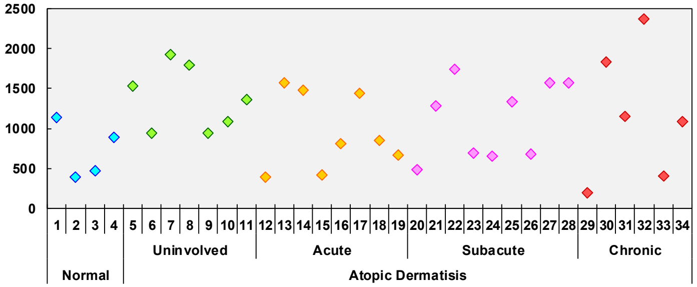

ERCC5

excision repair cross-complementation group 5

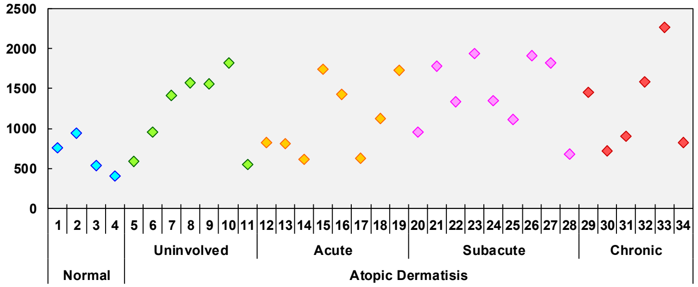

ARID4B /// RBM34

AT rich interactive domain 4B (RBP1-like) /// RNA binding motif protein 34

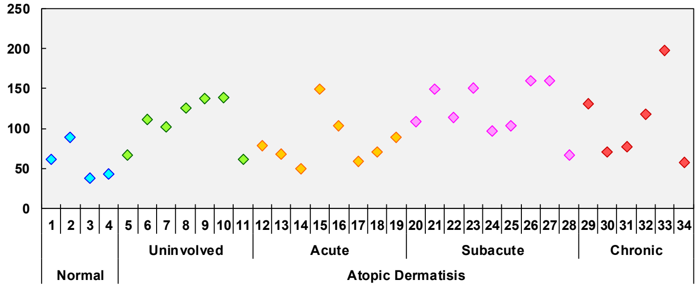

Up regulated in only uninvolved skin in AD patients (group 1)

RAPGEF3

Rap guanine nucleotide exchange factor 3

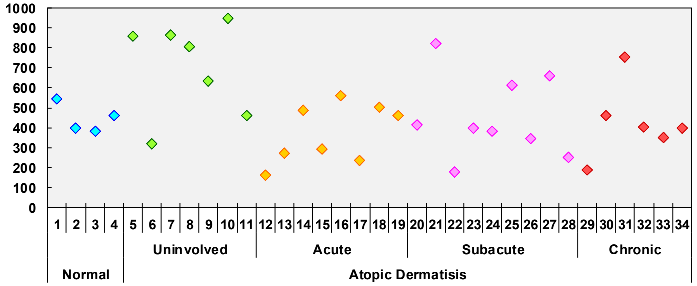

MYH11

myosin, heavy chain 11, smooth muscle

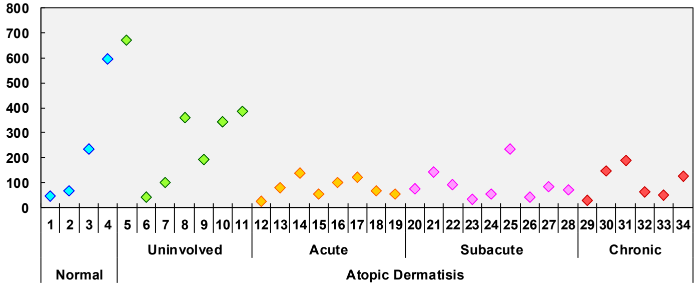

ATP1B1

ATPase, Na+/K+ transporting, beta 1 polypeptide

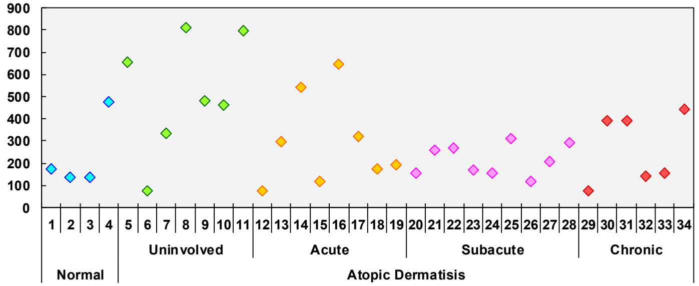

MSMO1

methylsterol monooxygenase 1

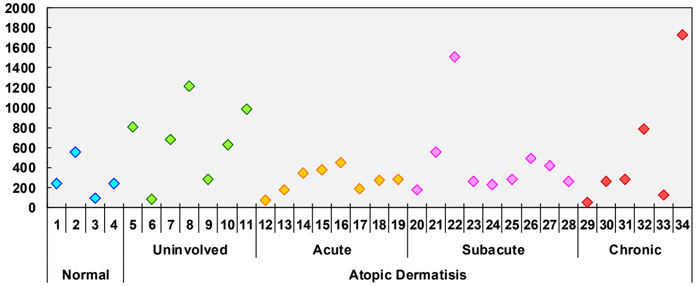

PIP

prolactin-induced protein

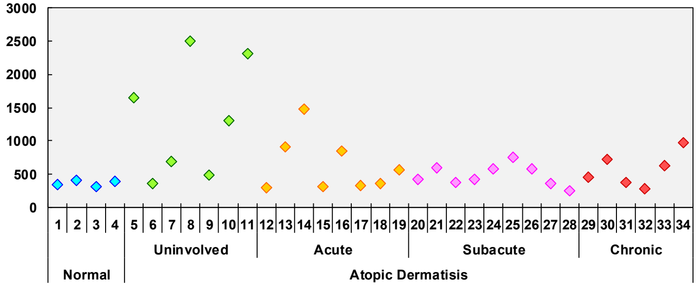

FADS1

fatty acid desaturase 1

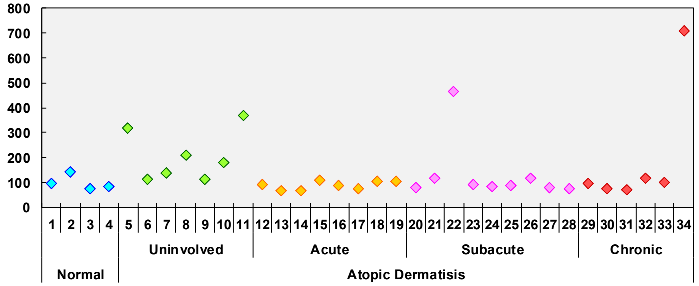

MUC1

mucin 1, cell surface associated

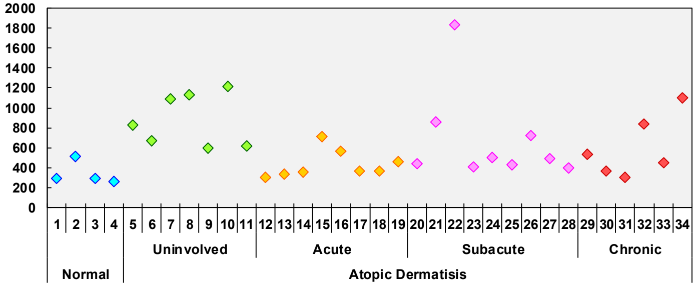

SCGB1D2

secretoglobin, family 1D, member 2

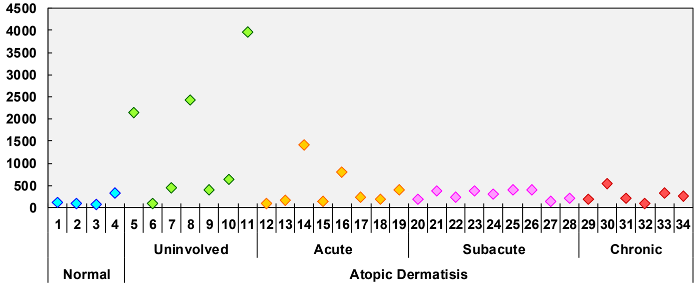

MED16

mediator complex subunit 16

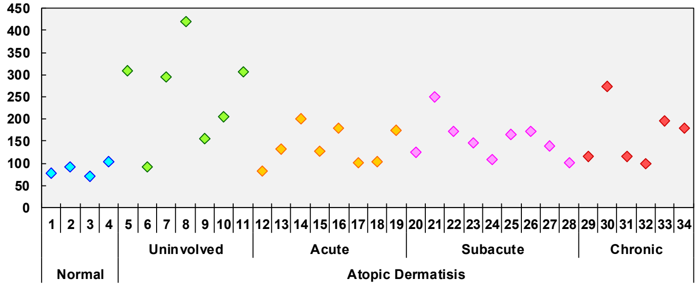

Up regulated in only uninvolved skin in AD patients (group 1) -continued

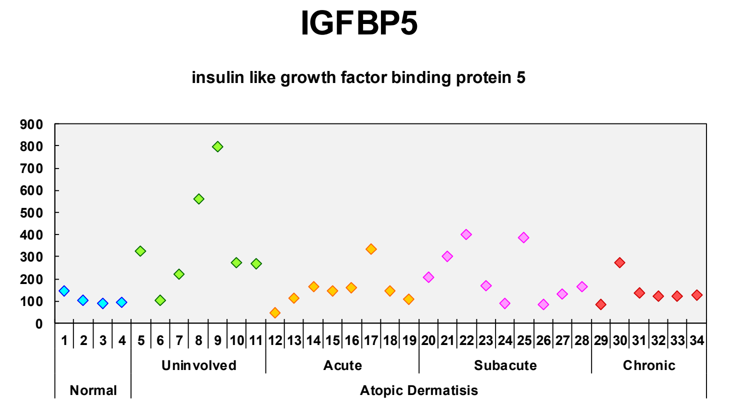

Up regulated in healthy control and uninvolved skin in AD patients

KRT15

keratin 15, type I

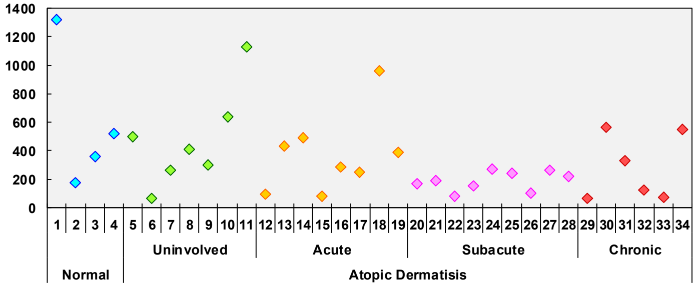

GABARAPL1 /// GABARAPL3

GABA(A) receptor-associated protein like 1 /// GABA(A) receptors associated protein like 3, pseudogene

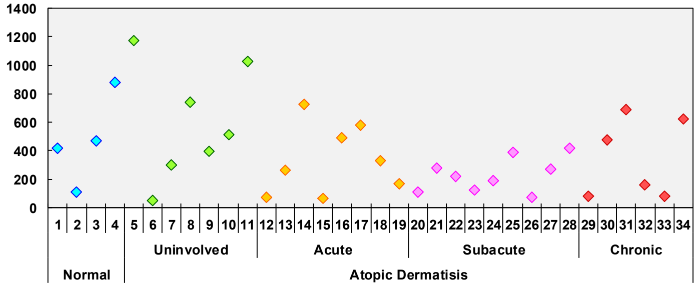

MYH11

myosin, heavy chain 11, smooth muscle

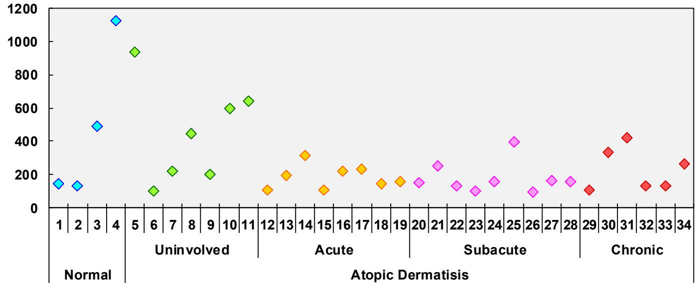

HBA2

hemoglobin, alpha 2

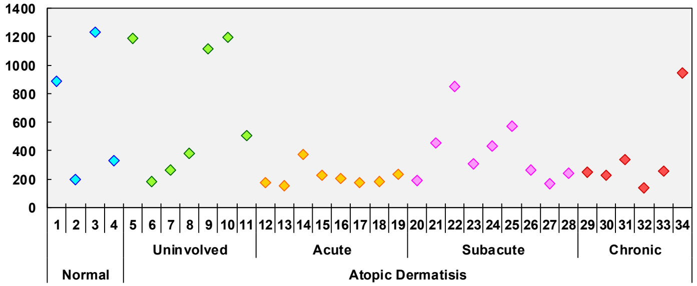

HBA1 /// HBA2

hemoglobin, alpha 1 /// hemoglobin, alpha 2

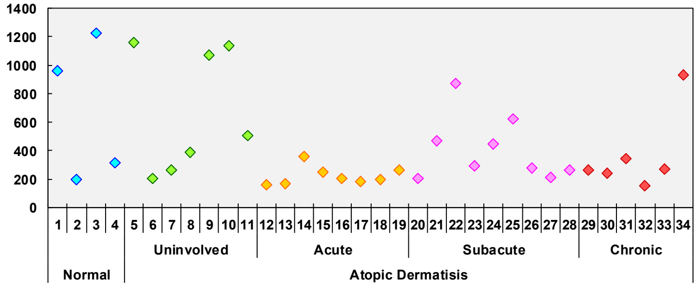

HBA1 /// HBA2

hemoglobin, alpha 1 /// hemoglobin, alpha 2

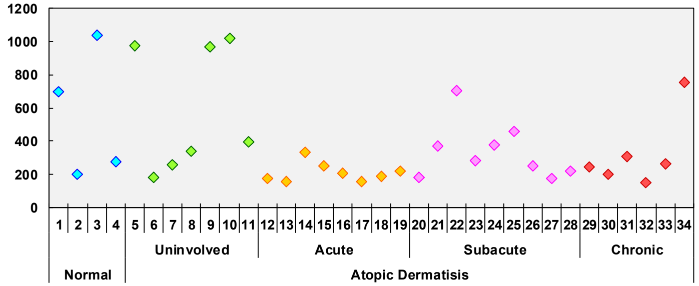

HBA1 /// HBA2

hemoglobin, alpha 1 /// hemoglobin, alpha 2

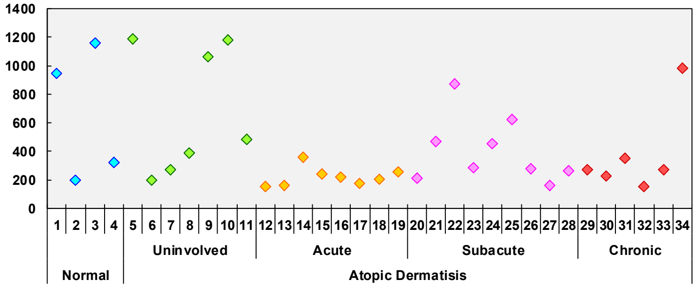

HBA1 /// HBA2

hemoglobin, alpha 1 /// hemoglobin, alpha 2

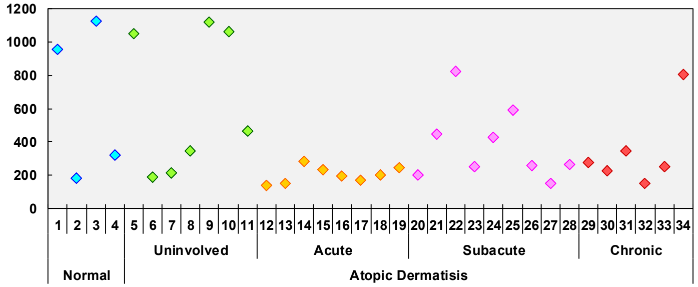

TCF7L2

transcription factor 7-like 2 (T-cell specific, HMG-box)

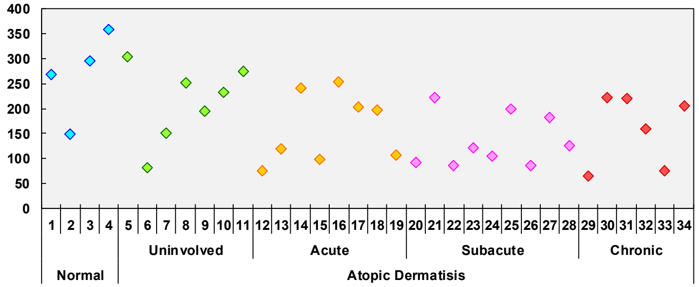

Down regulated in both uninvolved and involved skin in AD patients (group2)

CDHR1

cadherin-related family member 1

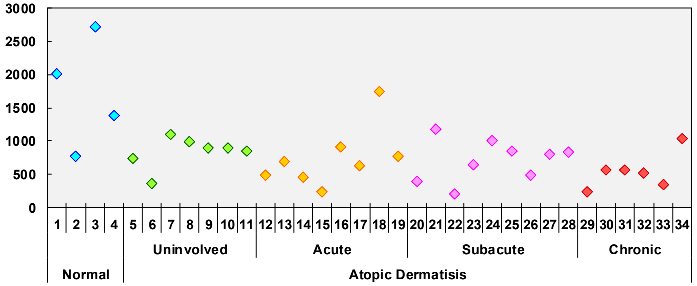

KLF2

Kruppel-like factor 2

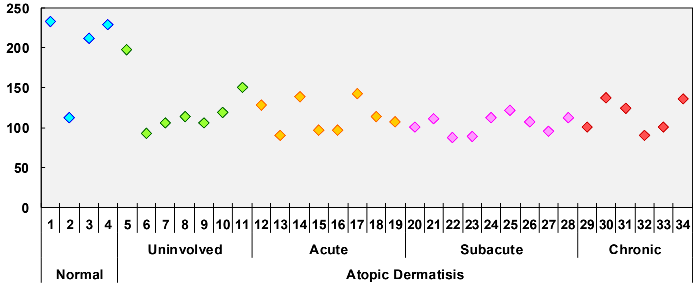

TNNI2

troponin I type 2 (skeletal, fast)

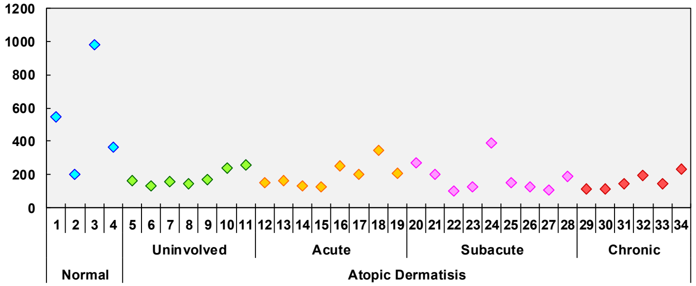

EGR1

early growth response 1

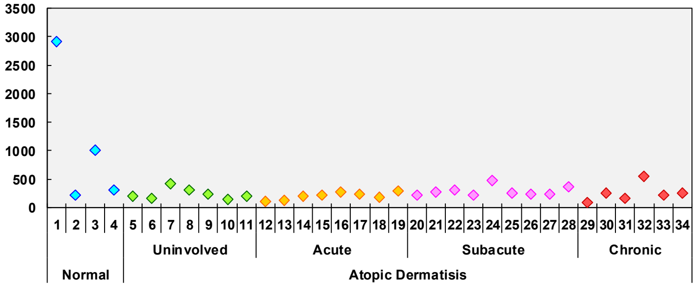

LOC100272216

uncharacterized LOC100272216

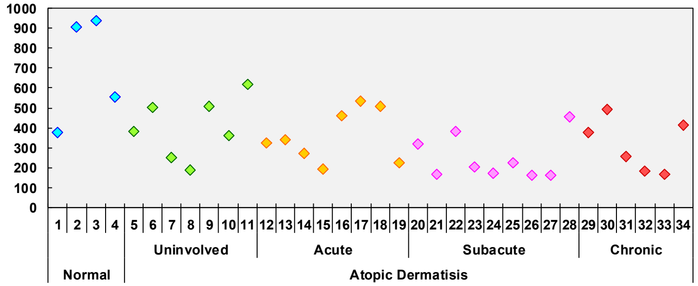

BGN

biglycan

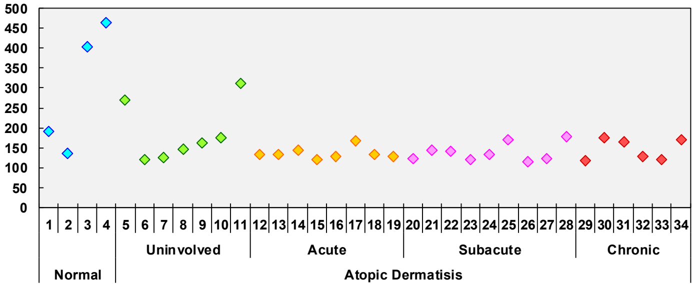

BGN

biglycan

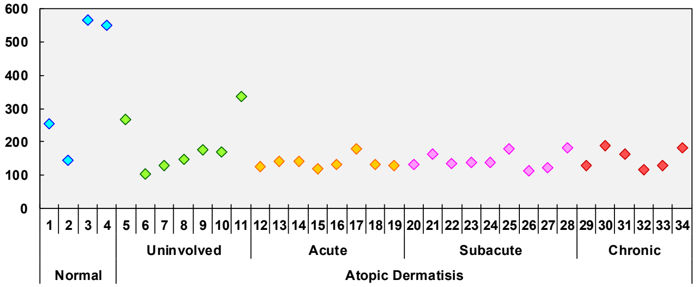

WIF1

WNT inhibitory factor 1

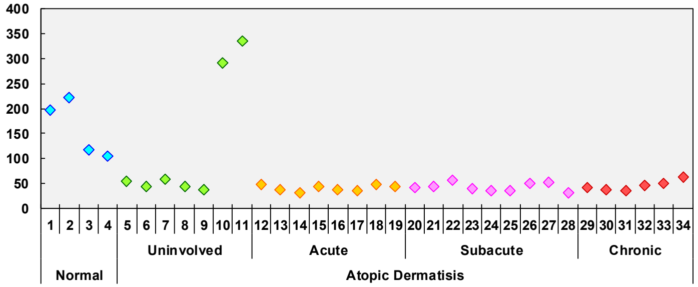

ZNF273

zinc finger protein 273

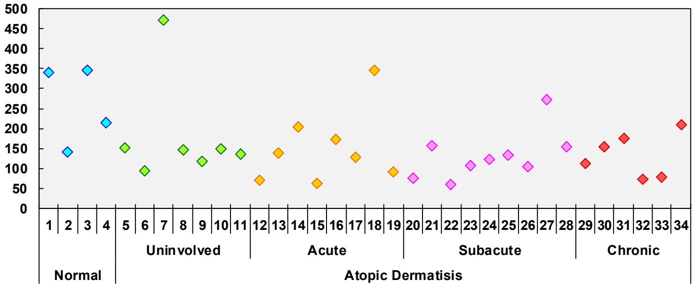

Down regulated in both uninvolved and involved skin in AD patients (group2) -continued

FN1

fibronectin 1

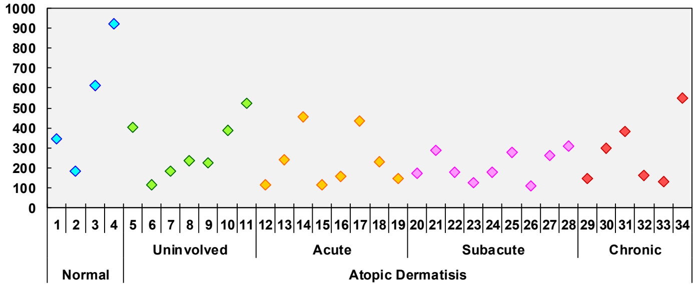

ID4

inhibitor of DNA binding 4, dominant negative helix-loop-helix protein

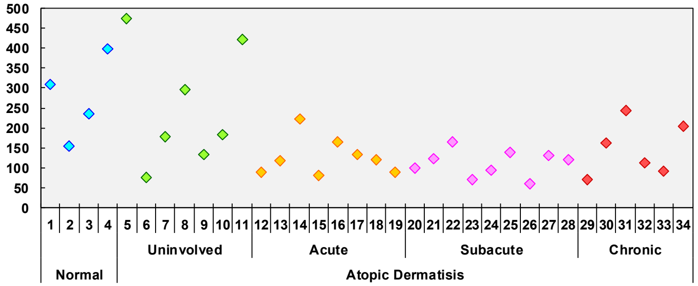

LOR

loricrin

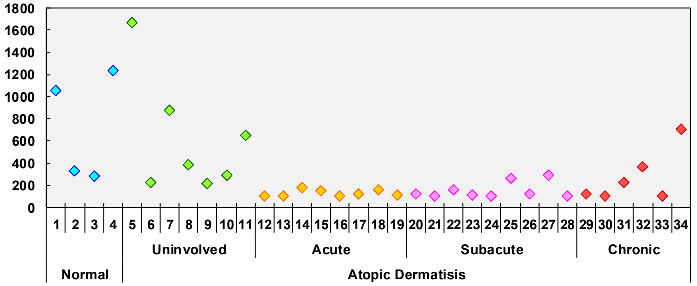

LEPR

leptin receptor

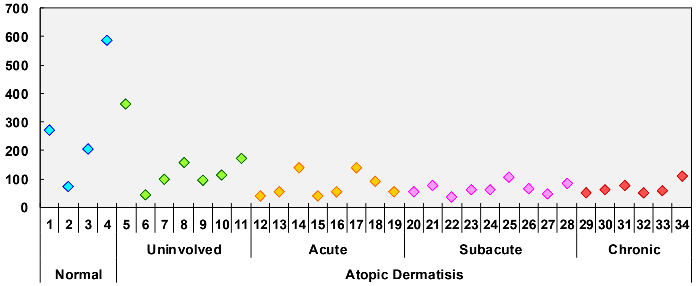

PCSK5

proprotein convertase subtilisin/kexin type 5

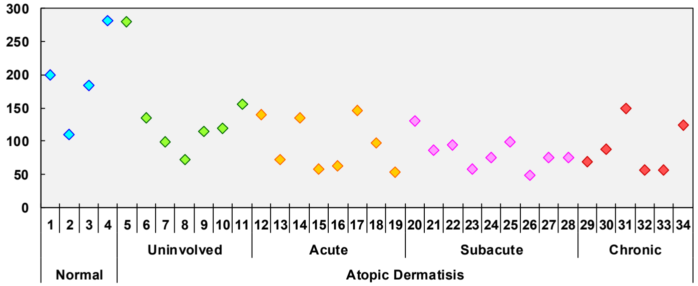

FHL1

four and a half LIM domains 1

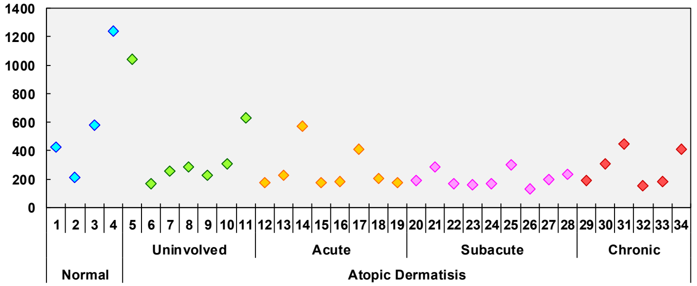

FLG

filaggrin

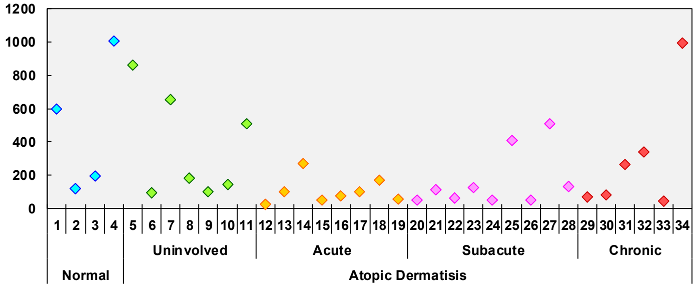

CYP1B1

cytochrome P450, family 1, subfamily B, polypeptide 1

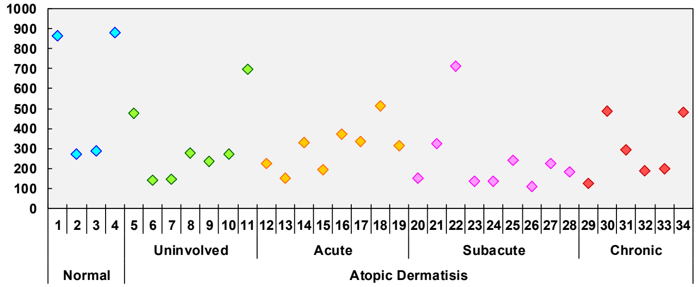

PURA

purine-rich element binding protein A

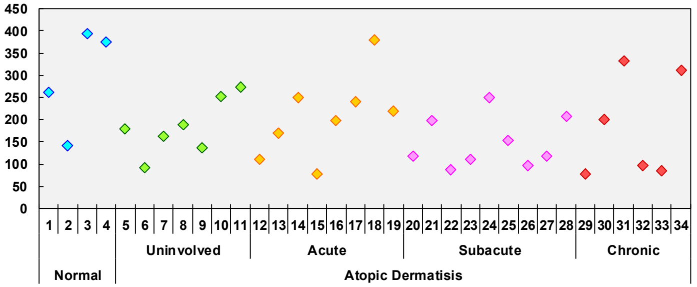

Up regulated both uninvolved and involved skin in AD patients (group 3)

SP110

SP110 nuclear body protein

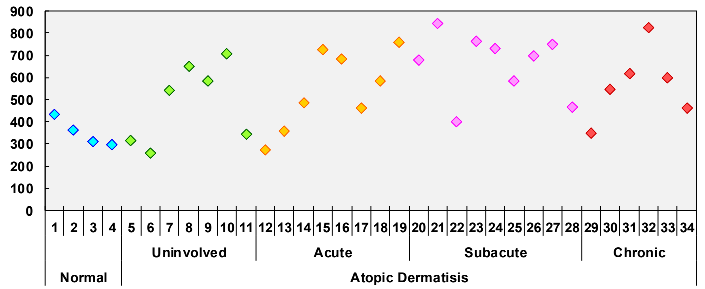

VDAC1

voltage-dependent anion channel 1

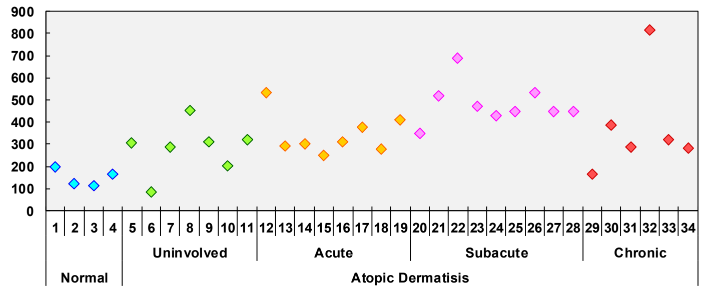

APLP2

amyloid beta (A4) precursor-like protein 2

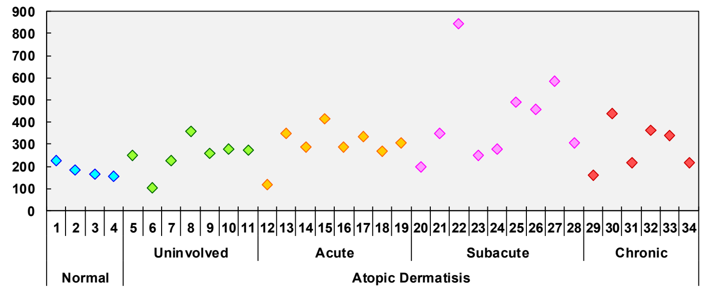

B2M

beta-2-microglobulin

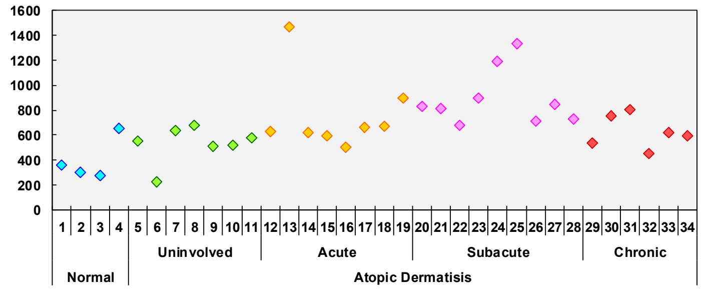

HSPA9

heat shock 70kDa protein 9 (mortalin)

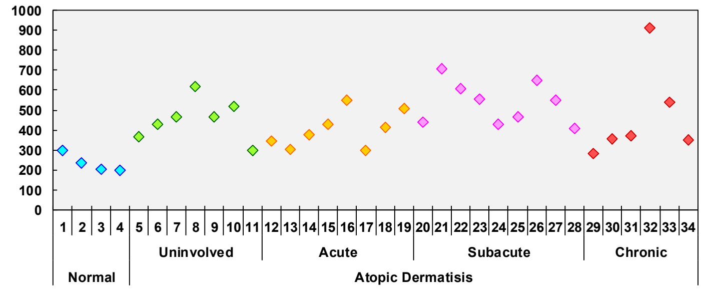

EIF5B

eukaryotic translation initiation factor 5B

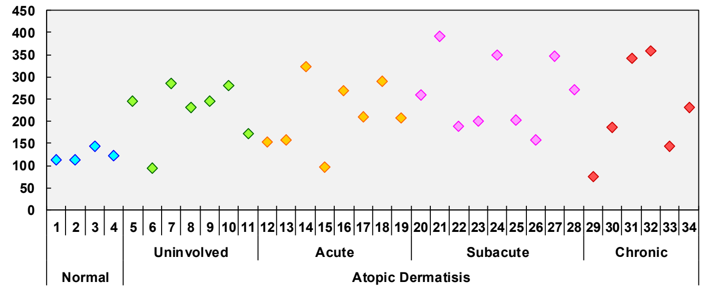

PTP4A2

protein tyrosine phosphatase type IVA, member 2

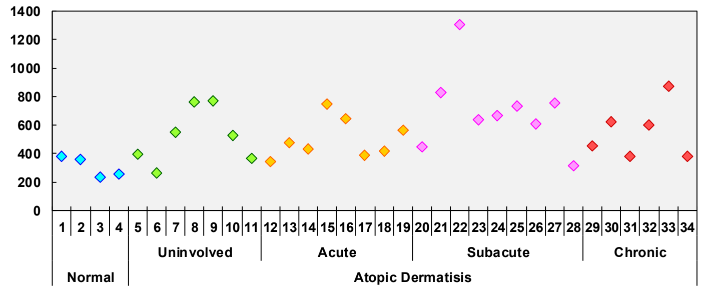

HNRNPC

heterogeneous nuclear ribonucleoprotein C (C1/C2)

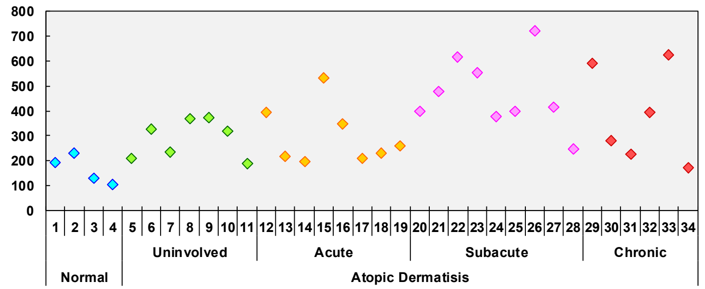

DDX1

DEAD (Asp-Glu-Ala-Asp) box helicase 1

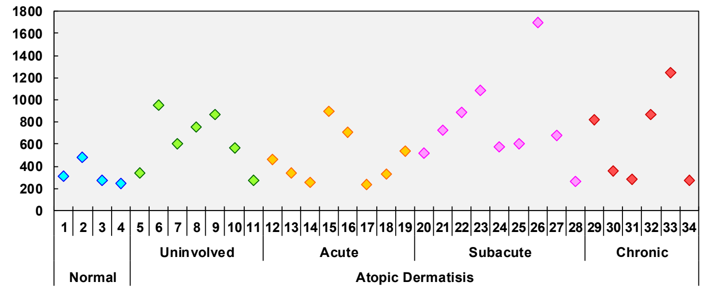

Up regulated both uninvolved and involved skin in AD patients (group 3) -continued

NAB1

NGFI-A binding protein 1

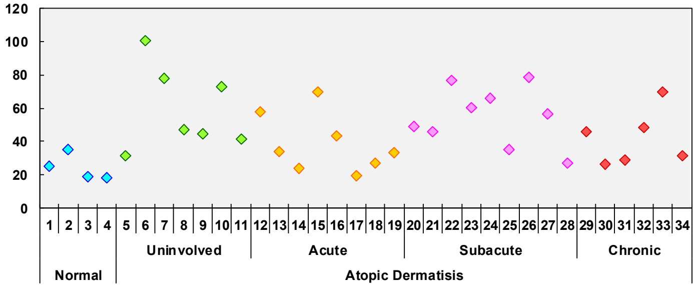

HSP90B1

heat shock protein 90kDa beta (Grp94), member 1

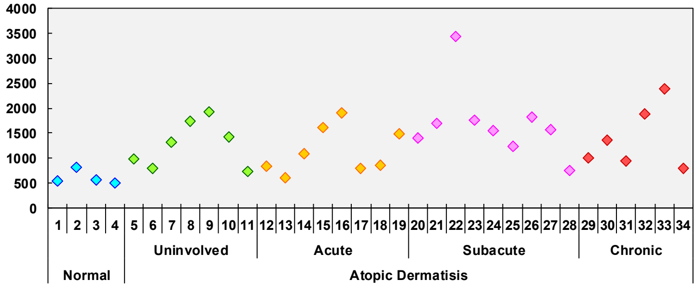

CANX

calnexin

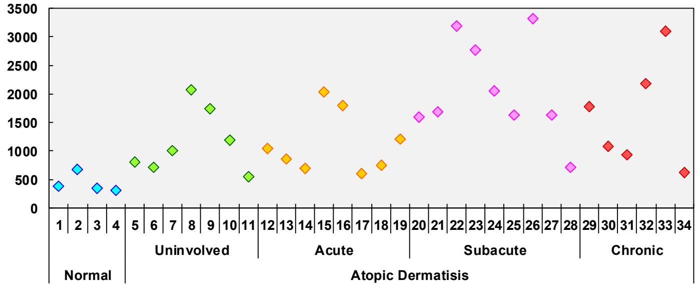

RNF11

ring finger protein 11

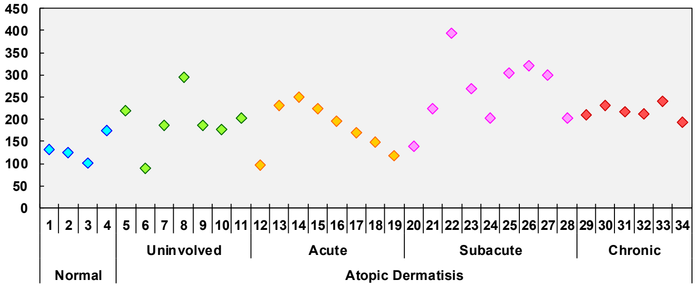

MLEC

malectin

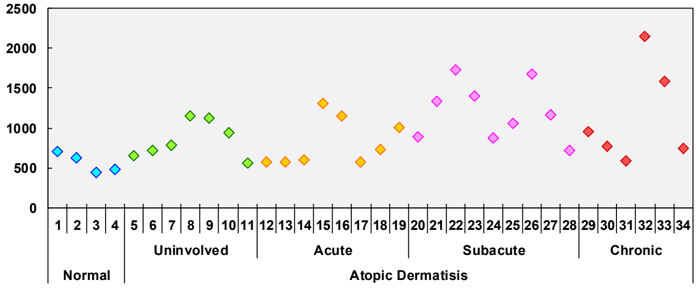

Up regulated in involved skin in AD patients (group 4)

SERPINB3

serpin peptidase inhibitor, clade B (ovalbumin), member 3

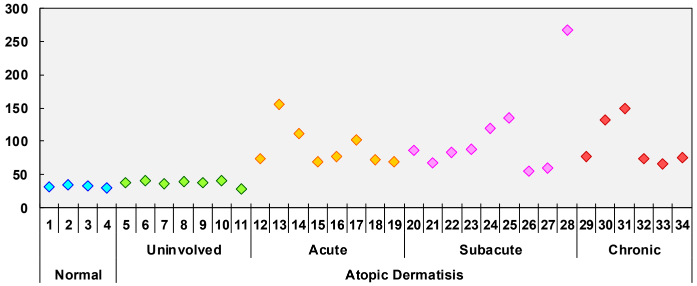

S100A2

S100 calcium binding protein A2

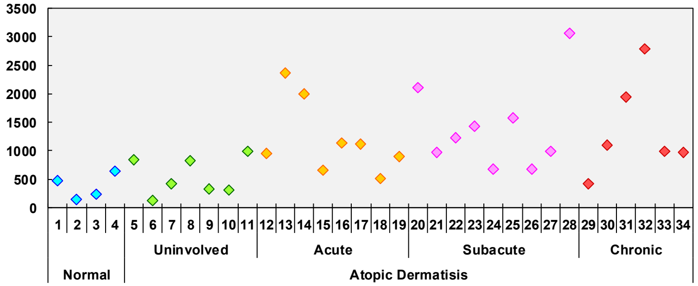

FABP5

fatty acid binding protein 5 (psoriasis-associated)

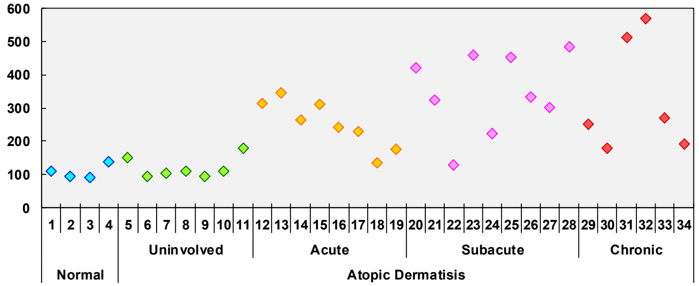

TYMP

thymidine phosphorylase

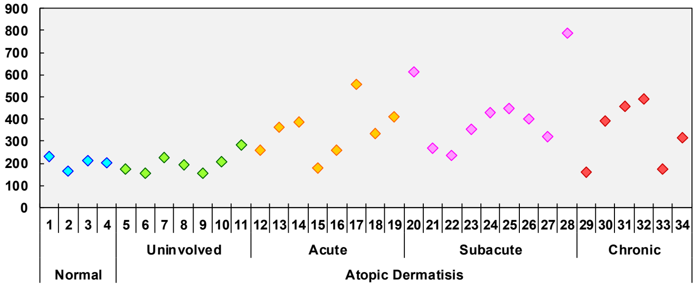

EHF

ets homologous factor

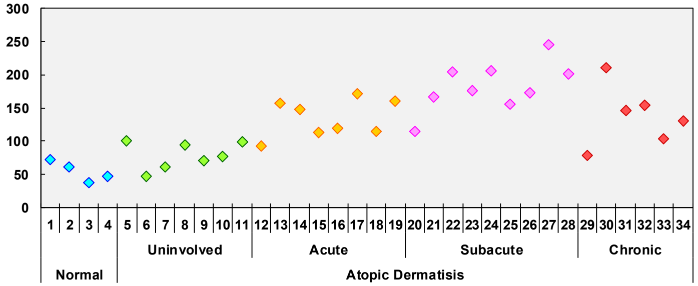

DSC2

desmocollin 2

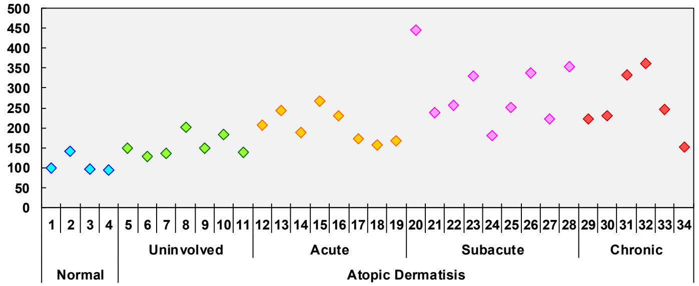

IL1RN

interleukin 1 receptor antagonist

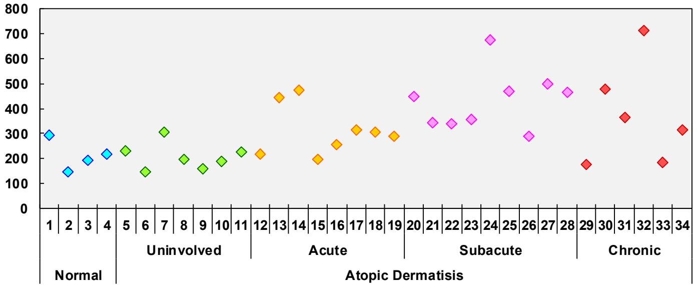

S100A9

S100 calcium binding protein A9

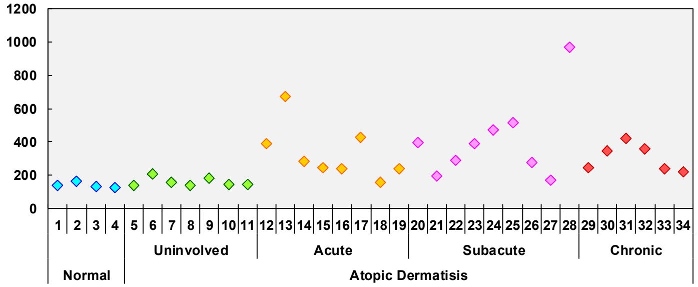

KRT6B

keratin 6B, type II

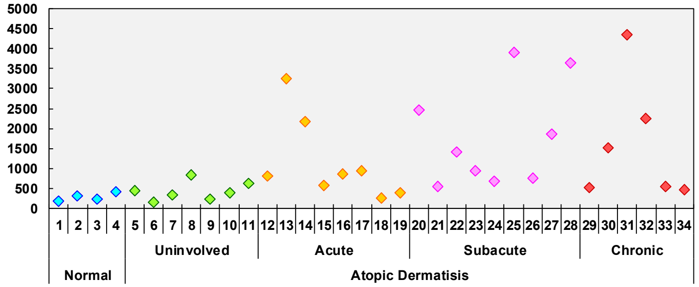

Up regulated in involved skin in AD patients (group 4)-continued

CLCA2

chloride channel accessory 2

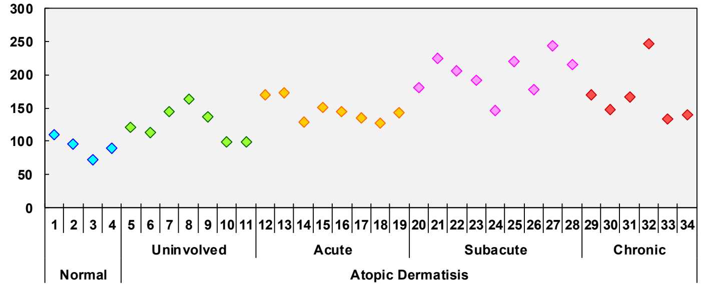

NMI

N-myc (and STAT) interactor

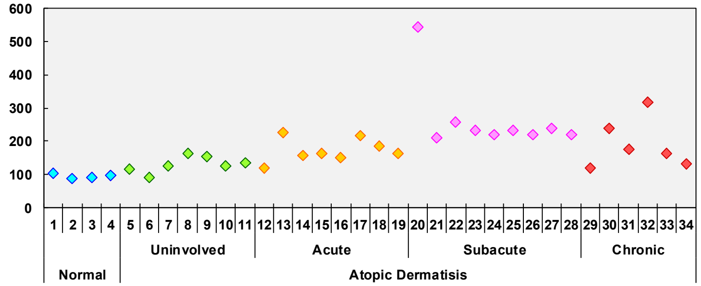

S100A8

S100 calcium binding protein A8

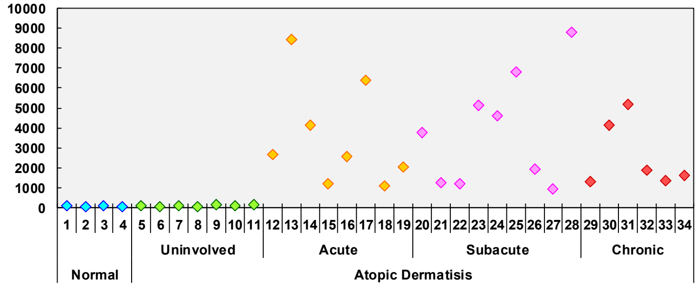

KRT16

keratin 16, type I

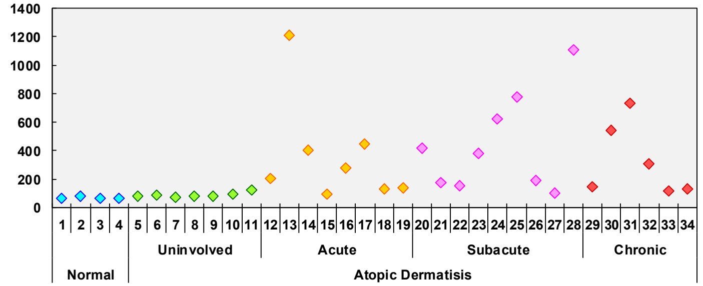

MMP12

matrix metallopeptidase 12

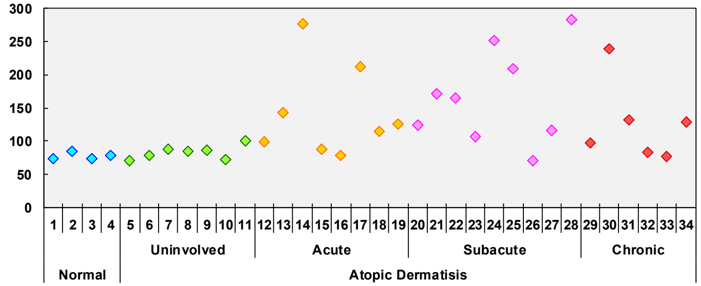

TOP2A

topoisomerase (DNA) II alpha

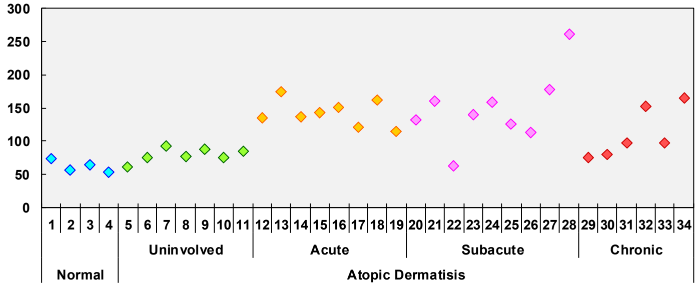

KRT6A /// KRT6B /// KRT6C

keratin 6A, type II /// keratin 6B, type II /// keratin 6C, type II

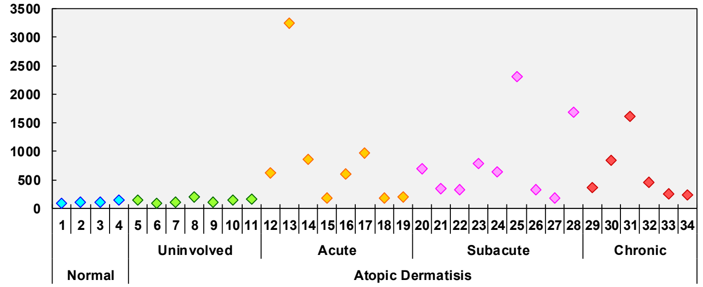

PRRC2C

proline-rich coiled-coil 2C

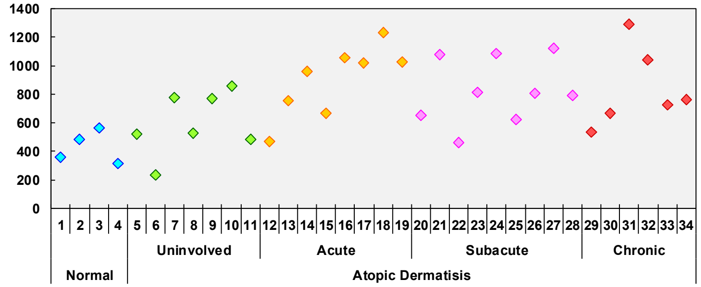

DUOX1

dual oxidase 1

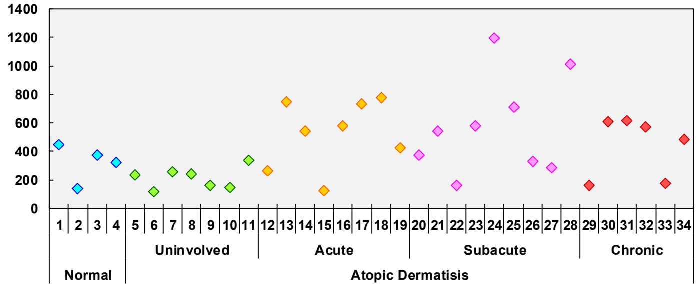

Up regulated in involved skin in AD patients (group 4)-continued

SCO2

SCO2 cytochrome c oxidase assembly protein

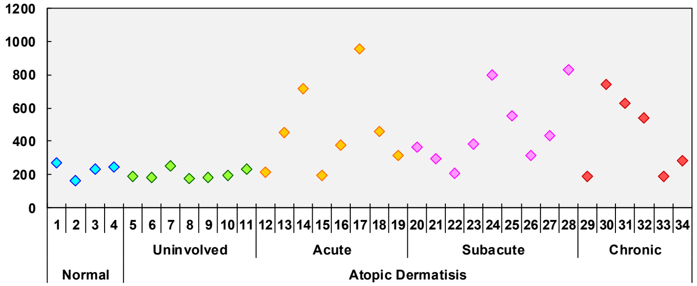

SERPINB13

serpin peptidase inhibitor, clade B (ovalbumin), member 13

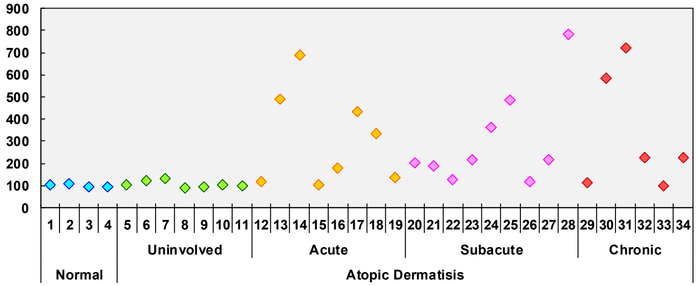

KRT6A

keratin 6A, type II

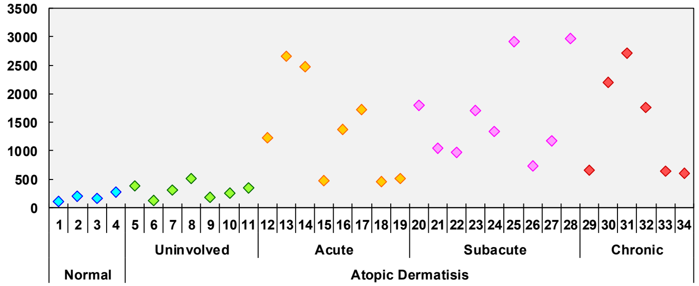

ADAM8

ADAM metallopeptidase domain 8

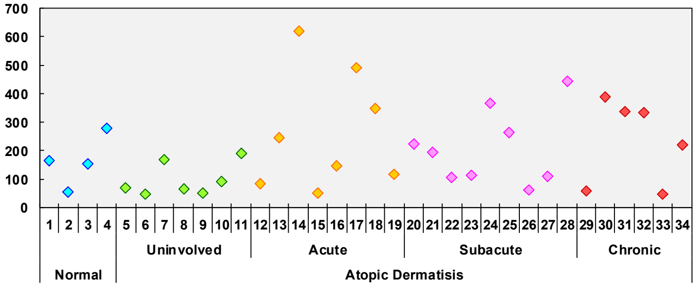

SPRR1A

small proline-rich protein 1A

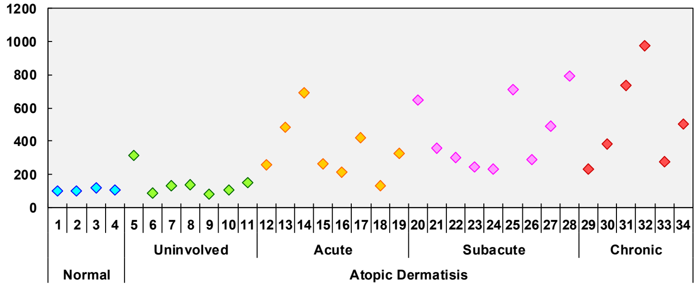

IFI16

interferon, gamma-inducible protein 16

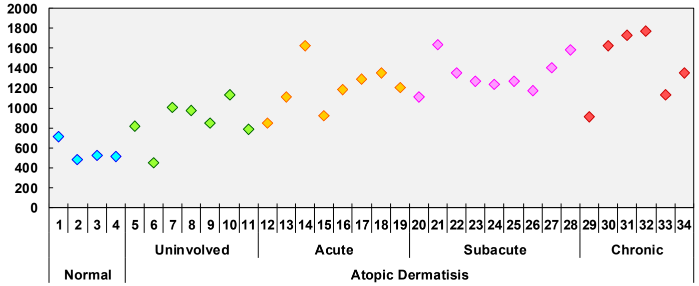

NOD2

nucleotide-binding oligomerization domain containing 2

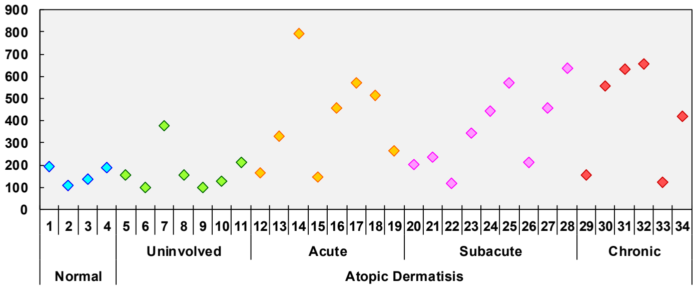

S100A7

S100 calcium binding protein A7

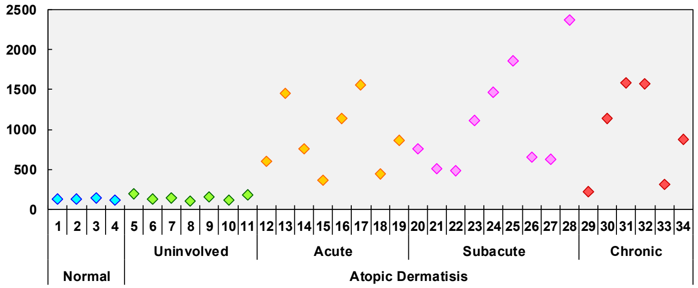

SERPINB13

serpin peptidase inhibitor, clade B (ovalbumin), member 13

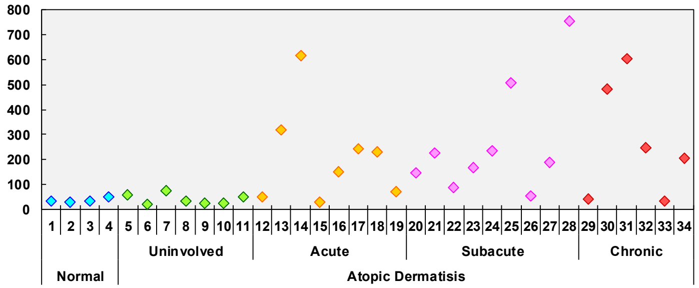

Up regulated in involved skin in AD patients (group 4)-continued

ANGPTL4

angiopoietin like 4

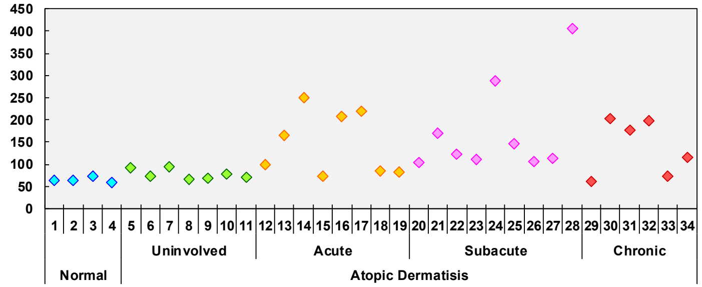

TNFSF10

tumor necrosis factor (ligand) superfamily, member 10

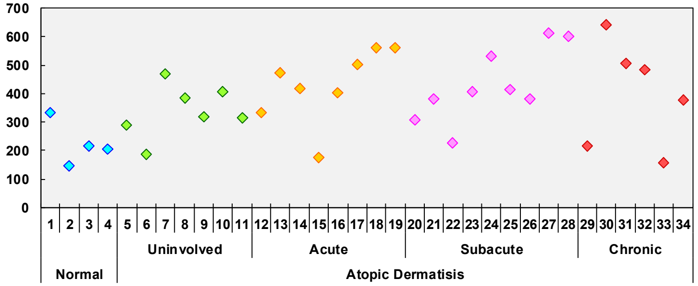

CD24

CD24 molecule

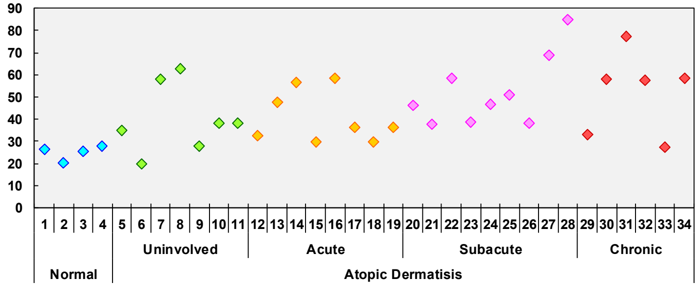

CBX3

chromobox homolog 3

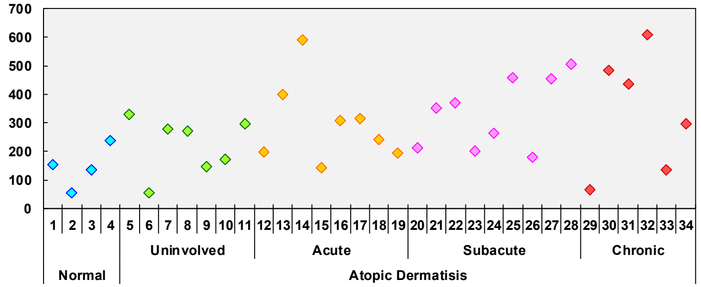

HIF1A

hypoxia inducible factor 1, alpha subunit (basic helix-loop-helix transcription factor)

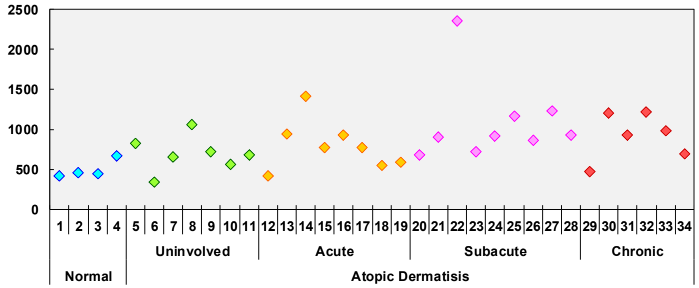

MICROARRAY ANALYSIS

Genes with altered expression only in non-lesional areas

Genes with altered expression only in non-lesional areas may be involved in maintaining healthy skin against constitutional mutations in AD. Multiple genes (MSMO1, FADS1, SCGB1D2) thought to be related to lipids and steroids were also included. A gene related to thyroid hormone (MED16) and a gene related to mucous barriers and intercellular signaling (MUC1) were also included (Figure 2). In addition, the expression of prolactin-induced protein (PIP), which has both local and systemic immunosuppressive effects, a proteolytic effect on the stratum corneum, and keratinocyte proliferation-inducing functions, was also enhanced.

Table S2. Outline of the Intensity of Immunohistochemistry (IHC).

**Gene Name**	**Chaperone and MHC Class I**				**Gripping Function**	**Consistency with Microarray Analysis**
	Normal control	Uninvolved	Involved	Clinical control		
**HSP90B1** (epidermis)	+	+	++	+	ER master chaperone	○
HSP90B1(dermis)	+	+	++	+		
**HSPA9**	Not consistent	Not consistent	Not consistent	Not consistent	Stress response chaperone	×
**CANX** (epidermis)	+	++	+++	++++	ER chaperone regulate MHC1	○
CANX (dermis)	+	++	+++	++++		
**B2M** (epidermis)	−～Weak	Weak～+	−～Weak～+	+	MHC1 association	○
B2M (dermis)	−	+	+～++	+～++		

	**T cell-related**					
	Normal control	Uninvolved	Involved	Clinical control		
**BGN** (epidermis)	+	Weak	Weak	+	Structure/trigger of Th1 and Th17	○
BGN (dermis)	+	Weak	Weak	+		
**KLF2** (epidermis)	+	Weak	Weak	++	CD4/CD8 T cell maturation<br>T cell quiescence	○
KLF2 (dermis)	+	Weak	Weak	++		
**LEPR** (epidermis)	Weak	Very weak	Very weak	+	Shifting of T cell toward Th1	○
LEPR (dermis)	Weak	Very weak	Very weak	+		
	**Cell Growth**					
	Normal control	Uninvolved	Involved	Clinical control		
**NAB1**	+	++	++	+++	EGR1 binding protein	○
**EGR1**	+	Very weak	Weak	+	Low expression in resting cells	○
**WIF1**	+	Weak	Weak	Weak～+	Negative regulation of keratinocyte proliferation	○

The intensity of staining was independently evaluated by three observers (H.S., S.S., and Y.H.). IHC showed that the protein expression of molecular chaperones (HSP90B1 and CANX), major histocompatibility complex (MHC) class I-related genes (HSP90B1, CANX, B2M), genes related to T cell maturation and differentiation (BGN, KLF2, LEPR), and cell growth genes (NAB1, EGR1, WIF1) in lesional and non-lesional areas was similar to the gene expression observed in the microarray study. However, the protein expression of HSPA9 was inconsistent with the microarray results. Dermal and epidermal cells showed increased expression of HSP90B1, CANX, and B2M, and decreased expression of BGN, KLF2, and LEPR. The nuclei of epidermal cells expressed NAB1 or EGR1 protein, but not both.

B2M, beta-2-microglobulin; BGN, biglycan; CANX, calnexin; EGR1, early growth response 1; ER, endoplasmic reticulum; HSPA9, heat shock protein family A (Hsp70) member 9; HSP90B1, heat shock protein 90 beta family member 1; KLF2, Kruppel like factor 2; LEPR, leptin receptor; NAB1, NGFI-A binding protein 1; Th1, T helper 1; WIF1, WNT inhibitory factor 1

X: indicates that the IHC data does not have the same trend as the microarray data.

O: Indicates that the IHC data has the same trend as the microarray data.

IHC ANALYSIS

EGR1 is a potent regulator of NAB2 transcription. By identifying a major regulatory element in the promoter of NAB2,[1] the present IHC and microarray study showed that cells expressed NAB1 or EGR1 (protein and gene levels), but not both.

Immunofluorescence study for CD3/KLF2 double staining

through confocal laser scanning microscopy (Leica TCS SP8 X; Leica, Wetzlar, Germany). KLF2 was stained using anti-KLF2 antibody (rabbit) (1:1,000 dilution; COSOMO BIO Co., LTD, Tokyo, Japan) as the primary antibody and donkey anti-rabbit IgG antibody Cy3 (AP182C; 1:1,000 dilution; Merck, Darmstadt, Germany) as the secondary antibody.

CD3 was stained using anti-CD3 antibody (mouse) (1:1000 dilution; Abcam, Cambridge, UK) as the primary antibody and donkey anti-mouse IgG H&L (Alexa Fluor® 488; Abcam) as the secondary antibody (1:1,000 dilution). Many CD3-positive infiltrating cells are stained with KLF2.

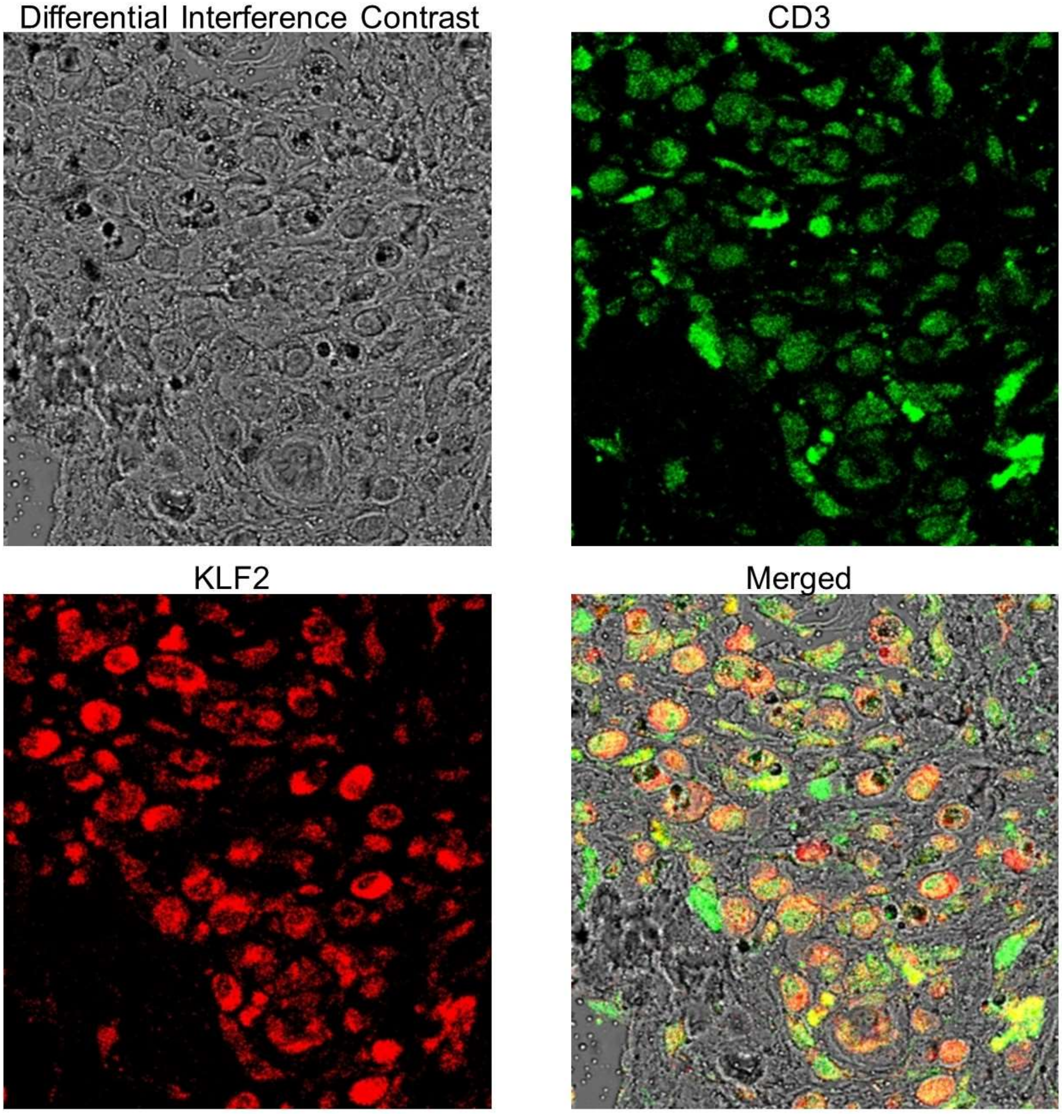

Figure S2. Immunofluorescence study for CD3/KLF2 double staining

Selected co-stained images showing that KLF2 (red) is expressed on CD3 positive cells (green) in the dermis. As shown in merged figure, many CD3-positive infiltrating cells are stained with KLF2.

Discussion on genes thought to play an important role in the pathogenesis of AD, but not introduced in the main text.

In the main text, we have explained that overload of the intracellular stress response may be one of factors to easily exacerbate and relapse AD.

In this Supporting Information text, we introduced some other candidate genes for atopic dermatitis that are thought to play an important role, as well as discoveries and ideas that may lead to an understanding of the pathogenesis of the disease.

LIPID METABOLISM

In our microarray analysis of AD, fluctuations in the expression of multiple genes related to lipids and cholesterol were observed. Increased expression of MSMO1 and FADS1 was observed in the non-lesioned area. In addition, consistent reductions in the expression of KLF2, LEPR, PCSK5, and CYP1B1 were observed in lesioned and non-lesioned areas.

FADS1 is an aggravating factor for AD.[2] In addition, a study reported that the prevalence of AD and allergic rhinitis decreases when the single nucleotide polymorphism of FADS1 is present.[3]

PCSK5 regulates prohormone, lipids, and sterol through inactivation of lipase. Inhibition of CYP1B1 is effective in the treatment of obesity, hypertension, and atherosclerosis.[4] However, there is consistently reduced expression of CYP1B1 in lesioned and non-lesioned areas in patients with AD. If this reduced expression is systemic beyond the skin, patients with AD may be at a lower risk of developing hypertension, obesity, and atherosclerosis. Uehara et al. reported that blood pressure is lower in patients with AD than in healthy controls.[5]

Abnormalities related to lipids and cholesterol may be related to the lack of a lipid barrier in the stratum corneum. In AD, the lipid balance in the stratum corneum is

disrupted and the water content capacity is reduced.[6] In addition, CYP1B1, which may cause disturbance in the balance of endogenous steroid hormones, is related to the synthesis pathway and metabolism of estrogen, testosterone, and progesterone.

HYPERTENSION IS RARE IN ADULT PATIENTS WITH AD

Hypertension is rare in adult patients with AD.[5] Nevertheless, the mechanism involved in this effect has not been clarified. CYP1B1 causes hypertension and its decreased expression may be associated with the occurrence of hypotension in patients with AD.

POSSIBLE CAUSE OF Th1/Th2 IMBALANCE

The expression of Th2 is higher than that of Th1, and treatment with interleukin 4 (IL4)/IL13 receptor antagonist has demonstrated a marked therapeutic effect in patients with AD. Thus, increased expression of Th2 plays an important role in the pathogenesis of AD. It has been shown that BGN (Th1 and Th17) and Leptin (Th1) shift the Th1/Th2 balance toward Th1.

The present microarray results showed that the expression of BGN and LEPR decreased in lesioned and non-lesioned areas in patients with AD. Immunostaining demonstrated that BGN and LEPR expression decreased in AD; notably, BGN expression increased in psoriatic lesions. Based on the results of the microarray and immunostaining analyses, it is suggested that the decrease in the expression of BGN and LEPR shifts the Th1/Th2 balance toward Th2 in AD.

GENES WITH INCREASED EXPRESSION ONLY IN NON-LESIONAL AREAS, PARTICULARLY THE FUNCTION OF PIP

The microarray analysis revealed genes with increased expression only in non-lesional areas of patients with AD. These genes play an important role in maintaining the unaffected area healthy. Among these genes, we selected PIP to conduct two functional studies.

Human PIP contains a sequence that binds to CD4 and inhibits the binding of CD4-human leukocyte antigen-antigen D related (CD4-HLA-DR). Furthermore, in mouse contact dermatitis, the CD4-binding sequence of mouse PIP acts to suppress inflammation locally and systemically.[7] Nevertheless, PIP contains an aspartate peptidase sequence in addition to the CD4-binding site, which disrupts the skin barrier and induces epidermal cell proliferation.[8] In AD, the immunosuppressive effect of PIP may be limited due to T cell or subsequent signaling problems, while the function of the enzyme sequence as a deteriorating factor for the skin is significant.

Apart from PIP, genes with increased expression only in non-lesional areas of AD included sterol-C4-methyl oxidase-like (SC4MOL), FADS1, MUC1 transmembrane, and thyroid hormone receptor-associated protein 95-kD subunit. These genes may be important targets for studying the pathology of AD and biochemical

characteristics of patients with AD.

MHC I ENHANCEMENT AND AUTOIMMUNITY IN AD

MHCI-related molecules, such as CANX, B2M, HSP90B1, and APLP2, showed increased expression in the microarray analysis (Figure 2). Moreover, increased expression of CANX and B2M was confirmed by immunostaining (Figure 3A). It has been reported that the 6p21.3 locus (the locus of MHCI) has a genetic linkage with AD.[9] MHCI and MHCII correspond to intracellular and extracellular antigens, respectively. The increase in MHCI-related gene expression suggests the presence of misfolding proteins in cells. Intracellular misfolding proteins may be recognized as self-antigens by MHCI. Autoimmunity may occur if the antigenic site is exposed to the surface with a normal protein higher-order structure. As another basic condition related to autoimmunity, the expression of KLF2, which exerts a quiescence effect on T lymphocytes, was decreased in diseased skin of AD versus healthy skin.[10] Although the identity of the abnormal protein was not disclosed in this study, the reaction to self-antigens may relate to vitiligo often observed in recalcitrant lesions of AD and the pathogenesis of alopecia totalis in young patients with AD. The upregulation of B2M and APLP2 may relate to the accumulation of amyloid in chronic skin lesions of adult patients with AD (see Supporting Information).[11]

AMYLOID DEPOSITION

Accumulation of misfolding β-sheet causes amyloidosis. Of note, B2M also has a β-sheet structure.[11] Currently, the misfolding or accumulation of B2M in tissues of patients with AD has not been demonstrated. Nevertheless, an increase in B2M in tissues of patients with AD may cause lichen amyloidosis. Lichen amyloydosis is frequently observed in the forearms and lower legs of adult patients with chronic and recalcitrant AD.

Amyloid precursor protein is involved in amyloid deposition. Amyloid beta precursor-like protein 2 (APLP2), which showed consistently increased expression in lesioned and non-lesioned areas in the microarray analysis, has been studied for homology with amyloid precursor protein.[12] Increased expression of APLP2 in patients with AD may cause amyloid deposition.

CLOSE INTERACTION AND GENE LOCUS BETWEEN NAB1 AND EGR1

NAB1 exerts an inhibitory effect on EGR1.[1] Both our microarray and immunostaining analyses consistently showed an increase in NAB1 expression and a decrease in EGR1 expression in lesioned and non-lesioned areas in patients with AD. In sequential immunostaining, NAB1 and EGR1 were stained exclusively in the epidermis. NAB1 was mainly stained in the middle and lower epidermis, while EGR1 was mainly stained in the upper epidermis.

The 5q31 is an important locus in AD. In the present microarray analysis, HSPA9 (5q31.2)[13] was consistently upregulated in lesioned and non-lesioned areas. In contrast, EGR1 (5q31.2)[14] and PURA (5q31.3) [15] were consistently downregulated in lesioned and non-lesioned areas. NAB1[16] in 2q32.2 and EGR1 in 5q31.2 are functionally related,[17] but their relationship to PURA has not yet been studied.[18] However, their association with itching in AD has not been fully investigated.

DOES CHANGE IN THE EXPRESSION OF CHAPERONES, B2M, KLF2, BGN, AND LEPR OCCUR IN ORGANS OTHER THAN THE SKIN?

These genes are expressed in numerous organs other than the skin.[19] The decreased staining of BGN, KLF2, and LEPR in dermal cells suggests that the decrease in their functions may extend to organs other than the skin. The chaperones HSP90B1 and CANX were also upregulated in dermal infiltrating cells, suggesting that they may be upregulated both in the epidermis and systemically. These changes in expression may reflect the character of cells in individual patients. Table S3 shows some functions of BGN, KLF2, and LEPR in organs other than the skin.

WIF1 AND KERATINOCYTE PROLIFERATION

WIF1 is expressed by stem cells of the human interfollicular epidermis and negatively regulates the proliferation of keratinocytes.[20] According to our microarray and IHC data, WIF1 expression is consistently attenuated in lesions and non-lesioned areas. Decreased expression of WIF1 in AD may prolong epidermal cell proliferation and interfere with the natural healing of AD.

EXACERBATION AND SPREADING OF ECZEMA BY DAMPS

It is established that DAMPs (S100A7, S100A8, and S100A9) are secreted under stress, transmit dangerous signals to the outside of the cell, and induce cell proliferation.[21] The levels of S100A9 and S100A8 are increased in AD. In our microarray analysis, increased expression of numerous DAMPs, i.e., CD24,[22] NMI,[23] and HSP90B1 (binding to HMGB1), as well as S100A7, A8, and A9, was identified. These findings suggest that active eczematous lesions of AD induce the development of new eczema lesions in AD (Figure 2).

SPECIAL REMARKS

In this study, we analyzed non-lesioned areas from patients with active AD, but not from former patients with AD who have not experienced a flare-up of the skin rash all over the body for many years. Analysis of normal appearing skin from patients with active skin lesions will provide practical information for the treatment of AD.

Previous reports on HSP expression in AD skin lesions and healthy skin

Reports of increased HSP expression in AD skin lesions:

Ghoreishi M et al. reported that the expression of HSP65 and HSP72/73 was increased in skin lesions of AD patients compared with healthy controls and contact dermatitis.[24] This suggests that HSP65 and HSP72/73 may be involved in the pathogenesis of AD.

Expression and function of HSP in healthy skin:

HSPs are generally stress-induced proteins, and Scieglinska D et al. reported that HSPA1, HSPA2 and HSPC are physiologically expressed on keratinocyte in unstressed healthy skin by immunohistochemistry. [25] In this report, the physiological functions of each HSP on healthy skin keratinocyte are described as follows: HSPA1 is involved in the cytoprotection, HSPA2 contributes to the early steps of keratinocyte differentiation, while HSPC is essential in the reepithelialization process.

BIOPSY

Biopsy specimens were obtained from 23 involved skins (eight acute: erythema exsudatum, edema, occasionally with seropapules; nine subacute: indurated erythematous relapsing lesions; and six chronic: lichenified lesions). For one patient with AD, two skin samples (one acute and one subacute) were obtained. Seven uninvolved skin specimens were obtained from seven of the 22 patients with AD. Normal control skin samples were obtained from four normal controls. Background details are provided in Table S1. Representative clinical features of patients with AD are shown in Figure S1. Normal-appearing skin of patients with AD was defined as a smooth surface without scaling or erythematous change, and the biopsy site was ≥10 cm apart from active skin lesions. Thirty biopsy specimens from 22 patients with AD were divided into two parts: one part was sectioned using a cryostat for RNA extraction, while the other was fixed in 10% neutral buffered formalin, embedded in paraffin, and subsequently subjected to immunohistochemistry (IHC) analysis.

Table S3. Backgrounds of all AD patients examined both microarray analysis and Immunohistochemical (IHC) analysis.

No.	State	F/M	Age	IgE	Mite	House dust	Cedar	WBC	Eosino(%)	Facial lesion	Cataract	Clinical feature in Fig. S1	Representative IHC staining in Fig. 3a
1	Normal control	F	30-39										
2	Normal control	M	30-39										
3	Normal control	M	40-48										
4	Normal control	M	20-29										
5	non-lesion	M	30-39	14305.5	>100	92	54.6	7300	9.9	+	+	+	
6	non-lesion	M	15-19	5022.7	>100	>100		8300	14.7				KLF2, LEPR, WIF1
7	non-lesion	M	20-29	8059	>100	>100	63	8700	15.9			+	BGN
8	non-lesion	F	20-29	21986.7	>100	>100	21	5100	15.6				
9	non-lesion	M	15-19	15015.5	>100	>100	>100	7800	15.4				
10	non-lesion	M	20-29	1835	>100	>100	>100	4800	10.1	+	−	+	HSP90B1, HSPA9, CANX, NAB1, EGR1
11	non-lesion	M	20-29	8.8	<0.34	<0.34	<0.34	4900	2.7				B2M
12	acute	F	50-59	4125.5	>100	>100	47.4	6400	14.1	+	−	+	
13	acute	M	20-29	31653.3	>100	>100	>100	10300	3.7	+	+	+	
14	acute	M	40-49	29441.4	>100	>100	28.8	8100	24.5			+	
15(8)	acute	F	20-29	21986.7	>100	>100	21	5100	15.6				HSP90B1, NAB1, EGR1, WIF1
16(9)	acute	M	15-19	15015.5	>100	>100	>100	7800	15.4				
17	acute	F	50-59	759	>0.35	>0.35	>0.35	6100	10.7	+			
18	acute	F	30-39	343.3	>0.35	>0.35	>0.35	7800	8.6	+	−		
19(11)	acute	M	20-29	8.8	<0.34	<0.34	<0.34	4900	2.7				
20	sub acute	M	30-39	23395.4	<4.0			7300	6.1	+	+	+	
21	sub acute	F	15-19	10860.6	27.2	18.7	11.4	5100	9	−	−		
22	sub acute	F	30-39	5369	18.9	14.7	7.27	6500	12.5	+	−	+	
23	sub acute	M	30-39	77210.2	>100			5000	13.9	+	−		LEPR
24	sub acute	M	20-29	9261.2	>100	>100	>100	5300	18.7	+	−		
25	sub acute	M	20-29	6282	<4.0	<4.0		6600	10.2	+	+		
26	sub acute	M	20-29	13186	>100	>100	66	9000	23.1				
27(7)	sub acute	M	20-29	8059	>100	>100	63	8700	15.9			+	HSPA9, CANX, B2M, BGN, KLF2
28(17)	sub acute	F	50-59	759	>0.35	>0.35	>0.35	6100	10.7	+			
29	chronic	M	15-19	46879.6	>100	>100	9.41	9200		+	−		
30	chronic	M	15-19	7441				8800	5.7	+	−		
31(5)	chronic	M	30-39	14305.5	>100	92	54.6	7300	9.9	+	+		
32(6)	chronic	M	15-19	5022.7	>100	>100		8300	14.7				
33	chronic	M	30-39	265	11.1	9.82	39.4	7300	18.8	+	−	+	
34(10)	chronic	M	20-29	1835	>100	>100	>100	4800	10.1	+	−	+	

non-lesion: normal appearing skin , acute: erythema exsudatum, edema, sometimes with seropapules
subacute: indurated erythematous relapsing lesion , chronic: lichenified lesion

Two biopsy specimens were taken from 8 AD patients. In these cases, one of case number was indicated by parentheses.

Figure S3. Representative Clinical Features of Patients with AD

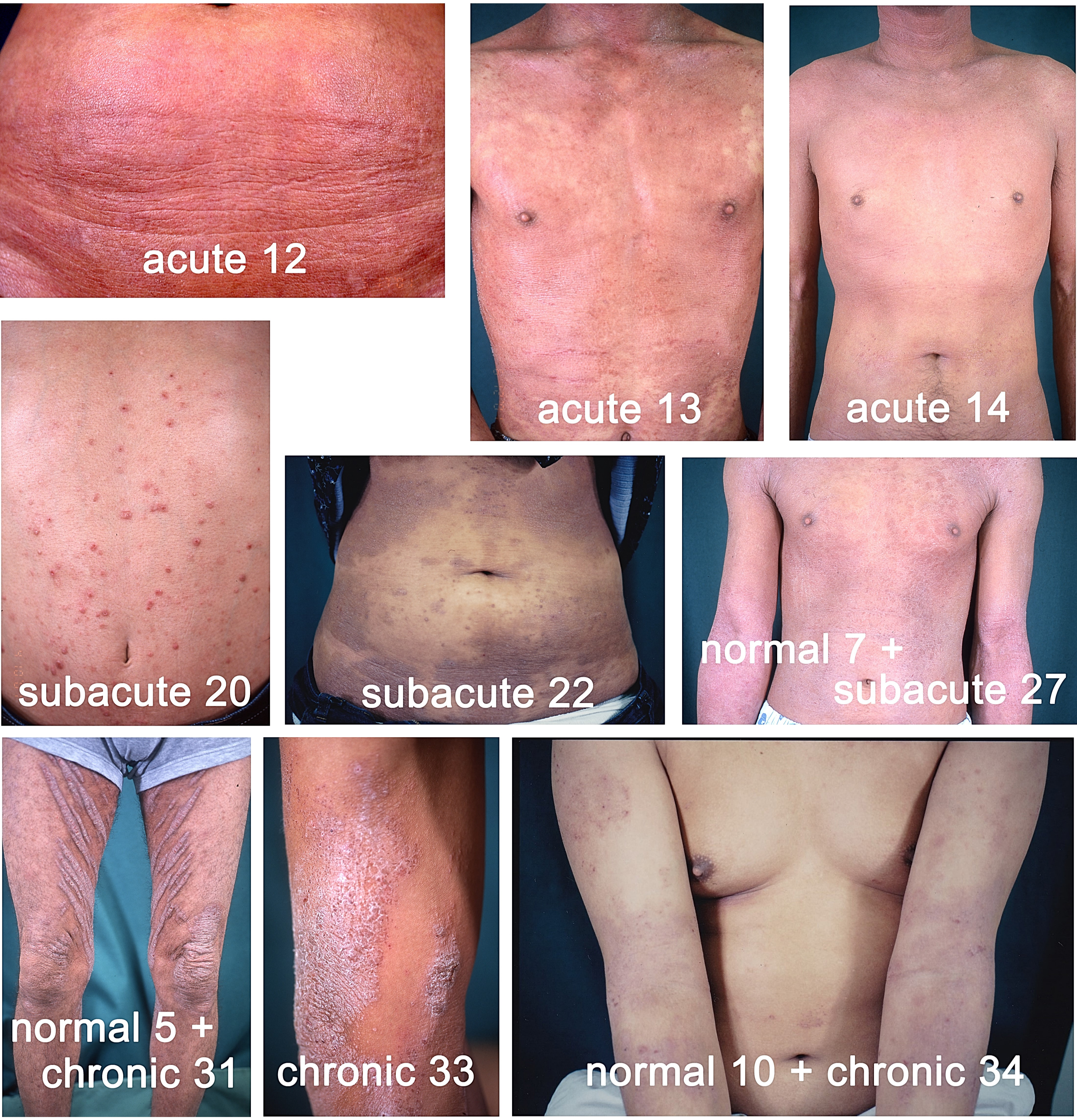

Figure S3. Representative Clinical Features of Patients with AD.

Clinical photographs captured at the time of skin biopsy are shown. The inserted letters indicate the skin status of the patient at the time of skin biopsy (non-lesioned to chronic) and the serial numbers of the biopsied specimens. The serial numbers of the specimens are used consistently in all figures and tables throughout the text.

Abbreviations: AD, atopic dermatitis

RNA PREPARATION, DNA MICROARRAY, AND STATISTICAL ANALYSIS

Total RNA was prepared from each biopsy specimen section using TRIzol (Invitrogen, Carlsbad, CA, USA) and an RNeasy Kit (Qiagen, Valencia, CA, USA). The derived RNA was amplified as described by Luo et al.[26] and used as a template for cRNA labelling. cRNA labelling and hybridization for the Gene Chip HGU133A arrays (Affymetrix, Santa Clara, CA, USA) were carried out as described in the Gene Chip Expression Analysis Technical Manual (Affymetrix). We analyzed 23,000 genes in 34 skin biopsy samples from 22 patients with atopic dermatitis (AD) and four normal controls using Affymetrix oligonucleotide arrays. Array images were quantified using the Micro Array Suite 5.0 software (Affymetrix), and the cell files were analyzed with the Affy 1.1 package (Bioconductor, http://www.bioconductor.org) to determine the expression levels. For background correction, normalization, probe-specific correction, and summarization, we used the algorithms mas, invariantset, pmonly, and liwong, respectively. The Mann–Whitney *U* test was performed to compare each gene between normal and AD samples; those that showed a significant difference ($P<0.05$) were selected.

The present study reexamined the data presented by Sugiura et al. in 2005.[27] The method of microarray analysis is the same as that previously used by Sugiura et al. In

the previous study, 22 cases were analyzed, but 17 cases with IgE ≥2,000 were selected. In the present study, we included all 22 cases. Microarray analysis was performed by Hayao Ebise MSc of Sumitomo Pharma Co., Ltd. (Osaka, Japan).

IMMUNOHISTOCHEMISTRY (IHC)

For IHC, we utilized the following 10 antibodies against HSP90B1 (heat shock protein 90 beta family member 1; anti-endoplasmin/HSP90B1; Lot: AP06150PU-N; 1:200 dilution; OriGene Technologies, Inc., Rockville, MD, USA), HSPA9 (heat shock protein family A (Hsp70) member 9; Grp75/Mot; 1:100 dilution; Abcam, Cambridge, UK), CANX (calnexin; Lot: ab22595; 1:100 dilution; Abcam), B2M (anti-β2 microglobulin; Lot: B2M/961; 1:100 dilution; Abcam), BGN (biglycan; Lot: ab231297; 1:48 dilution; Abcam), KLF2 (Kruppel like factor 2; Lot: ab203591; 1:200 dilution; Abcam), LEPR (leptin receptor; Lot: ab104403-100; 1:180 dilution; Abcam), NAB1 (NGFI-A binding protein 1; Lot: NBP2-46079; 1:150 dilution; Novus Biologicals, Centennial, CO, USA), EGR1 (early growth response 1; Lot: ab194357-100; 1:200 dilution; Abcam), and WIF1 (WNT inhibitory factor 1; Lot: ab71204-400; 1:40 dilution; Abcam).

IHC was performed using an automated *Roche* Ventana BenchMark XT staining system (Ventana Medical Systems/Roche, Tucson, AZ, USA). IHC was performed using an I-VIEW DAB Universal kit (760-041; Ventana Medical Systems/Roche) for antigen retrieval and detection conditions.

IMMUNOFLUORESCENCE ANALYSIS FOR CD4/CD8 DOUBLE STAINING

CD4 was stained using anti-CD4 antibody (rabbit) (Lot: GR3215096-1; 1:1,000 dilution; Abcam, Cambridge, UK) as the primary antibody and donkey anti-rabbit IgG antibody Cy3 (category number: AP182C; Lot: 3163509; 1:1,000 dilution; Merck Millipore, Burlington, MA, USA) as the secondary antibody.

CD8 was stained using anti-CD8 antibody (mouse) (Lot: GR3241658-13; 1:400 dilution; Abcam) as the primary antibody and donkey anti-mouse IgG H&L (Alexa Fluor® 488; Lot: 2018296; Life Technologies Corporation, Eugene, OR, USA) as the secondary antibody (1:1,000 dilution).

Table S4. The Probe Set of Microarray Analysis of 92 Genes with Significant Differences in Expression

Order	Cluster	Probe_set	Gene Symbol	Gene Title	Representative Public ID	RefSeq Transcript ID
1	1	204737_s_at	**MYH6 /// MYH7**	myosin, heavy chain 6, cardiac muscle, alpha /// myosin, heavy chain 7, cardiac muscle, beta	NM_000257	NM_000257 /// NM_002471 /// XR_245686
2	1	217576_x_at	**SOS2**	SOS Ras/Rho guanine nucleotide exchange factor 2	BF692958	NM_006939 /// XM_005268021 /// XM_011537103 /// XM_011537104
3	1	203336_s_at	**ITGB1BP1**	integrin beta 1 binding protein 1	AL548363	NM_004763 /// NM_022334 /// XM_005246183 /// XM_005246184 /// XM_005246185 /// XM_005246186 /// XM_005246187 /// XM_005246188 /// XM_005246189 /// XM_006711903 /// XM_011510416
4	1	214041_x_at	**RPL37A**	ribosomal protein L37a	BE857772	NM_000998
5	1	212336_at	**EPB41L1**	erythrocyte membrane protein band 4.1-like 1	AA912711	NM_001258329 /// NM_001258330 /// NM_001258331 /// NM_012156 /// NM_177996 /// XM_011528664 /// XM_011528665 /// XM_011528666 /// XM_011528667 /// XM_011528668 /// XM_011528669 /// XM_011528670 /// XM_011528671 /// XM_011528672 /// XM_011528673 /// XM_011528674 /// XM_011528675 /// XM_011528676 /// XM_011528677 /// XM_011528678 /// XM_011528679 /// XM_011528680 /// XM_011528681 /// XM_011528682 /// XM_011528683 /// XM_011528684 /// XM_011528685 /// XM_011528686
6	1	200806_s_at	**HSPD1**	heat shock 60kDa protein 1 (chaperonin)	BE256479	NM_002156 /// NM_199440 /// XM_005246518
7	1	201966_at	**NDUFS2**	NADH dehydrogenase (ubiquinone) Fe-S protein 2, 49kDa (NADH-coenzyme Q reductase)	NM_004550	NM_001166159 /// NM_004550 /// XM_005245208 /// XM_005245209
8	1	202414_at	**ERCC5**	excision repair cross-complementation group 5	NM_000123	NM_000123
9	1	214943_s_at	**ARID4B /// RBM34**	AT rich interactive domain 4B (RBP1-like) /// RNA binding motif protein 34	D38491	NM_001161533 /// NM_001206794 /// NM_015014 /// NM_016374 /// NM_031371 /// NR_027762 /// XM_005273160 /// XM_006711781 /// XM_011544133 /// XM_011544134 /// XM_011544212 /// XM_011544213 /// XR_949145
10	2	210051_at	**RAPGEF3**	Rap guanine nucleotide exchange factor 3	U78168	NM_001098531 /// NM_001098532 /// NM_006105 /// XM_005268571 /// XM_011537751 /// XM_011537752 /// XM_011537753 /// XM_011537754 /// XM_011537755 /// XM_011537756 /// XM_011537757 /// XM_011537758 /// XM_011537759 /// XR_944480
11	2	201496_x_at	**MYH11**	myosin, heavy chain 11, smooth muscle	S67238	NM_001040113 /// NM_001040114 /// NM_002474 /// NM_022844 /// XM_011522502 /// XM_011546698
12	2	201243_s_at	**ATP1B1**	ATPase, Na+/K+ transporting, beta 1 polypeptide	NM_001677	NM_001001787 /// NM_001677
13	2	209146_at	**MSMO1**	methylsterol monooxygenase 1	AV704962	NM_001017369 /// NM_006745 /// XM_005263176
14	2	206509_at	**PIP**	prolactin-induced protein	NM_002652	NM_002652
15	2	208962_s_at	**FADS1**	fatty acid desaturase 1	BE540552	NM_013402 /// XM_011545022
16	2	213693_s_at	**MUC1**	mucin 1, cell surface associated	AI610869	NM_001018016 /// NM_001018017 /// NM_001018021 /// NM_001044390 /// NM_001044391 /// NM_001044392 /// NM_001044393 /// NM_001204285 /// NM_001204286 /// NM_001204287 /// NM_001204288 /// NM_001204289 /// NM_001204290 /// NM_001204291 /// NM_001204292 /// NM_001204293 /// NM_001204294 /// NM_001204295 /// NM_001204296 /// NM_001204297 /// NM_002456 /// NM_182741
17	2	206799_at	**SCGB1D2**	secretoglobin, family 1D, member 2	NM_006551	NM_006551 /// XM_006718422
18	2	221418_s_at	**MED16**	mediator complex subunit 16	NM_005481	NM_005481 /// XM_011527604 /// XR_936134
19	2	203426_s_at	**IGFBP5**	insulin like growth factor binding protein 5	M65062	NM_000599
20	3	204734_at	**KRT15**	keratin 15, type I	NM_002275	NM_002275 /// XM_005257345 /// XM_011524784 /// XM_011524785

21	3	211458_s_at	**GABARAPL1 /// GABARAPL3**	GABA(A) receptor-associated protein like 1 /// GABA(A) receptors associated protein like 3, pseudogene	AF180519	NM_031412 /// NR_028287 /// XM_005253344
22	3	207961_x_at	**MYH11**	myosin, heavy chain 11, smooth muscle	NM_022870	NM_001040113 /// NM_001040114 /// NM_002474 /// NM_022844 /// XM_011522502 /// XM_011546698
23	3	217414_x_at	**HBA2**	hemoglobin, alpha 2	V00489	NM_000517
24	3	204018_x_at	**HBA1 /// HBA2**	hemoglobin, alpha 1 /// hemoglobin, alpha 2	NM_000558	NM_000517 /// NM_000558
25	3	211699_x_at	**HBA1 /// HBA2**	hemoglobin, alpha 1 /// hemoglobin, alpha 2	AF349571	NM_000517 /// NM_000558
26	3	211745_x_at	**HBA1 /// HBA2**	hemoglobin, alpha 1 /// hemoglobin, alpha 2	BC005931	NM_000517 /// NM_000558
27	3	209458_x_at	**HBA1 /// HBA2**	hemoglobin, alpha 1 /// hemoglobin, alpha 2	AF105974	NM_000517 /// NM_000558
28	3	212762_s_at	**TCF7L2**	transcription factor 7-like 2 (T-cell specific, HMG-box)	AI375916	NM_001146274 /// NM_001146283 /// NM_001146284 /// NM_001146285 /// NM_001146286 /// NM_001198525 /// NM_001198526 /// NM_001198527 /// NM_001198528 /// NM_001198529 /// NM_001198530 /// NM_001198531 /// NM_030756 /// XM_005270071 /// XM_005270073 /// XM_005270074 /// XM_005270075 /// XM_005270077 /// XM_005270078 /// XM_005270079 /// XM_005270080 /// XM_005270082 /// XM_005270083 /// XM_005270084 /// XM_005270085 /// XM_005270086 /// XM_005270088 /// XM_005270089 /// XM_005270091 /// XM_005270092 /// XM_005270093 /// XM_005270094 /// XM_005270095 /// XM_005270096 /// XM_005270100 /// XM_005270101 /// XM_005270102 /// XM_005270103 /// XM_005270104 /// XM_006717956 /// XM_011540109 /// XM_011540110 /// XM_011540111 /// XM_011540112 /// XM_011540113 /// XM_011540114 /// XM_011540115 /// XM_011540116 /// XM_011540117 /// XM_011540118 /// XM_011540119
29	4	213369_at	**CDHR1**	cadherin-related family member 1	AI825832	NM_001171971 /// NM_033100 /// XM_011540337 /// XM_011540338 /// XM_011540339 /// XM_011540340
30	4	219371_s_at	**KLF2**	Kruppel-like factor 2	NM_016270	NM_016270
31	4	206393_at	**TNNI2**	troponin I type 2 (skeletal, fast)	NM_003282	NM_001145829 /// NM_001145841 /// NM_003282
32	4	201693_s_at	**EGR1**	early growth response 1	AV733950	NM_001964
33	4	213089_at	**LOC100272216**	uncharacterized LOC100272216	AU158490	NR_027439
34	4	201261_x_at	**BGN**	biglycan	BC002416	NM_001711
35	4	213905_x_at	**BGN**	biglycan	AA845258	NM_001711
36	4	204712_at	**WIF1**	WNT inhibitory factor 1	NM_007191	NM_007191
37	4	215239_x_at	**ZNF273**	zinc finger protein 273	AU132789	NM_021148 /// NM_033548 /// NR_003099 /// XM_006715840 /// XM_006715841 /// XM_011515746 /// XM_011515747 /// XM_011515748 /// XM_011515749 /// XM_011515750 /// XR_927338
38	4	210495_x_at	**FN1**	fibronectin 1	AF130095	NM_001306129 /// NM_001306130 /// NM_001306131 /// NM_001306132 /// NM_002026 /// NM_054034 /// NM_212474 /// NM_212475 /// NM_212476 /// NM_212478 /// NM_212482 /// XM_005246397 /// XM_005246398 /// XM_005246399 /// XM_005246400 /// XM_005246401 /// XM_005246402 /// XM_005246403 /// XM_005246404 /// XM_005246405 /// XM_005246406 /// XM_005246407 /// XM_005246408 /// XM_005246409 /// XM_005246410 /// XM_005246411 /// XM_005246412 /// XM_005246413 /// XM_005246414 /// XM_005246415 /// XM_005246416 /// XM_005246417
39	4	209291_at	**ID4**	inhibitor of DNA binding 4, dominant negative helix-loop-helix protein	AW157094	NM_001546
40	4	207720_at	**LOR**	loricrin	NM_000427	NM_000427 /// XM_011509541
41	4	209894_at	**LEPR**	leptin receptor	U50748	NM_001003679 /// NM_001003680 /// NM_001198687 /// NM_001198688 /// NM_001198689 /// NM_002303

42	4	205559_s_at	**PCSK5**	proprotein convertase subtilisin/kexin type 5	NM_006200	NM_001190482 /// NM_006200 /// NR_120409 /// XM_005252039 /// XM_011518769 /// XM_011518770 /// XR_929806 /// XR_929807
43	4	201540_at	**FHL1**	four and a half LIM domains 1	NM_001449	NM_001159699 /// NM_001159700 /// NM_001159701 /// NM_001159702 /// NM_001159703 /// NM_001159704 /// NM_001167819 /// NM_001449 /// NR_027621 /// XM_006724743 /// XM_006724744 /// XM_006724745 /// XM_006724746 /// XM_006724747 /// XM_011531316
44	4	215704_at	**FLG**	filaggrin	AL356504	NM_002016 /// XM_011509329
45	4	202436_s_at	**CYP1B1**	cytochrome P450, family 1, subfamily B, polypeptide 1	AU144855	NM_000104
46	4	204021_s_at	**PURA**	purine-rich element binding protein A	NM_005859	NM_005859
47	5	208012_x_at	**SP110**	SP110 nuclear body protein	NM_004509	NM_001185015 /// NM_004509 /// NM_004510 /// NM_080424 /// XM_005246525 /// XM_006712487 /// XM_006712489 /// XM_011511088 /// XM_011511089 /// XM_011511090 /// XM_011511091 /// XM_011511092
48	5	217140_s_at	**VDAC1**	voltage-dependent anion channel 1	AJ002428	NM_003374 /// NR_036624 /// NR_036625 /// XM_005272075
49	5	208703_s_at	**APLP2**	amyloid beta (A4) precursor-like protein 2	BG427393	NM_001142276 /// NM_001142277 /// NM_001142278 /// NM_001243299 /// NM_001642 /// NR_024515 /// NR_024516
50	5	216231_s_at	**B2M**	beta-2-microglobulin	AW188940	NM_004048 /// XM_005254549 /// XM_006725182
51	5	200692_s_at	**HSPA9**	heat shock 70kDa protein 9 (mortalin)	NM_004134	NM_004134
52	5	214314_s_at	**EIF5B**	eukaryotic translation initiation factor 5B	BE138647	NM_015904
53	5	216988_s_at	**PTP4A2**	protein tyrosine phosphatase type IVA, member 2	L48722	NM_001195100 /// NM_001195101 /// NM_003479 /// NM_080391 /// NM_080392 /// XM_005271229 /// XM_005271230 /// XM_005271231 /// XM_005271232 /// XM_006710927 /// XM_006710928 /// XR_946766
54	5	212626_x_at	**HNRNPC**	heterogeneous nuclear ribonucleoprotein C (C1/C2)	AA664258	NM_001077442 /// NM_001077443 /// NM_004500 /// NM_031314 /// XM_006720125 /// XM_011536708 /// XM_011536709 /// XM_011536710 /// XM_011536711 /// XM_011536712
55	5	201241_at	**DDX1**	DEAD (Asp-Glu-Ala-Asp) box helicase 1	NM_004939	NM_004939
56	5	208047_s_at	**NAB1**	NGFI-A binding protein 1	NM_005966	NM_005966 /// XM_005246579 /// XM_005246580 /// XM_005246581 /// XM_005246582 /// XM_005246583 /// XM_005246585 /// XM_005246586 /// XM_005246587 /// XM_011511219 /// XM_011511220 /// XM_011511221 /// XM_011511222 /// XM_011511223
57	5	216449_x_at	**HSP90B1**	heat shock protein 90kDa beta (Grp94), member 1	AK025862	NM_003299
58	5	208853_s_at	**CANX**	calnexin	L18887	NM_001024649 /// NM_001746 /// XM_011534664 /// XM_011534665
59	5	208924_at	**RNF11**	ring finger protein 11	AB024703	NM_014372
60	5	200616_s_at	**MLEC**	malectin	BC000371	NM_001303627 /// NM_001303628 /// NM_014730 /// XM_011539031 /// XM_011539032 /// XM_011539033 /// XM_011539034
61	6	209720_s_at	**SERPINB3**	serpin peptidase inhibitor, clade B (ovalbumin), member 3	BC005224	NM_006919
62	6	204268_at	**S100A2**	S100 calcium binding protein A2	NM_005978	NM_005978
63	6	202345_s_at	**FABP5**	fatty acid binding protein 5 (psoriasis-associated)	NM_001444	NM_001444
64	6	204858_s_at	**TYMP**	thymidine phosphorylase	NM_001953	NM_001113755 /// NM_001113756 /// NM_001257988 /// NM_001257989 /// NM_001953
65	6	219850_s_at	**EHF**	ets homologous factor	NM_012153	NM_001206615 /// NM_001206616 /// NM_012153 /// XM_005252857 /// XM_005252860 /// XM_005252861 /// XM_005252862 /// XM_011519983 /// XM_011519984 /// XM_011519985
66	6	204750_s_at	**DSC2**	desmocollin 2	BF196457	NM_004949 /// NM_024422 /// XM_005258206
67	6	212657_s_at	**IL1RN**	interleukin 1 receptor antagonist	U65590	NM_000577 /// NM_173841 /// NM_173842 /// NM_173843 /// XM_005263661 /// XM_006712497 /// XM_011511121

68	6	203535_at	**S100A9**	S100 calcium binding protein A9	NM_002965	NM_002965
69	6	213680_at	**KRT6B**	keratin 6B, type II	AI831452	NM_005555
70	6	206166_s_at	**CLCA2**	chloride channel accessory 2	AF043977	NM_006536 /// XM_011542448
71	6	203964_at	**NMI**	N-myc (and STAT) interactor	NM_004688	NM_004688 /// XM_005246941
72	6	202917_s_at	**S100A8**	S100 calcium binding protein A8	NM_002964	NM_002964 /// XM_011509861
73	6	209800_at	**KRT16**	keratin 16, type I	AF061812	NM_005557
74	6	204580_at	**MMP12**	matrix metallopeptidase 12	NM_002426	NM_002426
75	6	201291_s_at	**TOP2A**	topoisomerase (DNA) II alpha	AU159942	NM_001067 /// XM_005257632 /// XM_011525165
76	6	214580_x_at	**KRT6A /// KRT6B /// KRT6C**	keratin 6A, type II /// keratin 6B, type II /// keratin 6C, type II	AL569511	NM_005554 /// NM_005555 /// NM_173086
77	6	214055_x_at	**PRRC2C**	proline-rich coiled-coil 2C	AW238632	NM_015172 /// XM_005245015 /// XM_005245016 /// XM_005245017 /// XM_005245018 /// XM_005245019 /// XM_005245020 /// XM_005245023 /// XM_005245024 /// XM_005245025 /// XM_005245026 /// XM_005245027 /// XM_005245028 /// XM_005245029 /// XM_005245030 /// XM_006711238 /// XM_011509343
78	6	219597_s_at	**DUOX1**	dual oxidase 1	NM_017434	NM_017434 /// NM_175940 /// XM_011521681 /// XM_011521682
79	6	205241_at	**SCO2**	SCO2 cytochrome c oxidase assembly protein	NM_005138	NM_001169109 /// NM_001169110 /// NM_001169111 /// NM_005138
80	6	217272_s_at	**SERPINB13**	serpin peptidase inhibitor, clade B (ovalbumin), member 13	AJ001698	NM_001307923 /// NM_012397 /// XM_005266705 /// XM_005266707 /// XM_011526029
81	6	209125_at	**KRT6A**	keratin 6A, type II	J00269	NM_005554
82	6	205180_s_at	**ADAM8**	ADAM metallopeptidase domain 8	NM_001109	NM_001109 /// NM_001164489 /// NM_001164490 /// XM_011539116 /// XM_011539117 /// XM_011539118
83	6	213796_at	**SPRR1A**	small proline-rich protein 1A	AI923984	NM_001199828 /// NM_005987
84	6	208965_s_at	**IFI16**	interferon, gamma-inducible protein 16	BG256677	NM_001206567 /// NM_005531 /// XM_005245127 /// XM_006711290 /// XM_011509478
85	6	220066_at	**NOD2**	nucleotide-binding oligomerization domain containing 2	NM_022162	NM_001293557 /// NM_022162 /// XM_005256084 /// XM_006721242 /// XM_006721243 /// XM_011523257 /// XM_011523258 /// XM_011523259 /// XM_011523260 /// XM_011523261 /// XR_429725 /// XR_429726 /// XR_933387
86	6	205916_at	**S100A7**	S100 calcium binding protein A7	NM_002963	NM_002963
87	6	211361_s_at	**SERPINB13**	serpin peptidase inhibitor, clade B (ovalbumin), member 13	AJ001696	NM_001307923 /// NM_012397 /// XM_005266705 /// XM_005266707 /// XM_011526029
88	6	221009_s_at	**ANGPTL4**	angiopoietin like 4	NM_016109	NM_001039667 /// NM_016109 /// NM_139314 /// NR_104213 /// XM_005272484 /// XM_005272485
89	6	202687_s_at	**TNFSF10**	tumor necrosis factor (ligand) superfamily, member 10	U57059	NM_001190942 /// NM_001190943 /// NM_003810 /// NR_033994
90	6	208650_s_at	**CD24**	CD24 molecule	BG327863	NM_001291737 /// NM_001291738 /// NM_001291739 /// NM_013230 /// NR_117089 /// NR_117090 /// XM_011535375
91	6	201091_s_at	**CBX3**	chromobox homolog 3	BE748755	NM_007276 /// NM_016587 /// XM_005249611 /// XM_005249612
92	6	200989_at	**HIF1A**	hypoxia inducible factor 1, alpha subunit (basic helix-loop-helix transcription factor)	NM_001530	NM_001243084 /// NM_001530 /// NM_181054

References

1. Kumbrink J, Kirsch KH, Johnson JP. EGR1, EGR2, and EGR3 activate the expression of their coregulator NAB2 establishing a negative feedback loop in cells of neuroectodermal and epithelial origin. J Cell Biochem 2010;111(1):207-17.

2. Schaeffer L, Gohlke H, Muller M, et al. Common genetic variants of the FADS1 FADS2 gene cluster and their reconstructed haplotypes are associated with the fatty acid composition in phospholipids. Hum Mol Genet 2006;15(11):1745-56.

3. Lattka E, Illig T, Heinrich J, Koletzko B. FADS gene cluster polymorphisms: important modulators of fatty acid levels and their impact on atopic diseases. J Nutrigenet Nutrigenomics 2009;2(3):119-28.

4. Li F, Zhu W, Gonzalez F. Potential role of CYP1B1 in the development and treatment of metabolic diseases. J Pharmacol Ther 2017;178:18-30.

5. Uehara M, Sugiura H, Tanaka K. Rarity of hypertension in adult patients with atopic dermatitis. Br J Dermatol 2002;146(4):631-5.

6. Sator PG, Schmidt JB, Hönigsmann H. Comparison of epidermal hydration and skin surface lipids in healthy individuals and in patients with atopic dermatitis. J Am Acad Dermatol 2003;48(3):352-8.

7. Sugiura S, Fujimiya M, Ebise H, et al. Immunosuppressive effect of

prolactin-induced protein: a new insight into its local and systemic role in chronic allergic contact dermatitis. Br J Dermatol 2010;162(6):1286-93.

8. Sugiura S, Tazuke M, Ueno S, et al. Effect of prolactin-induced protein on human skin: new insight into the digestive action of this aspartic peptidase on the stratum corneum and its induction of keratinocyte proliferation. J Invest Dermatol. 2015;135(3):776-85.

9. Hirota T, Takahashi T, Kubo M, et al. Genome-wide association study identifies eight new susceptibility loci for atopic dermatitis in the Japanese population. Nat Genet 2012;44(11):1222-6.

10. Kuo CT, Veselits ML, Leiden JM. LKLF: A transcriptional regulator of single-positive T cell quiescence and survival. Science 1997;277:1986-90.

11. Leri M, Bemporad F, Oropesa-Nuñez R, et al. Molecular insights into cell toxicity of a novel familial amyloidogenic variant of β2-microglobulin. J Cell Mol Med 2016;20(8):1443-56.

12. Tuli A, Sharma M, Wang X, et al. Amyloid precursor-like protein 2 association with HLA class I molecules. Cancer Immunol Immunother 2009;58(9):1419-31.

13. Kaul SC, Wadhwa R, Matsuda Y, et al. Mouse and human chromosomal assignments of mortalin, a novel member of the murine hsp70 family of proteins. FEBS

Lett 1995;361(2-3):269-72.

14. Le Beau MM, Espinosa R 3rd, Neuman WL, et al. Cytogenetic and molecular delineation of the smallest commonly deleted region of chromosome 5 in malignant myeloid diseases. Proc Natl Acad Sci U S A. 1993;90(12):5484-8.

15. Ma ZW, Pejovic T, Najfeld V, Ward DC, Johnson EM. Localization of PURA, the gene encoding the sequence-specific single-stranded-DNA-binding protein Pur alpha, to chromosome band 5q31. Cytogenet Cell Genet 1995;71(1):64-7.

16. Swirnoff AH, Apel ED, Svaren J, et al. Nab1, a corepressor of NGFI-A (Egr-1), contains an active transcriptional repression domain. Mol Cell Biol 1998;18(1):512-24.

17. Moser D, Molitor A, Kumsta R, Tatschner T, Riederer P, Meyer J. The glucocorticoid receptor gene exon 1-F promoter is not methylated at the NGFI-A binding site in human hippocampus. World J Biol Psychiatry 2007;8(4):262-8.

18. Reijnders MRF, Leventer RJ, Lee BH, et al. PURA-related neurodevelopmental disorders. 2017 Apr 27. In: Adam MP, Ardinger HH, Pagon RA, Wallace SE, Bean LJH, Stephens K, Amemiya A, editors. GeneReviews® [Internet]. Seattle (WA): University of Washington, Seattle, 1993-2020.

19. Fagerberg L, Hallström BM, Oksvold P, et. al. Analysis of the human tissue-specific expression by genome-wide integration of transcriptomics and antibody-based

proteomics. Mol Cell Proteomics 2014;13(2):397-406. doi:10.1074/mcp.M113.035600.

20. Schlüter H, Stark HJ, Sinha D, Boukamp P, Kaur P. WIF1 is expressed by stem cells of the human interfollicular epidermis and acts to suppress keratinocyte proliferation. J Invest Dermatol 2013;133:1669-73.

21. Jin S, Park CO, Shin JU, et al. DAMP molecules S100A9 and S100A8 activated by IL-17A and house-dust mites are increased in atopic dermatitis. Exp Dermatol 2014;23(12):938-41. doi:10.1111/exd.12563.

22. Chen GY, Tang J, Zheng P, Liu Y. CD24 and Siglec-10 selectively repress tissue damage-induced immune responses. Science 2009;323(5922):1722-5

23. Xiahou Z, Wang X, Shen J, et al. NMI and IFP35 serve as proinflammatory DAMPs during cellular infection and injury. Nat Commun 2017;8(1):950. doi:10.1038/s41467-017-00930-9.

24. Ghoreishi M, Yokozeki H, Hua WM, Nishioka K. Expression of 27 KD, 65 KD and 72/73 KD heat shock protein in atopic dermatitis: comparison with those in normal skin and contact dermatitis. J Dermatol 2000; 27: 370–379

25. Scieglinska D, Krawczyk Z, Sojka DR, Gogler-Pigłowska A. Heat shock proteins in the physiology and pathophysiology of epidermal keratinocytes. Cell Stress Chaperones 2019;24(6):1027-44.

26. Luo L, Salunga RC, Guo H, et al. Gene expression profiles of laser-captured adjacent neuronal subtypes. Nature Med 1995;5(1):117-22.

27. Sugiura H, Ebise H, Tazawa T, et al. Large-scale DNA microarray analysis of atopic skin lesions shows overexpression of an epidermal differentiation gene cluster in the alternative pathway and lack of protective gene expression in the cornified envelope. Br J Dermatol 2005;152:146-9.
